# A Digital Cognitive Behavioral Therapy Program for Adults With Alcohol Use Disorder: A Randomized Clinical Trial

**DOI:** 10.1001/jamanetworkopen.2024.35205

**Published:** 2024-09-26

**Authors:** Brian D. Kiluk, Bryan Benitez, Elise E. DeVito, Tami L. Frankforter, Donna M. LaPaglia, Stephanie S. O’Malley, Charla Nich

**Affiliations:** 1 Yale School of Medicine, New Haven, Connecticut

## Abstract

**Importance:**

Cognitive behavioral therapy (CBT) is an evidence-based treatment for alcohol use, yet patient access is limited and may be enhanced through digital therapeutics.

**Objective:**

To evaluate the efficacy of a digital CBT program (Computer-Based Training for Cognitive Behavioral Therapy [CBT4CBT]) or clinician-delivered CBT compared with standard treatment for reducing alcohol use.

**Design, Setting, and Participants:**

A 3-arm randomized clinical trial was conducted at outpatient substance use treatment facilities in Connecticut between February 14, 2017, and December 31, 2021, that included an 8-week treatment period with a 6-month follow-up period. Treatment-seeking adults were included who met criteria for current alcohol use disorder and reported drinking at least 14 (men) or 7 (women) drinks per week in the past month and were sufficiently stable for outpatient treatment.

**Interventions:**

Participants were randomly assigned to 1 of the following groups: (1) treatment as usual (TAU) consisting of weekly group or individual counseling, (2) CBT delivered weekly by trained and fidelity-monitored clinicians, or (3) web-based CBT plus brief weekly clinical monitoring.

**Main Outcomes and Measures:**

Rates of alcohol use were measured weekly during the treatment period and at 1-, 3-, and 6-month follow-up using the timeline follow-back method. The primary outcome was the percentage of days abstinent (PDA) from alcohol per month. Intention-to-treat analyses were conducted.

**Results:**

Of the 99 randomized participants (mean [SD] age, 45.5 [12.7]), 66 were male (66.7%); 39 identified as Black/African American (39.8%), 19 (19.2%) as Hispanic, and 47 (48.0%) as White. Mean (SD) rates of PDA from baseline to 6-month follow-up were 49.3% (27.8%) to 69.6% (34.4%) for TAU; 53.7% (29.8%) to 70.2% (35.1%) for CBT; and 47.6% (31.8%) to 82.6% (25.3%) for digital CBT. Results of random-effects regression showed a significant increase in PDA during the study period, with those assigned to digital CBT increasing PDA at a faster rate than TAU (*t*_733_ = 2.55; *P* = .01) and CBT (*t*_733_ = 3.36; *P* < .001). However, there was no statistically significant difference between treatment groups during the 8-week treatment period.

**Conclusions and Relevance:**

In this randomized clinical trial, while there was no significant difference between treatment groups during the 8-week treatment period, there was differential change between treatments during the 8-month study period that provides support for the efficacy of this digital CBT program.

**Trial Registration:**

ClinicalTrials.gov Identifier: NCT02742246

## Key Points

**Question:**

Is the digital cognitive behavioral therapy (CBT) program evaluated in this study more efficacious than standard outpatient care at improving the percentage of days abstinent in patients seeking treatment for alcohol use disorder?

**Findings:**

In this randomized clinical trial with 99 participants seeking treatment for alcohol use disorder, those assigned to digital CBT plus weekly monitoring increased their percentage of days abstinent by more than 50% during the 8-month study period. However, there was no statistically significant difference between groups during the treatment period.

**Meaning:**

The results of this trial provide support for the efficacy of a digital CBT program with brief weekly clinical monitoring for individuals seeking treatment for alcohol use.

## Teaser

This randomized clinical trial evaluates the use of a digital version of generalized cognitive behavioral therapy strategies in the treatment of individuals with alcohol use disorder.

## Introduction

Alcohol use disorder (AUD), characterized by an impaired ability to stop or control alcohol use despite adverse consequences, ranks as one of the most prevalent mental disorders globally and is associated with high mortality and disease burden.^1^ Implementation of evidence-based psychotherapies for treating AUD, such as cognitive behavioral therapy (CBT),^2,3^ into community practice settings has been challenging.^4,5^ Marked by low fidelity to the established intervention,^6^ many patients seeking treatment may not in fact be receiving the true intervention that has been shown to be effective.^7,8^ Multicomponent training improves therapist knowledge and delivery,^9,10^ but is too costly and time-intensive for most settings.

Digital therapeutics (mobile, web, or other software-based platforms that deliver treatments for medical conditions or diseases) offer the potential to enhance the ability of patients to access evidence-based treatment for AUD.^11,12,13^ They also offer major advantages of standardization and consistent quality, reduction of cost and clinician time, and potential continuous availability. However, evidence supporting the efficacy of digital therapeutics through randomized clinical trials (RCTs) is needed ensure these tools can benefit people with AUD.^14^

A digital version of CBT, called Computer-Based Training for Cognitive Behavioral Therapy (CBT4CBT), was developed in early 2000s by the late Kathleen Carroll, PhD, and colleagues.^15^ This digital CBT program is a web-based program consisting of core skill topics (modules) designed to teach patients generalizable CBT strategies for avoiding drug use,^16^ with a subsequent version targeting alcohol use,^17^ via video, graphics, audio instruction, and interactive exercises.^18^ Patient improvement in coping skills, cognitive control, and response inhibition have been identified as potential mechanisms of the effectiveness of this digital CBT program.^19,20,21^ The alcohol version of the program follows the same format used in the original, with content adapted based on the National Institute on Alcohol Abuse and Alcoholism CBT manual.^22^ Results from a pilot RCT in a sample of 68 adults with AUD indicated that this digital CBT program added to standard outpatient treatment was superior to standard treatment alone at improving alcohol abstinence; also, outcomes from digital CBT delivered as a stand-alone program with weekly clinical monitoring were not significantly different from standard treatment.^17^

The purpose of the current study was to further evaluate the efficacy of the digital CBT program for AUD as a stand-alone intervention compared with standard treatment in a larger RCT. The study also evaluated a clinician-delivered version of CBT to inform design and power calculations for a future noninferiority trial of digital vs clinician-delivered CBT. The primary hypothesis was that each form of CBT would be more effective than standard treatment at increasing alcohol abstinence.

## Methods

### Overview

**Figure 1.**
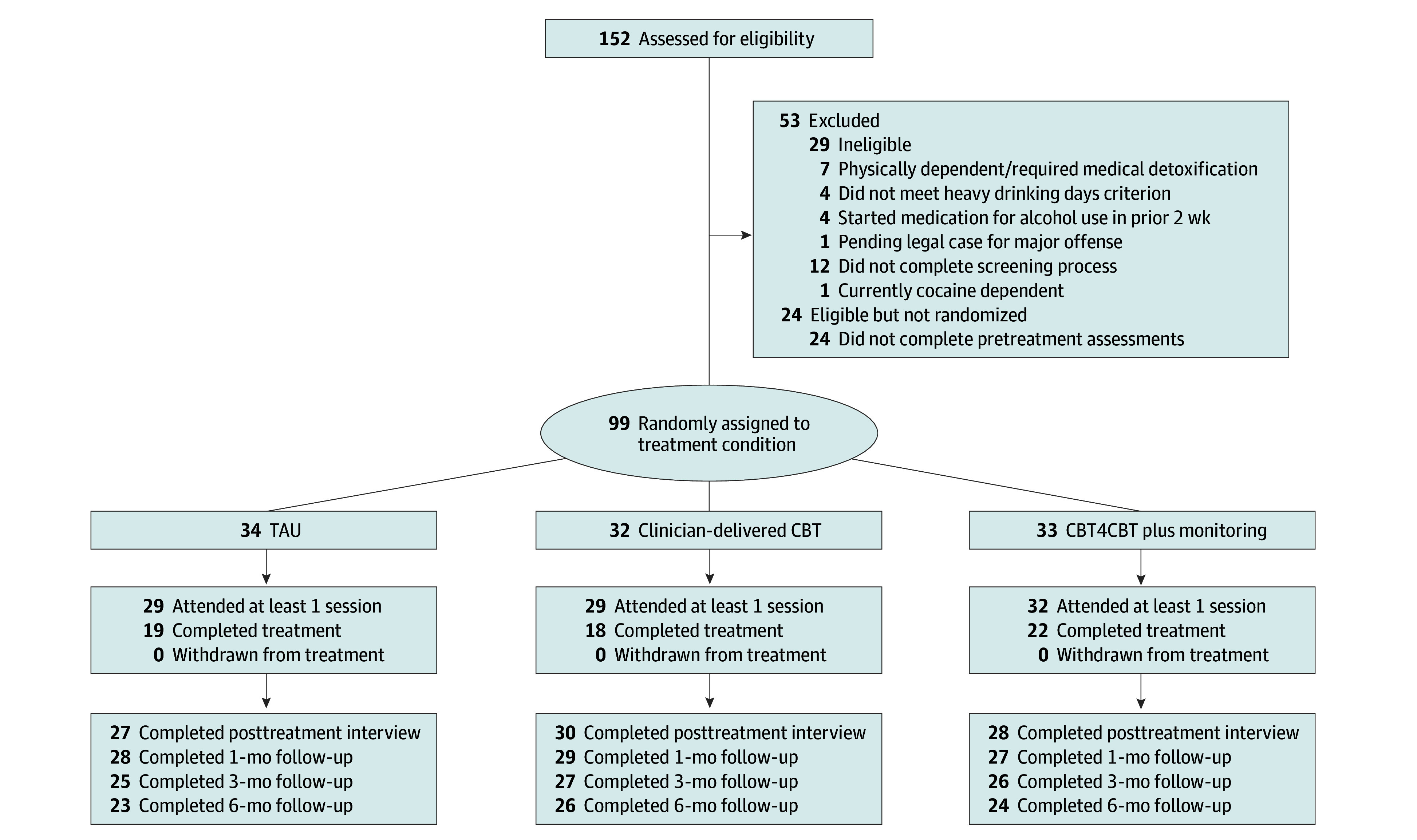
Participant Flow CBT indicates cognitive behavioral therapy; CBT4CBT, Computer-Based Training for Cognitive Behavioral Therapy; TAU, treatment as usual.

In this 3-arm, parallel-group RCT, conducted between February 14, 2017, and December 31, 2021 (end of funding), individuals seeking outpatient treatment for AUD were randomly assigned to 1 of the following treatments: (1) treatment as usual (TAU), (2) clinician-delivered CBT, or (3) digital CBT with brief clinical monitoring. Frequency of alcohol use was measured weekly during an 8-week treatment period and at 1-, 3-, and 6-month follow-up interviews. The primary outcome was the change in percentage of days abstinent (PDA) by month during treatment and through the 6-month follow-up period. The trial was approved by the Yale University Institutional Review Board. This report follows the Consolidated Standards of Reporting Trials (CONSORT) reporting guideline for reporting parallel group randomized trials.^23^ The full trial protocol can be accessed in Supplement 1. A total of 152 individuals provided written informed consent and were screened for eligibility (Figure 1). Participants received financial compensation in the form of gift cards for completion of assessments; the maximum compensation available was $655.

### Participants

Participants were recruited from outpatient substance use treatment facilities in Connecticut. Eligible individuals met the following criteria: (1) age 18 years or older, (2) current *Diagnostic and Statistical Manual of Mental Disorders, Fifth Editio*n (*DSM-5*) criteria for AUD and reported drinking 14 (men) or 7 (women) or more drinks weekly with 4 or more heavy drinking days (>4 drinks for males; >3 drinks for females) in the past month, (3) sufficiently stable for outpatient treatment, and (4) fluent in English and 6th grade or higher reading level. Exclusion criteria included (1) untreated bipolar or schizophrenic disorder, (2) current legal case pending such that incarceration was probable, or (3) prescribed an alcohol pharmacotherapy in the prior 2 weeks. Power analyses indicated a sample size of 60 per condition was needed to detect a small to medium effect (*d* = 0.35) of CBT or digital CBT compared with TAU on PDA, using a longitudinal random-effects regression model (2 groups, PDA by month, α = 0.05, β = 0.80).

Ninety-nine individuals were eligible for participation and randomly assigned to treatment by a research coordinator in equal numbers using a computerized urn randomization program^24^ to balance groups with respect to sex; racial and ethnic minority; educational level; severity of alcohol use as assessed by the Alcohol Use Disorders Identification Test (AUDIT)^25^; and whether referred to treatment by the criminal justice system. Racial and ethnicity data were included to ensure equal intervention allocation with respect to demographic variables.

### Treatments

#### Treatment as Usual 

Participants received standard outpatient treatment, consisting of weekly group and/or individual counseling delivered by certified counselors. All participants were offered standard ancillary services provided at the treatment facility, which included psychiatric, pharmacologic, and emergency services.

#### Clinician-Delivered CBT

Participants received weekly individual sessions of manual-guided CBT^16,22^ delivered by clinicians trained and supervised to protect internal validity and treatment integrity.^26^ There were 12 clinicians, 67% of whom were female. Following didactic training, clinicians completed supervised training cases with detailed feedback, based on ratings of audio-recorded sessions using the Yale Adherence and Competence Scale.^27^ Clinicians met weekly with a CBT expert for coaching and supervision during the trial. Audio recordings from 55 in-person sessions (40%) were randomly selected and rated, which demonstrated adequate CBT adherence and competence.

#### Digital CBT Plus Brief Clinical Monitoring

Participants were asked to complete 1 digital CBT module per week, accessed on a computer within the clinic or off-site with their own device (cellular data–enabled tablets were provided during the COVID-19 pandemic). The digital CBT program consists of 7 modules covering basic CBT concepts (eg, drink refusal skills, coping with craving), each structured to parallel clinician-delivered CBT sessions. In addition, participants were provided with brief (approximately 10 minutes) clinical monitoring weekly in person or remotely by a clinician that was manual guided^28^ and followed guidelines for low-intensity interventions.^29,30^

### Assessments

Assessments were administered at baseline, weekly during the treatment, at the end of the 8-week treatment period, and at 1-, 3-, and 6-month follow-up interviews. The Structured Clinical Interview for *DSM-5* was used to determine eligibility.^31^ Hazardous drinking was measured with the AUDIT^25,32^ before randomization. AUDIT scores represent the level of risky alcohol consumption over the past year. Total scores range from 0 to 40; scores from 1 to 7 suggest low-risk alcohol consumption, and scores of 15 or higher indicate the likelihood of moderate to severe alcohol use disorder. Self-reported frequency and quantity of alcohol and drug use was assessed with a calendar-based Timeline Follow Back^33^ to derive PDA and a secondary outcome, the percentage of heavy drinking days (PHDD). Urine samples were collected at each in-person visit, which were tested for ethyl glucuronide (EtG) as a biological indicator of recent heavy drinking (cutoff 500 ng/mL).^34,35,36,37,38^ Assessment of potential treatment mediators included a brief version of the Coping Strategies Scale^39,40^ to assess frequency of coping strategies for avoiding alcohol use, as well as a 35-item multiple choice instrument to assess knowledge of CBT concepts.^41^

### Statistical Analysis

All analyses were performed using SPSS, version 29.0.1.0 (IBM Inc) and SAS, version 9.4 (SAS Institute LLC). Statistical significance for 2-sided tests was set at *P* < .05. Analysis of variance or χ^2^ test was used to evaluate demographic and baseline characteristics, as well as treatment adherence, across treatment conditions. Engagement and usability data regarding the digital CBT program included the mean number of modules completed, the mean amount of time to complete each module, and the number of homework assignments completed.

The principal analytic strategy for evaluating treatment effects on PDA was longitudinal random-effects regression analysis using the maximum likelihood approach for handling missing data, with time modeled by month during the treatment period and through the 6-month follow-up. Following an omnibus test modeled with all 3 treatments, 2 a priori planned contrasts were evaluated: TAU vs clinician-delivered CBT and TAU vs digital CBT plus brief monitoring. We added a third contrast post hoc to evaluate clinician-delivered CBT vs digital CBT plus brief monitoring. All analyses were conducted for the full intention-to-treat (ITT) sample, using all available data. Separate analyses were conducted for the subsample of participants who were deemed treatment completers (defined a priori as attending at least 5 sessions/modules within 8 weeks). Secondary outcomes included PHDD, the percentage of negative EtG urine test results, change in coping strategies, and CBT knowledge.

## Results

### Participants

Demographic and baseline characteristics were comparable across treatments (Table). Most participants self-reported their gender as male (67.7%; female, 33.3%), and the percentages of self-reported race and ethnicity, with missing data on 1 individual, were Black/African American (39.8%), Hispanic only (8.2%), White (48.0%), multiracial (3.1%), and other (African European, 1.0%). Mean (SD) age was 45.5 (12.7) years. Most participants were unemployed (59.6%), not married/single (78.4%), and completed high school (90.9%). Thirty-four participants (34.3%) indicated they were referred to treatment by the criminal justice system. Participants reported drinking a mean (SD) of 13.8 (8.5) days during the month before randomization, with heavy drinking on 9.6 (8.5) days, and 7.4 (5.7) drinks per drinking day. The mean (SD) AUDIT score was 22.0 (8.1).

**Table.** Baseline Demographic and Clinical Characteristics

Variable	TAU (n = 34)	CBT (n = 32)	Digital CBT (n = 33)	Total (n = 99)
**Categorical variables, No. (%)**				
Gender				
Male	22 (64.7)	25 (78.1)	19 (57.6)	66 (66.7)
Female	12 (35.3)	7 (21.9)	14 (42.4)	33 (33.3)
Hispanic ethnicity^a^	9 (26.5)	3 (9.4)	7 (21.2)	19 (19.2)
Race				
White	13 (39.4)	17 (53.1)	17 (51.5)	47 (48.0)
Black/African American	16 (48.5)	13 (40.6)	10 (30.3)	39 (39.8)
Hispanic only^a^	3 (9.1)	1 (3.1)	4 (12.1)	8 (8.2)
Multiracial	1 (3.0)	1 (3.1)	1 (3.0)	3 (3.1)
Other (African European)	0	0	1 (3.0)	1 (1.0)
Completed high school	32 (94.1)	31 (96.9)	27 (81.8)	90 (90.9)
Never married/living alone	27 (79.4)	24 (77.4)	25 (78.1)	76 (78.4)
Unemployed	21 (61.8)	17 (53.1)	21 (63.6)	59 (59.6)
Alcohol use disorder severity				
Mild	3 (8.8)	1 (3.2)	1 (3.0)	5 (5.1)
Moderate	8 (23.5)	7 (22.6)	7 (21.2)	22 (22.4)
Severe	23 (67.6)	23 (74.2)	25 (75.8)	71 (72.4)
**Continuous variables, mean (SD)**				
Age, y	44.5 (14.0)	45.3 (12.6)	46.8 (11.6)	45.5 (12.7)
Days of alcohol use, past 28	14.2 (7.8)	12.8 (8.6)	14.5 (9.2)	13.8 (8.5)
Heavy drinking days, past 28	8.9 (8.1)	9.1 (8.4)	10.8 (9.0)	9.6 (8.5)
Drinks per drinking day past 28	6.3 (4.0)	7.1 (4.0)	8.7 (8.0)	7.4 (5.7)
PDA, past 28 d, %	49.3 (27.8)	53.7 (29.8)	47.6 (31.8)	50.1 (29.7)
PHDD, past 28 d, %	68.2 (28.9)	67.5 (30.1)	61.4 (32)	65.7 (30.2)
AUDIT score^b^	20.7 (9.0)	21.4 (8.6)	23.9 (6.3)	22.0 (8.1)

Abbreviations: AUDIT, Alcohol Use Disorder Identification Test; CBT, cognitive behavioral therapy; PDA, percentage of days abstinent; PHDD, percentage of heavy drinking days; TAU, treatment as usual.

^a^ Hispanic race represents those who self-reported only Hispanic as their race.

^b^ AUDIT scores represent the level of risky alcohol consumption over the past year. Total scores range from 0 to 40; scores from 1 to 7 suggest low-risk alcohol consumption. Scores of 15 or higher indicate the likelihood of moderate to severe alcohol use disorder.

### Treatment Engagement, Retention, and Safety

Although we planned to enroll 180 participants, we randomized 99 participants due to the COVID-19 pandemic. Of 90 who initiated treatment, 59 (65.6%) completed the treatment protocol (Figure 1). The percentage of participants who initiated or completed treatment was comparable across conditions (TAU, 21 [61.8%]; CBT, 16 [50.0%]; digital CBT, 22 [66.7%]). When the COVID-19 pandemic shutdowns started in the US (March 2020), 83 participants had already finished the study treatment period; 7 were active when all research and treatment visits transitioned to telephone or video-based teleconference and 9 enrolled after March 2020. COVID-19 impacted research and treatment visits for 16 participants: TAU, 5 (14.7%); CBT, 6 (18.8%); and digital CBT, 5 (15.2%). There were 16 adverse events reported during the trial, nearly all due to alcohol-related withdrawal (n = 7) or medical detoxification (n = 7). Rates of adverse events did not differ substantially between TAU and digital CBT.

Participants spent a mean (SD) of 37.6 (18.5) days in treatment of the 56-day period, with no significant difference between treatments. Participants assigned to TAU attended a mean (SD) of 4.8 (3.1) counseling sessions, those assigned to CBT attended 4.5 (2.3) sessions, and those assigned to digital CBT completed 5.2 (2.1) modules and attended 4.7 (2.1) monitoring sessions. The number of sessions attended during the treatment period did not differ significantly between groups. Engagement and usability data regarding digital CBT indicated participants spent 36 (7.1) minutes on each module and completed 3 of 6 homework assignments, which was comparable to prior studies.^17,41^

### Treatment Effects on Primary Outcome

The eTable in Supplement 2 provides the mean PDA at each time point across treatments. Mean (SD) rates of PDA by month from baseline to week 8 were TAU, 49.3% (27.8%) to 69.3% (26.2%); CBT, 53.7% (29.8%) to 68.1% (29.9%); and digital CBT, 47.6% (31.8%) to 75.1% (25.1%). In the ITT sample, results of random-effects regression indicated a significant increase in PDA by month during the 8-week treatment period (*b* = 10.88; *F*_1, 183.45_ = 66.2; *P* < .001), but no differential change between treatments. Overall, participants reported a mean (SD) PDA of 50.1% (29.7%) at baseline, 68.1% (29.1%) at week 4, and 70.9% (27.0%) at week 8. Among treatment completers, results indicated a similar pattern with a significant increase in PDA over time (*b* = 11.03; *F_1_*_, 116.98_ = 47.75; *P* < .001), but no differential rate of change between treatment conditions.

Results during the 6-month follow-up period for the ITT sample did not indicate a change in PDA over time. However, among treatment completers, results indicated a significant interaction of time × treatment (*F*_2, 312.21_ = 8.01; *P* < .001); post hoc contrasts indicated that PDA decreased during follow-up for those who completed clinician CBT compared with those who completed digital CBT (*b* = −4.85; *t*_301_ = 3.96; *P* < .001) or TAU (*b* = −3.36; *t*_301_ = 2.64; *P* = .009). From the end of treatment to 6-month follow-up, the mean PDA for those who completed CBT decreased from 72.5% to 61.2%, compared with a change from 76.6% to 85.9% for those who completed digital CBT.

**Figure 2.**
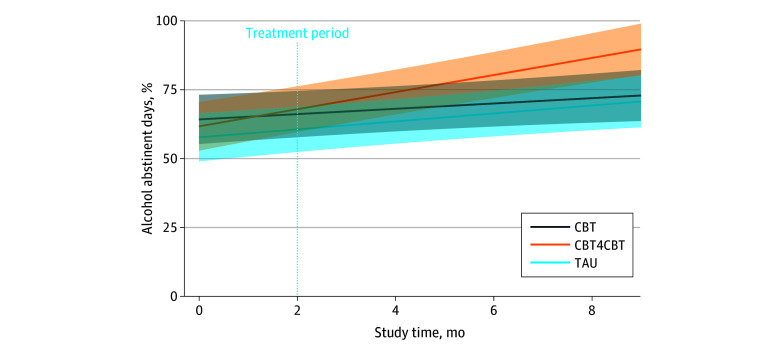
Percentage of Days Abstinent (PDA) From Alcohol by Month During Study Period Lines represent slope of change in PDA by month; shaded areas represent 95% CIs. CBT indicates cognitive behavioral therapy; CBT4CBT, Computer-Based Training for Cognitive Behavioral Therapy; TAU, treatment as usual.

Across the full 8-month study period, there were statistically significant time × treatment interactions for PDA in the ITT sample (*F*_2, 733_ = 6.12; *P* = .002) and treatment completers subsample (*F*_2, 477_ = 14.82; *P* < .001). Mean (SD) rates of PDA from baseline to 6-month follow-up were TAU, 49.3% (27.8%) to 69.6% (34.4%); CBT, 53.7% (29.8%) to 70.2% (35.1%); and digital CBT, 47.6% (31.8%) to 82.6% (25.3%). Post hoc comparisons showed digital CBT increased PDA over time at a faster rate than TAU (*b* = 1.66; *t*_733_ = 2.55; *P* = .01) and CBT (*b* = 2.14; *t*_733_ = 3.36; *P* < .001) (Figure 2). Among treatment completers, digital CBT increased PDA at a faster rate than TAU (*b* = 2.21; *t*_477_ = 2.98; *P* = .003) and CBT (*b* = 4.12; *t*_477_ = 5.41; *P* < .001), while TAU also increased PDA at a faster rate than CBT (*b* = 1.92; *t*_477_ = 2.43; *P* = .02).

### Secondary Outcomes: Alcohol Use, Coping Skills, and CBT Knowledge

In terms of PHDD, the pattern of results was similar to PDA, indicating a significant decrease in PHDD during the 8-week treatment period (*b* = −26.50; *F*_1, 277_ = 174.5; *P* < .001), but no differential change between treatments. The eTable in Supplement 2 reports the mean (SD) PHDD at each time point. Overall, participants reported a mean PHDD of 65.7% (30.2%) at baseline, 15.1% (20.9%) at week 4, and 13.8% (21.8%) at week 8. This reduction was also true for treatment completers. There was no significant change in PHDD over time during the 6-month follow-up period for the full ITT sample or for the subsample of treatment completers.

Results indicated no significant difference between treatments in the percentage of negative EtG test results during the treatment period. The mean (SD) percentage of negative EtG test results (of the number of samples collected) was 49.2% (37.3%). Participants submitted a mean (SD) 5.9 (2.6) urine samples (of 8 expected); the number submitted were not significantly different by treatment.

There was a significant change over time on mean Coping Strategies Scale scores between baseline and the end of treatment (*F*_1, 68_ = 13.10; *P* < .001) and differential change over time by treatment (*F*_2, 68_ = 3.56; *P* < .05). Those assigned to digital CBT reported the greatest increase in frequency of coping strategies from baseline to the end of treatment (week 0: mean, 36.2; week 8: mean, 49.2) compared with TAU (week 0: mean, 38.0; week 8: mean, 45.0) or CBT (week 0: mean, 39.8; week 8: mean, 40.6). The CBT knowledge scores also showed a differential change from baseline to 6-month follow-up (*F*_4, 102_ = 2.6; *P* < .05); those assigned to digital CBT showed the greatest increase in knowledge scores, from 47% to 60% correct.

## Discussion

To our knowledge, this is one of the first studies to evaluate the efficacy of a digital CBT program for alcohol use within an RCT that also evaluated a clinician-delivered CBT treatment in comparison with standard outpatient treatment for individuals with AUD. During the trial, in the full sample, regardless of treatment group, participants demonstrated a significant increase in alcohol abstinence as well as a decrease in heavy drinking days over time. Although differences in alcohol abstinence between treatments were not apparent when the treatment and follow-up periods were examined separately, when the entire 8-month study period was considered, those assigned to digital CBT provided with brief weekly monitoring showed a greater increase in days abstinent from alcohol compared with standard treatment or clinician-delivered CBT. This is consistent with findings supporting the efficacy of this digital CBT program in an outpatient sample of individuals with various nonalcohol substance use disorders.^41^ Secondary outcomes measuring the use of coping skills and CBT knowledge favored digital CBT as well.

The results of this trial provide support for the utility of digital CBT in conjunction with brief weekly monitoring for individuals with AUD. Participants assigned to digital CBT showed substantial increases in rates of abstinence from alcohol during a relatively brief treatment period, from an average of 47.6% days abstinent in the month prior to enrollment to 75.1% in the final month of treatment. This represents a greater than 50% improvement in the number of nondrinking days, exceeding benchmarks identified as clinically significant improvement.^42^ Moreover, these participants continued to increase their frequency of nondrinking days through a 6-month period after access to digital CBT ended (82.6% days abstinent in final follow-up month).

What was most striking was that digital CBT plus brief monitoring consistently outperformed clinician-delivered CBT in terms of increasing alcohol abstinence, as well as the frequency of cognitive and behavioral coping strategies. Although this trial was not powered to directly test differences in effects on drinking between digital CBT and clinician-delivered CBT, post hoc analyses showed significant differences between the 2 forms of CBT delivery when directly compared over the entire 8-month study period.

Findings from this study should not be used to broadly conclude that digital CBT is superior to clinician-delivered CBT. Among the reasons why participants assigned to digital CBT demonstrated better outcomes than clinician-delivered CBT may be the differential influence of the COVID-19 pandemic on the delivery of in-person interventions in this trial. The format, duration of sessions, and fidelity monitoring substantially changed during the pandemic, likely impacting the dose and quality of CBT for 6 of the 32 participants (18.8%). The format, duration, and content of the digital CBT program was unchanged in the context of the COVID-19 pandemic. Another possible explanation is that weekly CBT with homework monitoring may have been less flexible for some in this outpatient population. Although differences in the rates of treatment completion were not statistically significant, those assigned to CBT had the lowest rates of completion, with only 50.0% attending at least 5 sessions. Future work is needed to explore patient characteristics associated with treatment completion across digital or clinician-delivered CBT.

The limited differences in outcomes between either CBT treatment and TAU may be because standard care in many addiction treatment facilities includes skills-focused group therapy or other evidence-based treatments. Most facilities require training in evidence-based treatments and clinicians often report using CBT in their daily practice.^6^ In some ways, CBT has become the standard of care at most facilities. A meta-analysis showed no significant difference in substance use outcomes when CBT was compared with another evidence-based treatment.^2^

### Strengths and Limitations

Strengths of this study include the rigorous methodologic features, such as urn randomization, a well-characterized sample, fidelity monitoring of intervention delivery, and high rates of follow-up data collection. Also, the sample is demographically diverse. Rates of heavy drinking and AUDIT scores at baseline in this clinical sample were comparable with other trials with outpatient treatment seekers.^43,44^ However, the findings are limited by a small sample size that was below target based on power estimations, largely due to the influence of COVID-19 on enrollment. Also, despite differential treatment effects on self-reported alcohol use, rates of negative EtG urine test results did not differ significantly across conditions. However, urine EtG testing has limitations as an outcome in clinical trials.^45^ In addition, the length of study treatment was relatively brief compared with a standard CBT course of treatment.

## Conclusion

This RCT provides support for the efficacy of digital CBT at increasing alcohol abstinence when provided with brief clinical support. The acceptance of digital therapeutics as a tool to address mental health and substance use issues has grown in the past decade, particularly during the COVID-19 era,^46,47^ yet many of the products marketed to address substance use lack research validation.^48^ While more work is needed to identify the optimal strategies for implementation in routine clinical practice, as well as patient characteristics that may moderate outcomes, these findings contribute further evidence that this digital CBT program provided with weekly clinical monitoring is an effective and appealing option for patients seeking help for AUD.

## Notes

Supplement 1.
Trial Protocol

Supplement 2. **eTable.** Percentage of Days Abstinent from Alcohol (PDA) and Percentage of Heavy Drinking Days (PHDD) at Each Study Time Point

Supplement 3.
Data Sharing Statement
