# Supplementary material for: A Digital Cognitive Behavioral Therapy Program for Adults With Alcohol Use Disorder: A Randomized Clinical Trial
Source: JAMA Netw Open. 2024 Sep 26;7(9):e2435205. doi: 10.1001/jamanetworkopen.2024.35205 (PMC11428014; doi:10.1001/jamanetworkopen.2024.35205)
Supplement: Supplement 1. — Trial Protocol [file jamanetwopen-e2435205-s001.pdf]

HIC# 1511016759

YALE UNIVERSITY
HUMAN INVESTIGATION COMMITTEE

Application to Involve Human Subjects in Biomedical Research
100 FR1 (2011-6)

Please refer to the HIC website for application instructions and information required to complete this application. The Instructions are available at http://www.yale.edu/hrpp/forms-templates/biomedical.html Submit the original application and two (2) copies of all materials including relevant sections of the grant which funds this project (if applicable) to the HIC.	**HIC OFFICE USE ONLY**	

SECTION I: ADMINISTRATIVE INFORMATION

**Title of Research Project:** : Efficacy and Mechanisms of CBT4CBT for Alcohol Use Disorders			
**Principal Investigator:** Brian Kiluk, PhD		**Yale Academic Appointment:** Assistant Professor	
**Campus Address:** 40 Temple Street, Suite 6C; New Haven, CT 06511			
**Campus Phone:** 203-737-3314	**Fax:** 203-737-1371	**Pager:**	**E-mail:** brian.kiluk@yale.edu
**Protocol Correspondent Name & Address** (*if different than PI*): Joanne Corvino; 40 Temple Street, Suite 6C; New Haven, CT 06511			
**Campus Phone:** 203-785-2012	**Fax:** 203-737-1371	**E-mail:** joanne.corvino@yale.edu	
**Yale Cancer Center CTO Protocol Correspondent Name & Address** (*if applicable*): **N/A**			
**Campus Phone:**	**Fax:**	**E-mail:**	

**Faculty Advisor:**(required if PI is a student, resident, fellow or other trainee) ☒ **NA**		**Yale Academic Appointment:**	
**Campus Address:**			
**Campus Phone:**	**Fax:**	**Pager:**	**E-mail:**

Investigator Interests:

APPROVED BY THE YALE UNIVERSITY IRB 2/15/2021

Does the principal investigator, co-investigator, or any other responsible research team member, or any of their family members (spouse, child, domestic partner) have an incentive or interest, financial or otherwise, that may be viewed as affecting the protection of the human subjects involved in this project, the scientific objectivity of the research or its integrity? See Disclosures and Management of Personal Interests in Human Research http://www.yale.edu/hrpp/policies/index.html#COI

☐ Yes √ No

If yes, list names of the investigator or responsible person:

The Yale University Principal Investigator and all Yale University and Yale New Haven Hospital individuals who are listed as co-investigators on a protocol with a Yale University Principal Investigator must have a current financial disclosure form on file with the University's Conflict of Interest Office. If this has not been done, the individual(s) should follow this link to the COI Office Website to complete the form: http://www.yale.edu/coi/

NOTE: The requirement for maintaining a current disclosure form on file with the University's Conflict of Interest Office extends primarily to Yale University and Yale-New Haven Hospital personnel. **Whether or not they are required to maintain a disclosure form with the University's Conflict of Interest Office, all investigators and individuals deemed otherwise responsible by the PI who are listed on the protocol are required to disclose to the PI any interests that are specific to this protocol.**

SECTION II: GENERAL INFORMATION

1. **Performing Organizations:** Identify the hospital, in-patient or outpatient facility, school or other agency that will serve as the location of the research. Choose all that apply:

a. Internal Location[s] of the Study:

☐ Magnetic Resonance Research Center (MR-TAC)
☐ Yale Cancer Center/Clinical Trials Office (CTO)
☐ Yale Cancer Center/Smilow
☐ Yale-New Haven Hospital
☐ Specify Other Yale Location:
☐ Yale University PET Center
☐ YCCI/Church Street Research Unit (CSRU)
☐ YCCI/Hospital Research Unit (HRU)
☐ YCCI/Keck Laboratories
☐ Cancer Data Repository/Tumor Registry

b. External Location[s]:

☐ APT Foundation, Inc.
☐ Connecticut Mental Health Center
☐ Clinical Neuroscience Research Unit (CNRU)
☒ Other Locations, Specify:
MCCA (Specify location(s))
Bridgeport, CT and New Haven, CT
AND
SATU
1 Long Wharf Drive; New Haven, CT
☐ Haskins Laboratories
☐ John B. Pierce Laboratory, Inc.
☐ Veterans Affairs Hospital, West Haven
☐ International Research Site

APPROVED BY THE YALE UNIVERSITY IRB 2/15/2021

c. Additional Required Documents ***(check all that apply)*****:** ☒ N/A

☐ *YCCI-Scientific and Safety Committee (YCCI-SSC) Approval Date:
☐ *Pediatric Protocol Review Committee (PPRC) Approval Date:
☐ *YCC Protocol Review Committee (YRC-PRC) Approval Date:
☐ *Dept. of Veterans Affairs, West Haven VA HSS Approval Date:
☐ *Radioactive Drug Research Committee (RDRC) Approval Date:
☐ YNHH-Radiation Safety Committee (YNHH-RSC) Approval Date:
☐ Magnetic Resonance Research Center PRC (MRRC-PRC) Approval Date:
☐ YSM/YNHH Cancer Data Repository (CaDR) Approval Date:
☐ Dept. of Lab Medicine request for services or specimens form

Approval from these committees is required before final HIC approval is granted. See instructions for documents required for initial submission and approval of the protocol. Allow sufficient time for these requests. Check with the oversight body for their time requirements.

2. **Probable Duration of Project:** State the expected duration of the project, including all follow-up and data analysis activities.
   April 2016-July 2021
3. **Research Type/Phase: (Check all that apply)**
   a. **Study Type**
   ☒ Single Center Study
   ☐ Multi-Center Study
   Does the Yale PI serve as the PI of the multi-site study? Yes ☐ No ☐
   ☐ Coordinating Center/Data Management
   ☐ Other:

   b. **Study Phase** ☐ **N/A**
   ☐ Pilot ☐ Phase I ☒ Phase II ☐ Phase III ☐ Phase IV
   ☐ Other (*Specify)*

4. **Area of Research: (Check all that apply)** Note that these are overlapping definitions and more than one category may apply to your research protocol. Definitions for the following can be found in the instructions section 4c:

☒ Clinical Research: Patient-Oriented
☐ Clinical Research: Epidemiologic and Behavioral
☐ Translational Research #1 ("Bench-to-Bedside")
☐ Translational Research #2 ("Bedside-to-Community")
☒ Clinical Research: Outcomes and Health Services
☐ Interdisciplinary Research
☐ Community-Based Research

5. Is this study a clinical trial? Yes ☒ No ☐

APPROVED BY THE YALE UNIVERSITY IRB 2/15/2021

NOTE the current ICMJE (International Committee of Medical Journal Editors) definition of a clinical trial: "any research study that prospectively assigns human participants or groups of humans to one or more health-related interventions to evaluate the effects on health outcomes." Health-related interventions include any intervention used to modify a biomedical or health-related outcome (for example, drugs, surgical procedures, devices, behavioral treatments, dietary interventions, and process-of-care changes). Health outcomes include any biomedical or health-related measures obtained in patients or participants, including pharmacokinetic measures and adverse events"

If yes, where is it registered?

Clinical Trials.gov registry ☒

Other (*Specify*)

*Registration of clinical trials **at their initiation** is required by the FDA, NIH and by the ICMJE.*

If this study is registered on clinicaltrials.gov, there is new language in the consent form and compound authorization that should be used.

For more information on registering clinical trials, including whether your trial must be registered, see the YCCI webpage, http://ycci.yale.edu/researchers/ors/registerstudy.aspx or contact YCCI at 203.785.3482)

6. Will this study have a billable service as defined by the Billable Service Definition?

Yes ☐ No☒

If you answered "yes", this study will need to be set up in Patient Protocol Manager (PPM) http://medicine.yale.edu/ymg/systems/ppm/index.aspx

7. Are there any procedures involved in this protocol that will be performed at YNHH or one of its affiliated entities? Yes ___ No **X** *If Yes, please answer questions a through c and note instructions below. If No, proceed to Section III.*

a. Does your YNHH privilege delineation currently include the **specific procedure** that you will perform?

b. Will you be using any new equipment or equipment that you have not used in the past for this procedure?

c. Will a novel approach using existing equipment be applied?

If you answered "no" to question 7a, or "yes" to question 7b or c, please contact the YNHH Department of Physician Services (688-2615) for prior approval before commencing with your research protocol.

SECTION III: FUNDING, RESEARCH TEAM AND TRAINING

1. **Funding Source:** Indicate all of the funding source(s) for this study. Check all boxes that apply. Provide information regarding the external funding source. This information should include identification of the agency/sponsor, the funding mechanism (grant or contract), and whether the award is pending or has been awarded. Provide the M/C# and Agency name (if grant-

funded). If the funding source associated with a protocol is "pending" at the time of the protocol submission to the HIC (as is the case for most NIH submissions), the PI should note "Pending" in the appropriate section of the protocol application, provide the M/C# and Agency name (if grant-funded) and further note that University (departmental) funds support the research (until such time that an award is made).

PI	Title of Grant	Name of Funding Source	Funding	Funding Mechanism
Brian Kiluk, PhD	Efficacy and Mechanisms of CBT4CBT for Alcohol Use Disorders	NIAAA	☒ Federal<br>☐ State<br>☐ Non Profit<br>☐ Industry<br>☐ Other For Profit<br>☐ Other	☐Grant-M# R01AA024122-01A1<br>☐Contract#<br>☐Contract Pending<br>☐ Investigator/Department Initiated<br>☐ Sponsor Initiated<br>☐ Other, Specify:

IRB Review fees are charged for projects funded by Industry or Other For-Profit Sponsors. Provide the Name and Address of the Sponsor Representative to whom the invoice should be sent. ***Note: the PI's home department will be billed if this information is not provided.***

Send IRB Review Fee Invoice To: **N/A**
Name:
Company:
Address:

2. **Research Team:** List all members of the research team. Indicate under the affiliation column whether the investigators or study personnel are part of the Yale faculty or staff, or part of the faculty or staff from a collaborating institution, or are not formally affiliated with any institution. **ALL members of the research team MUST complete Human Subject Protection Training (HSPT) and Health Insurance Portability and Accountability Act (HIPAA) Training before they may be listed on the protocol. See NOTE below.**

	Name	Affiliation: Yale/Other Institution (Identify)	NetID
**Principal Investigator**	Brian Kiluk, PhD	Yale	BK84
**Role: Co-Investigator**	Elise DeVito, PhD	Yale	EED32
**Role: Co-Investigator**	Stephanie O'Malley, PhD	Yale	SS02
**Role: Data Manager**	Tami Frankforter, MS	Yale	TLF7
**Role: Research Assistant**	Lawanda Frederick	Yale	LF384
**Role: Administrator**	Joanne Corvino	Yale	JKC9
**Role: Research Assistant**	Elizabeth Doohan	Yale	ED266

NOTE: The HIC will remove from the protocol any personnel who have not completed required training. A personnel protocol amendment will need to be submitted when training is completed.

SECTION IV: PRINCIPAL INVESTIGATOR/FACULTY ADVISOR/ DEPARTMENT CHAIR AGREEMENT

As the **principal investigator** of this research project, I certify that:

- The information provided in this application is complete and accurate.
- I assume full responsibility for the protection of human subjects and the proper conduct of the research.
- Subject safety will be of paramount concern, and every effort will be made to protect subjects' rights and welfare.
- The research will be performed according to ethical principles and in compliance with all federal, state and local laws, as well as institutional regulations and policies regarding the protection of human subjects.
- All members of the research team will be kept apprised of research goals.
- I will obtain approval for this research study and any subsequent revisions prior to my initiating the study or any change and I will obtain continuing approval of this study prior to the expiration date of any approval period.
- I will report to the HIC any serious injuries and/or other unanticipated problems involving risk to participants.
- I am in compliance with the requirements set by the University and qualify to serve as the principal investigator of this project or have acquired the appropriate approval from the Dean's Office or Office of the Provost, or the Human Subject Protection Administrator at Yale-New Haven Hospital, or have a faculty advisor.
- I will identify a qualified successor should I cease my role as principal investigator and facilitate a smooth transfer of investigator responsibilities.

______________________________ ____________________

PI Name (PRINT) and Signature Date

As the **faculty advisor** of this research project, I certify that:

- The information provided in this application is complete and accurate.
- This project has scientific value and merit and that the student or trainee investigator has the necessary resources to complete the project and achieve the aims.
- I will train the student investigator in matters of appropriate research compliance, protection of human subjects and proper conduct of research.
- The research will be performed according to ethical principles and in compliance with all federal, state and local laws, as well as institutional regulations and policies regarding the protection of human subjects.
- The student investigator will obtain approval for this research study and any subsequent revisions Prior to initiating the study or revision and will obtain continuing approval prior to the expiration of any approval period.
- The student investigator will report to the HIC any serious injuries and/or other unanticipated problems involving risk to participants.
- I am in compliance with the requirements set forth by the University and qualify to serve as the faculty advisor of this project.

Department Chair's Assurance Statement

Do you know of any real or apparent institutional conflict of interest (e.g., Yale ownership of a sponsoring company, patents, licensure) associated with this research project?
☐ Yes (provide a description of that interest in a separate letter addressed to the HIC.)
☐ No

As Chair, do you have any real or apparent protocol-specific conflict of interest between yourself and the sponsor of the research project, or its competitor or any interest in any intervention and/or method tested in the project that might compromise this research project?
☐ Yes (provide a description of that interest in a separate letter addressed to the HIC)
☐ No

I assure the HIC that the principal investigator and all members of the research team are qualified by education, training, licensure and/or experience to assume participation in the conduct of this research trial. I also assure that the principal investigator has departmental support and sufficient resources to conduct this trial appropriately.

____   ____________
Chair Name (PRINT) and Signature   Date

____
Department

YNHH Human Subjects Protection Administrator Assurance Statement

Required when the study is conducted solely at YNHH by YNHH health care providers.

As Human Subject Protection Administrator (HSPA) for YNHH, I certify that:

- I have read a copy of the protocol and approve it being conducted at YNHH.
- I agree to notify the IRB if I am aware of any real or apparent institutional conflict of interest.
- The principal investigator of this study is qualified to serve as P.I. and has the support of the hospital for this research project.

____   __________________
YNHH HSPA Name (PRINT) and Signature   Date

APPROVED BY THE YALE UNIVERSITY IRB 2/15/2021

For HIC Use Only	
Date Approved	Human Investigation Committee Signature
This protocol is valid through ______	

SECTION V: RESEARCH PLAN

1. **Statement of Purpose:** State the scientific aim(s) of the study, or the hypotheses to be tested.

Well-controlled randomized clinical trials of computer-assisted therapies are very rare, and there are few that would meet current methodological standards established for traditional evidence-based treatments. We propose to conduct a randomized clinical trial of our web-based version of Computer Based Training in Cognitive Behavioral Therapy (CBT4CBT) to evaluate its effectiveness relative to therapist-delivered cognitive behavioral therapy or standard outpatient counseling with participants with alcohol use disorder. Specific aims are as follows:
Aim 1 – ***Evaluate the efficacy of CBT4CBT and clinician-delivered CBT relative to standard treatment for reducing alcohol use through an 8-week randomized trial with 6-month follow-up.*** Our primary hypothesis is that either form of CBT will be more effective than standard treatment at increasing the percentage of days abstinent (PDA)[2, 3] during treatment (8 weeks) and through the follow-up (6 months), assessed via Timeline FollowBack interviews[4, 5]. Secondary outcomes will include percentage of heavy drinking days (PHDD)[6, 7], urine ethyl glucoronide analysis (EtG)[8-10], drinking-related consequences[11, 12], quality of life[13], and cost effectiveness[14].

Aim 2 – ***Evaluate the extent to which CBT's putative mechanisms act as mediator of treatment effect on reducing alcohol use in both forms of CBT relative to standard treatment.*** Hypothesized mechanisms include enhanced behavioral, cognitive, and affective control, measured within treatment by indices reflecting acquisition and implementation of targeted skills (behavioral role-play assessment[15], coping strategies inventory[16, 17], and measures of homework completion and knowledge of CBT). Latent growth curve modeling will test for mediation using the product of coefficients method[18, 19]. We hypothesize:
(a) greater increases in skills acquisition for those assigned to either form of CBT compared to TAU;
(b) increased acquisition of skills during treatment will be associated with greater PDA during follow-up;
(c) CBT's effect (in either delivery format) on PDA will be mediated by skills acquisition.

Exploratory Aim -- ***To determine if genetic risk moderates the response to treatment***. We hypothesize that genetic risk related to substance use disorders, co-occurring mental health disorders and cognitive dysfunction will negatively affect treatment response.

APPROVED BY THE YALE UNIVERSITY IRB 2/15/2021

The primary outcome measures will be reduction in alcohol use (percent days abstinent - PDA) assessed via timeline follow back interviews. Secondary outcomes will include the percentage of heavy drinking days (PHDDs), urine ethyl glucorinide analysis (EtG), drinking-related consequences, quality of life, and cost-effectiveness. Analyses will also determine whether web-based CBT4CBT retains key characteristics of traditional clinician-delivered CBT (e.g., acquisition of coping skills, use of change strategies) and to explore these as potential mediators of outcome (mechanisms of action). Measures of cognitive function (e.g., sustained attention, working memory, response inhibition, planning, cognitive flexibility, decision-making) will be collected to explore the moderating effect on treatment outcomes.

2. **Background:** Describe the background information that led to the plan for this project. Provide references to support the expectation of obtaining useful scientific data.

A SIGNIFICANCE

Cognitive behavioral therapy (CBT) for alcohol use disorders has considerable empirical support for its efficacy[21-25], and has shown to be highly compatible with pharmacotherapies for alcohol dependence[26-33]. This is consistent with evidence supporting CBT's efficacy across a number of psychiatric disorders, including depression, anxiety disorders, and eating disorders[24, 34-36]. Moreover, CBT stands out as a comparatively durable approach among empirically validated treatments for alcohol use disorders[37, 38].

A1. Obstacles to broad dissemination and effectiveness of CBT

Despite CBT's strong support from efficacy trials, it has proven challenging to disseminate to the clinical community[39-43]. Although clinicians and program directors report that they frequently utilize a range of empirically validated treatments in standard practice[40], there is little evidence that they are actually doing so, particularly in a manner likely to improve patient outcomes[44, 45]. Despite clinicians frequently reporting use of CBT in clinical practice[46-48], objective evidence suggests this is not the case. For example, independent ratings of over 300 audiotapes of clinicians conducting standard individual counseling as practiced in 11 clinics across the US indicated that CBT interventions were virtually undetectable; any type or level of coping skill training was observed in only 3% of sessions and any mention of cognitions in only 8% of sessions[49]. Furthermore, data from randomized clinician training studies[50-52] highlight the multiple challenges in transferring empirically validated treatments to clinical practice. For example, similar to Motivational Interviewing training trials[52], we found in-person training plus ongoing supervision is the only training method that results in clinician competence at levels that would be commensurate with those in CBT efficacy trials[50]. This level of training, however effective, is too costly and time-intensive to be feasible to train large numbers of clinicians in complex approaches like CBT[45], particularly given the limited resources of most alcohol/drug use treatment settings[40, 53, 54].

A2. Limited understanding of CBT mechanisms; methodological challenges

In addition to the challenges of dissemination and implementation of CBT in clinical practice, the lack of understanding regarding CBT's mechanisms also limits the extent of effectiveness for the broad population of individuals with alcohol use disorders. *In order to translate CBT's effects from research to practice, we need better understanding of mechanism, including optimal conditions for delivery and what components must not be diluted to achieve change*[55, 56]. **Although there is strong evidence that CBT works to reduce alcohol use, answers to the**

questions of how, and for whom it works are not resolved[57, 58]. CBT is based on the premise that individuals with alcohol problems develop impaired affective, behavioral and cognitive control over stressful states and opportunities to drink and hence lower self-efficacy[59-62]. Thus, one promising explanation for CBT's effectiveness and durability is its focus on conveying *generalizable skills* to exert cognitive/behavioral control over over-learned behavior and patterns, reduce impulsive responding to seek immediate reward in response to alcohol cues via control of craving strategies, improve decision-making and problem-solving skills, and recognize, challenge, and exert control over cognitions associated with alcohol use[63].

Strategies for evaluating an intervention's mechanisms of behavior change are comparatively well established[55, 64-67]. Morgenstern and Longabaugh's[68] causal chain to demonstrate skill acquisition as a mechanism of CBT proposed four criteria, which included evidence that inclusion of coping behaviors as a covariate reduced the effect of CBT on drinking outcome (statistical mediation[69]). **However, much of the evidence regarding skills acquisition from CBT trials for alcohol use fails to meet all four of these criteria[16, 68, 70, 71].** Several methodological features likely contribute to the mixed evidence regarding CBT's mechanisms: First, most trials evaluating clinician-delivered interventions face issues of variability in treatment delivery that may confound the evaluation of mechanisms. This includes variability in fidelity to the intervention[72-74], dose and quality of delivery[75], as well as '*therapist effects' that may directly affect the active treatment ingredients hypothesized to be responsible for contributing to behavior change*. Clinicians delivering standardized treatment in clinical trials regularly show substantial differences in the outcomes of clients they treat[76-78], and in some trials, 'therapist effects' account for more variability in outcome than treatment factors[79-81]. Furthermore, distinguishing non-specific factors from specific active ingredients has proven difficult in trials examining mediators across clinician-delivered treatments[58, 82, 83]. **Specific effects are inseparable from 'relational' effects (e.g., therapeutic alliance) when treatments are delivered in the context of an interpersonal relationship[76].** Second, there are relatively few reliable measures of skills acquisition and implementation[16, 59, 68], as many self-reported assessments may not be adequately sensitive to the specific skills taught nor measured at the appropriate intervals to demonstrate change prior to the outcome[55, 65]. Third, given the complexity of behavior change, trials have been limited by inclusion of only one level of analysis of the mechanism, rather than considering the contributions of multiple processes to form a more complete understanding of how, and for whom, CBT works. For instance, there is considerable evidence that chronic, heavy alcohol use affects cognitive abilities associated with controlled and effortful processing of novel information, including executive functions and fluid cognitive abilities[84-89], which have been shown to affect treatment processes[90-93], and may affect the delivery of a cognitively-demanding therapy such as CBT (e.g., moderation of a mediator). Lastly, traditional methods for demonstrating statistical mediation (e.g., causal steps approach) have been limited by testing single mechanisms with inadequate power for detecting mediated effects[94, 95]. More advanced statistical approaches have indicated the improvement in coping skills may mediate the effect of CBT on reductions in primary drug use[96], marijuana[97], gambling[98], and chronic pain[99].

A3. Potential of computer-delivered interventions to address obstacles

The emergence of computer-delivered interventions offers tremendous promise with respect to making evidence-based treatments more broadly accessible to those who may benefit from

APPROVED BY THE YALE UNIVERSITY IRB 2/15/2021

them[100, 101]. This is crucial given that the vast majority of those with alcohol use disorders do not receive treatment[102-104]. Computer-delivered interventions have the potential to reach rural populations and other groups whose access to treatment is limited, as well as those who do not seek treatment because of discrimination or stigma[105, 106]. Furthermore, they also offer significant advantages of standardization and consistent quality, reduction of cost and clinician time, and potential 24/7 availability[14, 107-111]. **In addition, computer-delivered interventions may also facilitate evaluation of mechanisms via delivery of active treatment ingredients in a more focused and concentrated form than is possible with clinician-delivered treatments.** One of the key benefits of computerized delivery of interventions is standardization[101, 112, 113] relative to traditional clinician-delivered treatments with respect to variability in treatment fidelity, quality, and delivery of active and common elements[114]. These characteristics make computer-delivered interventions a promising solution to the dissemination and implementation challenges, as well as the challenges in identifying mechanisms, that have limited CBT's broad use in clinical practice.

However, in order for this potential to be realized, computer-delivered interventions need to be evaluated in well-controlled high-quality randomized trials, with **appropriate control and comparison conditions**. Computer-delivered interventions should be subject to the same rigorous testing and methods that are required for establishing efficacy of behavioral and pharmacologic interventions prior to their dissemination[115, 116]. *Ideally such trials would include clinician- and computer-delivered versions of the same intervention, as adoption of computerized versions of empirically supported treatments is predicated upon their retaining the efficacy and putative active ingredients of the original clinician-delivered approach. Further, such a design would also allow for investigation of client characteristics that influence how mechanisms operate in each form of treatment delivery, which would have high clinical utility.*

The current state of research on computer-delivered interventions includes very few trials with such a design. Our recent methodological review of the existing randomized clinical trials evaluating all computer-delivered interventions for adult Axis I disorders[117] found that only **3 of the 75 trials** evaluated met minimal methodology standards for evaluating intervention efficacy, with most utilizing weak wait-list control conditions, non-validated self-reports of outcome, and having poor rates of follow-up. Regarding computer-delivered interventions specifically for alcohol use, a 2011 meta-analyses of 25 randomized trials concluded "At present, it is not possible to interpret the evidence with any degree of certainty. **The current literature is limited by small sample sizes, short-term follow-up, few studies in non-student adult populations and few comparisons with active comparator groups"[118].** Thus, evaluation of a computer-delivered version of CBT within a well-controlled trial that includes a clinician-delivered version would fill an important gap and potentially lead to a valuable new and easily disseminated treatment resource.

A4. Development and evaluation of computer-delivered CBT

In response to the (1) limited availability of CBT in clinical settings, (2) complexity of training in CBT and the increasing time constraints on clinicians, and (3) need to develop a version of CBT that can be implemented more consistently, with a high level of quality and at lower cost, we developed a computer-based training version of CBT for substance use disorders, called CBT4CBT[119]. The original CBT4CBT program consists of seven modules targeting drug use

APPROVED BY THE YALE UNIVERSITY IRB 2/15/2021

disorders, the content of which is based closely on the NIDA CBT manual[120]. In developing CBT4CBT, we sought to construct a highly engaging version of CBT that could take advantage of the capacity of computer-based learning to convey key CBT skills via a range of media (e.g., video, graphics, audio instruction, interactive exercises). In particular, we capitalized on the use of video-based examples to emphasize learning of targeted behavioral, cognitive, and affective strategies, with emphasis on modeling from examples of individuals utilizing skills in a range of realistic situations. We conceived of CBT4CBT as a 'skills training machine'; one that seeks to teach a core set of generalizable CBT strategies (functional analysis, coping with craving and strong affect, problem solving, decision making, challenging thoughts, and assertive drug refusal). Interactive exercises are included to reinforce patients' understanding of targeted skills, as well as practice assignments (i.e., homework) to encourage implementation and practice of skills, and thus enhance durability of treatment effects. Given our previous work linking out-of-session practice of homework with skill acquisition and outcome in CBT[121], the CBT4CBT program places extensive emphasis on practice of skills through interactive homework exercises and close monitoring of homework completion.

We have completed two randomized trials of CBT4CBT within independent samples of drug users[119, 122, 123] (PRELIMINARY DATA – C.1.a.). These have indicated: (1) statistically significant effects over standard treatment on biological outcomes (% drug-negative urine specimens)[119, 122], (2) enhanced durability through a 6-month follow-up[122, 123], (3) significant differences with respect to standard treatment in acquisition of coping skills[96], and (4) **changes in acquisition of coping skills via CBT-mediated reduction in drug use through follow-up[96, 124]**. Dr. Kiluk's 2010 report was one of the first successful demonstrations of skills acquisition as a mediator of CBT's effect on drug use (PRELIMINARY DATA – C.1.b.), meeting the criteria described by Morgenstern and Longabaugh[68], as well as most of those outlined by Kazdin and Nock[64]. Several aspects of these trials may have enhanced our ability to detect mediation effects: First, we included a situational role-play assessment[15] for measuring skill acquisition, which may have provided a more valid assessment than self-report questionnaires. Second, we focused on the **quality rather than quantity** of skills, which may have provided a more meaningful indicator of skill acquisition. Third, standardized delivery of CBT via computer may have facilitated detection of changes in acquired skills and mediating effects by reducing variability in treatment delivery.

Dr. Kiluk led the team that developed the adaptation of CBT4CBT specifically targeting alcohol use, which was completed in 2011. The program follows the format used in the original drug-based version, with content, situations, and videos adapted for individuals with alcohol use disorders and based closely on the NIAAA CBT manual[125]. We *have completed an R21 trial establishing its feasibility, safety, and preliminary efficacy at reducing alcohol use within a sample of outpatient treatment seekers (PRELIMINARY DATA – C.1.e.).* **The next logical step in this program of research is a larger randomized controlled trial, which would include traditional clinician-delivered CBT and treatment as usual (TAU) in order to evaluate whether the computerized version retains key characteristics of clinician-delivered CBT.** Although a direct comparison of CBT4CBT to clinician-delivered CBT (e.g., a non-inferiority study)[126-128] would be premature at this stage (and not sufficiently powered in the proposed project), such a design could compare the cost-effectiveness of the two formats, as well as generate effect size and power estimates for a future non-inferiority trial. ***Additionally, this***

design is an ideal platform to evaluate mechanisms of CBT, for several reasons[65, 129]. First, inclusion of a treatment condition consisting of CBT4CBT delivered with minimal clinical contact (*focused on monitoring clients' safety and assessing signs/symptoms of clinical deterioration*) would offer the evaluation of an isolated form of CBT's main active ingredient - coping skills training, without the presence of therapist factors and common factors associated with clinician-delivered CBT. Second, although both delivery formats of CBT may facilitate change through similar mechanisms (e.g., enhanced affective, behavioral, and cognitive control), the processes by which they affect these mechanisms may differ in several ways. For instance, there is likely to be a larger 'dose' of CBT skills training in the computer-delivered version. It is also possible that the interpersonal and tailored discussion of completed homework activities may facilitate out-of-session practice in clinician-delivered CBT, whereas viewing the video-based modeling of skills implementation may be most salient in CBT4CBT. Third, retaining a TAU condition is essential for both evaluating the efficacy of CBT4CBT for alcohol use disorders, as well as permitting evaluation of the specificity of CBT's mechanisms.

A5, BIOMARKER DEVELOPMENT TO FACILITATE CBT: Alcohol use disorder is a highly polygenic trait, which means that there are many common single nucleotide polymorphisms (SNPs) that affect risk within the population, perhaps thousands, with most having very small effects (e.g. OR < 1.1) {Gelernter, 2015 #648}. Information on individual differences in genetic risk can be used as a biomarker to develop more effective treatments as outlined by the NIH's Precision Medicine Initiative {Collins, 2015 #679}. Techniques exist to study the aggregate effect of risk SNPs in an individual's genome. For a given trait, the sum of risk SNPs in an individual's genome is their genetic risk score, or polygenic risk score (PRS) {Purcell, 2009 #566}. The PRS for individuals in a population can be estimated using the summary statistics from independent genomewide association studies (GWAS). An individual's PRS is the sum of non-correlated SNPs in their genome weighted by the SNP effects size (e.g. odds ratio) in the GWAS. SNPs with large effect in the GWAS will contribute more to an individual's PRS compared to SNPs with small effect. For a given disorder, the amount of genetic risk carried by an individual might influence their response to certain treatments. For example, it has been suggested that treatment resistant schizophrenics have more schizophrenia genetic risk compared treatment responsive schizophrenics {Frank, 2015 #567}. Characterizing genetic effects on cocaine use disorder treatment response is important because it could help to explain heterogeneity in treatment outcomes and establish a foundation to develop more effect treatments.

A6. SUMMARY

- CBT has strong empirical support for the treatment of alcohol use disorders, yet implementation into clinical practice has been challenging. *CBT's mechanisms are not yet fully understood, which may limit the potency of the intervention and the effectiveness for a broad population of those with alcohol use disorders.*
- CBT4CBT has demonstrated effectiveness at reducing illicit drug use and improving the acquisition of skills as compared to standard treatment, with benefits maintained through follow-up periods in two independent randomized clinical trials[119, 122, 123]. We have used this form of CBT to demonstrate that skill acquisition mediates its effect on treatment outcomes[96]. *Preliminary data from the completed R21 supports the feasibility, safety and preliminary efficacy of CBT4CBT for reducing alcohol use.*

- Computer-delivered interventions may reduce the variability in treatment delivery that may have limited trials of clinician-delivered interventions to demonstrate mechanisms of behavior change[101, 112, 130, 131].
- A randomized trial investigating the short- and long-term efficacy of CBT4CBT for alcohol use disorders would be particularly valuable at this stage of research, as it would not only facilitate evaluation of CBT4CBT's efficacy for alcohol use disorders with respect to standard treatment (TAU), but also permit some exploratory comparison with clinician-delivered CBT. Further, this strategy may prove a **particularly informative means of evaluating mechanisms via CBT delivery in different formats.**

B. INNOVATION

- The proposed project would be the first adequately powered RCT to evaluate the efficacy of a web-based version of CBT for alcohol use disorders. The evaluation of CBT4CBT (delivered with minimal clinician contact) will provide evidence of CBT4CBT's effect as a virtual 'stand alone' intervention for alcohol use, which could have broad implications for making this cost-effective form of CBT more broadly accessible.
- Evidence of an effective, empirically-supported web-based CBT intervention for alcohol use disorders could lead to a number of applications and potential research directions[106, 132], including dissemination to rural and other hard-to-reach populations, evaluation in combination with alcohol pharmacotherapies, or implemented as a standard psychosocial platform for medication trials[133, 134].
- Inclusion of a computer-delivered and clinician-delivered CBT in the same trial will allow some exploratory comparisons of these interventions in terms of their cost-effectiveness, specificity, and participant satisfaction. The proposed project will generate data that could inform design and power calculations for a future non-inferiority study.
- Inclusion of two distinct forms of delivery of CBT in the same trial will allow for examination of one of CBT's hypothesized primary mechanisms of change, acquisition of targeted skills. The provision of targeted skills can be examined with and without 'relational' factors that contribute to outcomes (e.g., therapist empathy, warmth, and therapeutic alliance).

C. APPROACH

C1. PRELIMINARY DATA

Our research group at Yale has completed a series of clinical trials evaluating clinician-delivered CBT in a range of populations, including alcohol[3, 135-137], cocaine[122, 138-141], and marijuana[142, 143] users. Of particular relevance to our ability to meet the specific aims of the proposed project, our trials have focused on evaluation of treatment integrity and active ingredients[75, 144-147], and on identifying mediators of outcome as potential mechanisms of behavior change[15, 96, 121, 148]. Moreover, we have achieved consistently high rates of completed follow-up interviews for our intention-to-treat samples (80-100%)[149, 150].

C.1.a. Validation of CBT4CBT in drug users

Two independent 8-week trials of CBT4CBT[14, 119, 122, 123] for drug use disorders have evaluated the efficacy of the program as an adjunct to standard outpatient treatment. The initial trial included 78 individuals (46% female, 58% ethnic minority, 77% unemployed) entering outpatient treatment randomized to either standard outpatient treatment (weekly individual

sessions and groups, TAU) or standard TAU with access to CBT4CBT weekly. Nearly 80% of participants were users of both alcohol and drugs in this trial. In terms of outcome, *those assigned to the CBT4CBT condition submitted a significantly lower proportion of urines that were positive for any drugs compared to TAU (34% vs. 53%, respectively; F=3.9, p<.05, d=.46). This difference represents a moderate effect size.* The second trial replicated these findings in a sample of 101 cocaine-dependent methadone-maintained individuals (60% female, 40% ethnic minority, 89% unemployed). Results indicated those assigned to CBT4CBT plus TAU, compared to TAU alone, were more likely to attain 3 or more weeks of continuous abstinence from cocaine (36% vs. 14%, *p<.01*), *which is a meaningful outcome measure associated with greater rates of long-term abstinence and fewer problems in psychosocial functioning*[149, 150]. In terms of durability of effects, both trials retained >80% of the intention-to-treat (ITT) sample at the 6-month follow up interview. Random effect regression analyses of drug use across time indicated a significant enduring benefit of CBT4CBT in both trials, such that those assigned to CBT4CBT plus TAU tended to decrease their substance use over the follow-up period compared to TAU alone[122, 123]. Thus, the 'sleeper effect' of CBT appears to be retained in its translation to computer-assisted format.

C.1.b. Coping skills acquisition as a mediator of treatment outcome in CBT4CBT

Evidence for the promise of using a computer-based CBT for evaluating mechanisms comes from Dr. Kiluk's analysis of differential acquisition of coping skills in the initial CBT4CBT trial[96]. We used a role-playing exercise we have validated in previous research (Drug Risk Response Test, DRRT)[15] and adapted to assess the specific coping skills taught in the CBT4CBT modules. Based on independent ratings (i.e., raters blind to treatment condition and time point) of pre/post-treatment/follow-up comparisons from 52 participants, there were significant group by time interactions, **indicating those assigned to CBT4CBT had a significantly greater increase in the quality of their coping responses across the entire study period (*F*=9.3, *p*=.004; Figure 1)**. Data from this trial also suggest that acquisition of skills during treatment may be a mediator of substance use outcomes (Fig 2, below). A series of regression equations demonstrated: (1) an effect of treatment condition on the quality of coping responses (*'a'= .26, se=.11, p<.05)* favoring CBT4CBT (TAU = 3.89, CBT4CBT = 4.40; *F*=4.6, (*df* 1,40), *p* <.05), and (2) a significant effect of the mediator (quality of coping responses) on outcome, controlling for treatment condition (*'b' = 9.19, se=3.24, p<.01)*. Using the product of coefficients method[18, 19] to determine if the mediated effect is significantly different from zero, calculated as $[(a \times b)/(se_a \times se_b)]$, resulted in Z=6.64, p<.05. Also, confidence limits for the mediated effect using the program PRODCLIN[151] resulted in upper and lower confidence intervals of 5.39 and 0.31, consistent with a statistically significant mediated effect. This suggests improvement in the quality of skills acquired during treatment was a mediator of post-treatment drug use. **This comparatively strong evidence supporting skills acquisition as a potential mediator of outcome in CBT4CBT is striking,**

Figure 1. Coping skills by treatment and time

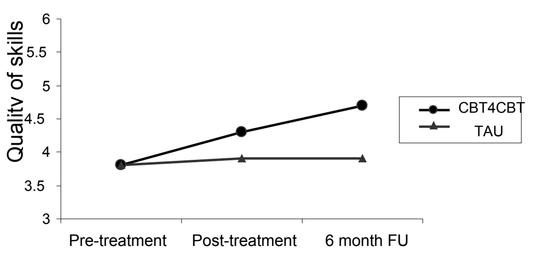

Figure 2 Quality of coping skills as mediator of CBT4CBT

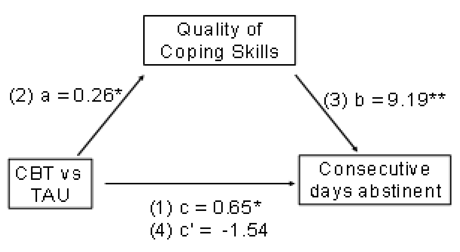

given that empirical support for mediation in CBT in the addictions has been elusive[59, 68, 124].

C.1.c. Cognitive factors associated with effectiveness of CBT4CBT

The potential role of cognitive functioning as a moderator of outcome in CBT was also suggested by Dr. Kiluk's analysis of skills acquisition and cognitive factors[152, 153]. Although examination of general intelligence measures (as estimated by Shipley Institute of Living Scale[154]) within the initial CBT4CBT trial did not indicate a direct effect on treatment retention[152], we did find an indirect effect on substance use outcomes, through the acquisition of coping skills[153]. First, results of repeated measures ANOVAs indicated significant group by time effects for the mean quality of coping response according to a median split of IQ (Group x Time, $F(1,49) = 4.31$, $p<.05$). In other words, those who scored above the median IQ improved the quality of their coping skills more than those with lower IQ, during the course of the 8 week treatment (Figure 3). Furthermore, the product of coefficients method indicated the acquisition of coping skills mediated the effect of IQ on the duration of continuous abstinence ($Z= 8.55$, $p<.05$), with confidence intervals indicative of a significant indirect effect[153]. Preliminary evidence from our most recent completed trial of CBT4CBT[122] indicated several domains of cognitive function at pre-treatment moderated treatment engagement and the acquisition of skills during treatment. For instance, greater rates of inattention on a task of sustained attention[155] at pre-treatment were associated with fewer sessions attended in TAU compared to CBT4CBT ($F(1,72)=4.69$, $p<.05$) and slower cognitive processing speed at pre-treatment was associated with lower quality of skills acquired in TAU but not CBT4CBT ($F(1,64)=5.82$, $p<.05$). These results are consistent with others that have indicated treatment processes and/or mechanisms of behavior change may be different depending on level of cognitive impairment[85, 91, 156, 157]. Thus, we have preliminary evidence that various measures of cognitive function may have moderating (and potentially indirect) effects on treatment outcome in CBT4CBT compared to standard treatment.

C.1.d. Therapeutic alliance in CBT4CBT

To explore the potential for individuals to develop some form of a therapeutic alliance with a computer-based intervention, and how it may differ from traditional features of a therapeutic alliance, we adapted a widely-used and well-validated measure of the alliance, the Working Alliance Inventory (WAI)[158], for inclusion in our most recent trial of CBT4CBT for cocaine use disorders[122]. This adapted version, the Working Alliance Inventory for Technology-based Interventions (WAI-Tech)[159], included items that measured individuals' level of relationship with the CBT4CBT program, and not their counselor or members of the research team. Overall, the WAI-Tech appeared to have similar psychometric characteristics as the standard WAI in terms of internal consistency, mean scores, and stability over time, although not surprisingly, the 'bond' subscale of the WAI-Tech was consistently lower than the other subscales and decreased over time [$F(1,52) = 5.78$, $p<.05$]. Results indicated individuals' alliance with the CBT4CBT program did not negatively affect the alliance with their counselor (as this trial included CBT4CBT in addition to TAU). However, scores on the WAI-Tech were not associated with cocaine use outcomes, whereas total scores on the standard WAI for those assigned to TAU

Figure 3. Coping Skills Over Time by IQ

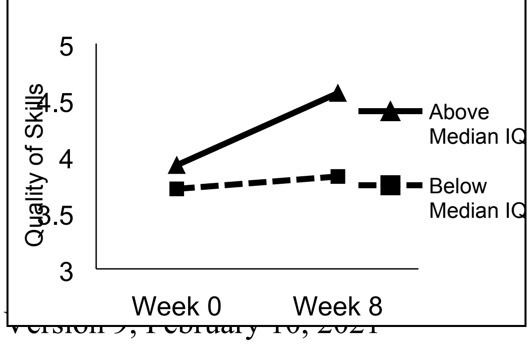

were associated with the percentage of days abstinent from cocaine ($r = .43$, $p<.05$)[159]. These findings suggest some concepts of the working (i.e., 'therapeutic') alliance may apply to computer-delivered CBT, yet the function of the alliance may be different than in face-to-face psychotherapies. **Differences in the role of the alliance on outcomes from a 'stand alone' CBT4CBT versus a clinician-delivered CBT remains an empirical question.**

C.1.e. Feasibility and preliminary support for CBT4CBT for alcohol use disorders

As mentioned above, CBT4CBT for alcohol use disorders was evaluated in an R21 pilot trial wherein 68 participants with a current alcohol use disorder were randomized to one of the following 8-week treatment conditions: (1) standard treatment as usual (TAU), (2) TAU plus CBT4CBT, or (3) CBT4CBT plus minimal clinical monitoring (i.e., 'CBT4CBT only'). *The R21 data demonstrate this version of CBT4CBT was safe, feasible, and effective at reducing alcohol use when provided within a general community outpatient facility. Of the 7 modules contained in the CBT4CBT program, participants completed an average of 5 (sd=2.2), which is comparable to our prior trials of CBT4CBT[119, 122]. Participants reported high levels of satisfaction with CBT4CBT compared to TAU (72 vs. 67% "very satisfied", respectively). Participants assigned to either CBT4CBT condition were significantly more likely to complete the 8-week course of treatment than those assigned to TAU (% completing treatment: TAU = 23%; TAU+CBT4CBT = 59%; CBT4CBT only = 63%; $X^2 = 8.72$, $p<.01$). In terms of alcohol use outcomes, there was a significant treatment condition x time effect indicating a greater increase in the percentage of days abstinent for those assigned to either CBT4CBT condition compared to TAU over the course of the 8-week trial ($F=5.81$, $p<.01$). Also, the percentage of subjects with no heavy drinking days (PSNHDD)[160] in the final month of treatment was significantly higher for the CBT4CBT conditions compared to TAU (Figure 4 - TAU = 9%; TAU+CBT4CBT = 40%; CBT4CBT only = 33.3%; $X^2 = 5.79$, $p<.05$). Furthermore, there is promising evidence those assigned to a CBT4CBT condition increased their use of change strategies[16] over time (pretreatment: M=31.9, sd=13.2; post: M=46.1, sd=15.2), increased abstinence self-efficacy[161] (pre: M=33.9, sd=13.6; post: M=35.4, sd=12.0), decreased distress intolerance[162] (pre: M=8.8, sd=8.3; post: M=7.6, sd=7.4), and increased their knowledge of CBT concepts as indicated by percent correct responses on a CBT quiz (pre: M=74.4, sd=11.8; post: M=82.4, sd=11.1). Finally, no serious adverse events (SAEs) were related to any CBT4CBT condition, indicating the relative safety of CBT4CBT provided with minimal clinician contact.*

Figure 4. PSNHDD in final month of treatment

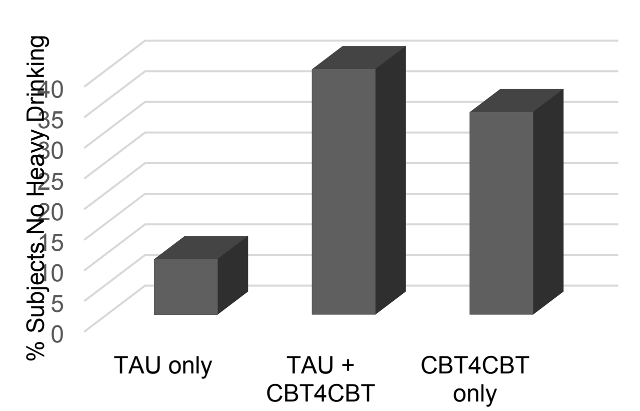

C.1.f. Summary of preliminary studies and preparation for this project

- CBT4CBT has established efficacy at reducing rates of drug use in two independent randomized trials, including the first known statistical evidence supporting skills acquisition as a mediator of CBT's effect.

- Data from our recently completed R21 evaluating the alcohol version of CBT4CBT (including CBT4CBT delivered with brief clinical monitoring) indicates that this approach is feasible, safe and well-accepted by individuals with alcohol use disorders. *Preliminary data further indicate CBT4CBT is effective at increasing the percentage of days abstinent and the percentage of subjects with no heavy drinking days compared to standard treatment only*.
- Our published and preliminary data highlight the relevant experience and developing expertise of New Investigator Dr. Kiluk (PI), with respect to evaluation of mechanisms of behavior change in computerized CBT[96, 122, 131, 153, 159, 163].

C.2. RESEARCH PLAN

C.2.a. Overview

One hundred eighty individuals seeking treatment for alcohol use at the MCCA program in Bridgeport or New Haven, CT or SATU in New Haven will be randomized to (1) standard treatment as usual (TAU) at MCCA or SATU, (2) clinician-delivered CBT, or (3) CBT4CBT for alcohol use disorders (with brief clinical monitoring). Treatments will be delivered over an 8-week period with a six-month follow-up after termination of the study treatments, consistent with prior trials of CBT4CBT[119, 122]. The primary outcome measure will be the percentage of days abstinent from alcohol (measured via Timeline FollowBack[4]), with secondary measures to include percentage of heavy drinking days, percentage of positive urine ethyl glucuronide (EtG), drinking-related consequences, quality of life, and cost effectiveness.

The primary mediator of CBT's effects will be measured by indices reflecting the acquisition and implementation of targeted skills, with additional mediators to include self-efficacy, and 'relational' mediators such as therapeutic alliance collected during the treatment period. Cognitive moderators will include experiential measures of sustained attention, working memory, response inhibition, planning, cognitive flexibility, and decision making. Participant characteristics explored as potential moderators of outcome include motivation, severity of alcohol use, and general intellectual function. The clinician-delivered CBT condition will be implemented by carefully selected MCCA or SATU master's level clinicians, highly trained and closely supervised throughout the trial. Treatment fidelity and therapist skill *in all conditions* will be evaluated by independent process raters, using methods we have worked out in previous trials[136-139, 164, 165]. *Treatment utilization and costs will be measured by the Program and Client Costs for Substance Abuse Treatment*[14, 166] *in all conditions from 12 months prior to baseline through follow up for cost-effectiveness evaluation.*

C.2.b. Participants

Participants will be 180 individuals seeking treatment at MCCA, an outpatient treatment facility, one of the largest providers of addiction treatment services within the Bridgeport-New Haven area or at SATU located in New Haven.

1. Inclusion and exclusion criteria.

Individuals will be included who:

- Are 18 years of age or older.
- Are applying for outpatient alcohol treatment and meet current DSM-5 criteria for alcohol use disorder, consuming at least 14/7 drinks (men/women) per week with at least 4 heavy drinking days reported in the past 28 days,

- Are sufficiently stable for 8 weeks of outpatient treatment and can commit to a 6-month follow-up
- Are willing to provide locator information for follow-up, and
- Are fluent in English and have a 6th grade or higher reading level.

Individuals will be excluded who:
- Have an untreated bipolar or schizophrenic disorder,
- Have a current legal case pending such that incarceration during the 8-week protocol is likely,
- *Have been prescribed an alcohol pharmacotherapy (e.g., disulfiram, naltrexone) within the past two weeks*, or
- Are physically dependent on alcohol, opioids or benzodiazepenes *such that immediate medical detoxification is necessary for safety purposes* (individuals demonstrating significant withdrawal symptoms would be eligible for re-screening following brief medical detoxification, which is arranged by MCCA or SATU staff at triage).

2. **Rationale for sample choice**. We are proposing relatively few exclusions that parallel our
completed trials of CBT4CBT[119, 123] in recruiting a broad range of individuals whose primary substance use problem is alcohol (those with low levels of drug use will be eligible providing they do not meet current disorder criteria). *The inclusion criterion regarding level of drinking is consistent with NIAAA definitions of risky/heavy drinking[167, 168] and comparable to prior randomized trials for establishing efficacy of cognitive behavioral treatments [3, 28, 169]. We will exclude those initiating an alcohol pharmacotherapy to limit potential confounds, as well as exclude individuals experiencing significant alcohol withdrawal symptoms in need of medical detoxification for safety reasons.*

C.2.c. Procedures

1. Initial screening by Research Coordinator: All new MCCA or SATU patients will be invited to participate in the protocol at the time of their initial clinic appointment. See below for detailed description.

2. Urn randomization: To increase the likelihood that treatment groups are balanced with respect to demographic variables (gender, race, education) as well as likely prognostic variables (severity of alcohol dependence, level of familiarity with and access to computers) participants will be assigned to treatment conditions through urn randomization[173, 174], using a Microsoft Access program we have implemented successfully in multiple trials [138, 142, 171, 175, 176].

3. Treatment phase: Study treatments will last 8 weeks. During the treatment phase, all participants will meet weekly virtually either via Zoom and/or phone with research staff for completion of self-report and interview assessments. In all conditions, we will closely monitor participant treatment response and safety via BAC, urine screens which are taken by the clinic on the clinic's usual schedule and assessment of psychiatric status. Although in our experience this is a very rare event in behavioral trials[177], including those of computer-delivered interventions[110, 119, 122], participants who show significant deterioration (e.g., significant increases in alcohol use or psychiatric symptoms that cannot be managed within the protocol) will be regarded as symptomatic failures, withdrawn from the treatment arm of the study, and referred for

appropriate treatment (typically a more intensive level of care). See Protection of Human Subjects, below.

4. Termination, post-treatment assessment, and follow-up: At the end of the 8-week treatment period, all participants will be re-interviewed (see Assessments) virtually either via Zoom and/or phone . Access to the CBT4CBT program will be terminated, and *participants in all three conditions will be offered the opportunity to continue in treatment as usual at MCCA or SATU if desired/needed.* Follow-up interviews (using the full post-treatment battery that includes treatment utilization) will be conducted 1, 3, and 6 months after termination virtually either via Zoom and/or phone. We will attempt to follow all participants in our intention-to-treat sample, using strategies that have been successful in our multiple previous studies[178], where our *current rate of follow-up approaches 90-95% across studies*[149, 179, 180].

5. Rationale for 8-week treatment with 6-month follow-up: We are proposing an 8-week treatment period to be consistent with prior and ongoing trials of CBT4CBT[119, 122]. The CBT4CBT program contains 7 modules; individuals are recommended to complete one module per-week (essentially mimicking a traditional once-per-week clinician-delivered CBT treatment course). *Although a 12-week (or longer) treatment period may be more typical for clinician-delivered CBT protocols, a shorter 8-week trial was chosen as the optimal time period for establishing the efficacy of CBT4CBT. Also, the NIAAA CBT manual describes only 8 core sessions, which includes a termination session (with 14 elective sessions)*[125]. Thus, an 8-week course of therapist-delivered CBT would not be unreasonable, as it would cover core topics. A 6-month follow-up period was chosen to evaluate the durability and or delayed emergence of treatment effects; CBT often shows greater effects in the period following treatment completion, as individuals continue to gain mastery of skills for avoiding alcohol and drug use (i.e., 'sleeper effect')[140]. *Moreover, there is prior evidence that individuals who attain alcohol abstinence or are low-risk drinkers (i.e., non-heavy drinkers) at 6-months post-treatment are more likely to be abstinent or low-risk drinkers at 12-months*[181, 182]*, thereby reducing the need to include a longer follow-up period and thus further reducing costs.*

6. DNA analysis

This optional part of the study will continue to be paused. Saliva samples will be collected into Oragene DNA collection kits made by DNA Genotek (Ottawa, ON, Canada). DNA will be extracted with kits made by DNA Genotek. Subjects DNA will be genotyped using the HumanOmniExpress-24 array (Illumina, San Diego, CA) or a similar high-density SNP array that has genomewide SNP content. The DNA extraction and analysis procedures will take place at the Genetics Laboratory or a Yale Core facility. Samples will be stored at the Genetics Laboratory

C.2.d. Treatments

1. Standard treatment as usual (TAU): Participants randomized to this condition will receive treatment-as-usual at MCCA or SATU. This typically consists of *group counseling delivered by masters-level and certified counselors trained and employed by MCCA or SATU and supervised by MCCA Clinic Director*, Victor Pittman, or the SATU Clinical Director, Donna La Paglia. Patients in this and all conditions are offered standard ancillary services, delivery of which is monitored closely throughout the trial (see Assessments).

2. Individual clinician-delivered CBT: Participants assigned to this condition will receive 8 weekly individual sessions of manual guided CBT[120, 125], which provides detailed instructions to clinicians regarding the same topic areas covered in CBT4CBT. Clinicians will be trained and supervised using procedures we have developed in multiple previous CBT trials[50,

[136, 138] to protect internal validity and treatment integrity. Extensive procedures for clinician selection, training, and supervision should minimize variability in outcome due to 'therapist effects'[183], however as noted earlier, some level of clinician variability is expected. The CBT clinicians will be at least masters-level clinicians drawn from MCCA or SATU staff and selected on the basis of audio-taped samples of their work. Following an initial 2-day didactic training, they will each complete at least 3 closely supervised training cases on which they will receive detailed feedback on their fidelity and skill based on ratings of their audio-recorded sessions using our validated therapy process assessment system, the Yale Adherence and Competence Scale (YACS)[75]. Once certified to begin the trial, the CBT clinicians will meet weekly with a CBT supervisor (Dr. Bold), which will involve updating of case material, review of audio recordings of each clinicians' work, and comparison of self-monitoring of CBT implementation with supervisors' ratings[145] (see Assessments). This technique, used in our previous psychotherapy studies[143, 145, 184], is included to assure fidelity to manual guidelines and a consistent high level of adherence and skill throughout the trial. Clinicians will review and make copies of completed homework assignments and will report, after each session, which CBT modules were covered.

3. CBT4CBT plus brief clinical monitoring*:* Participants assigned to this condition will be given a username and password to access the CBT4CBT website *at the clinic*. *Access to the CBT4CBT program will be restricted outside the clinic to ensure greater control over the delivery of CBT4CBT, eliminate the potential confounds of outside internet access/speed, and to reduce risks to privacy/confidentiality.* The Research Coordinator will instruct each participant concerning the first session to assure they know how to use the program and answer any questions they might have. Participants will be asked to spend at least one hour per week *working with the program at the clinic in a private room*. Participants will be asked to complete all 7 modules over the course of the 8-week study treatment period. The program tracks, for each participant, time logged onto the program, modules accessed, progress through the program from session to session, completion of homework assignments, and learning of CBT principles through multiple choice tests. Because participants will be composed of treatment-seeking individuals, participants will also meet weekly with an MCCA or SATU clinician according to clinic policy, trained and supervised *to provide brief clinical monitoring (10 minutes maximum) using our modified 'Clinical Management' manual[185], following guidelines for low-intensity interventions used in previous placebo-controlled trials[186-188] and trials of internet-delivered treatment [189]* (See Protection of Human Subjects). *These brief monitoring sessions are for the purposes of ensuring client safety and will focus on assessment of clinical symptoms and level of risk to harm self and/or others.*

Treatment Integrity: 20 audio-recorded sessions from each condition will be randomly selected and rated by trained coders blind to treatment condition, in order to evaluate therapist fidelity to manual guidelines as well as skill across conditions and therapists using the YACS[75], which includes validated scales for standard counseling, CBT, and clinical monitoring. This is included to assess treatment integrity across conditions using procedures we have worked out in multiple trials[136, 144, 147, 184, 190].

4. **Rationale for study design:** The primary aim of this trial is to provide a rigorous evaluation of

the efficacy of CBT4CBT for alcohol use disorders. *Although a 2-group design (CBT4CBT vs. TAU) may be efficient for establishing the efficacy and evaluating the mechanisms of CBT4CBT, a 3-group design including a clinician-delivered CBT provides greater scientific yield.* The standard treatment control condition allows for evaluation of relative efficacy of CBT4CBT to active outpatient alcohol treatment, *however, it would not control for expectations of improvement from CBT. The standard treatment control condition also would not permit exploration of the effects of computer- versus clinician-delivered CBT on acquisition of coping skills, duration of effects, cost effectiveness, etc. and hence we believe the inclusion of the clinician-delivered CBT condition is both novel and appropriate at this stage of evaluation of CBT4CBT.* **We recognize that this study is not powered for a direct comparison of CBT4CBT to clinician-delivered CBT (e.g., a non-inferiority analysis)**. We believe such a study would be premature, as there have been no direct comparisons of computer- versus clinician-delivered CBT for alcohol use upon which to base power calculations, and such a study would require a comparatively large sample size and would be extremely costly. Moreover, power calculations for non-inferiority analyses require estimations of confidence intervals around predetermined ranges of equivalence[126, 128, 191]. If CBT4CBT shows adequate promise in the proposed study, it could generate those estimates and sample size estimation of a possible future non-inferiority analysis. We do, however, expect a significant difference in cost effectiveness favoring CBT4CBT over clinician-delivered CBT, and can explore differences in indirect effects across conditions.

C.2.e. Assessments

To facilitate cross-study analyses as well as comply with NIH recommendations for PhenX (Consensus Measures for Phenotypes and eXposures; https://www.phenxtoolkit.org/)[192, 193], we propose a battery of assessments similar to those used in multiple previous trials through Yale's Psychotherapy Development Center, which include instruments with strong psychometric properties. Instruments used for assessing primary drinking outcomes and mediators/moderators of treatment effects are briefly described below (See Schedule of Assessments for full battery):

- **Drinking outcome:** The Timeline Follow-Back (TLFB)[4, 5] will be used for *measuring the primary outcome (PDA)* and secondary outcome percentage of heavy drinking days (PHDD), as well as measurement of frequency of tobacco product use[194] (which will include verification through measurement of exhaled CO levels[195]). A heavy drinking day is defined as any day of consumption of 5 or more standard drinks for men, and four or more standard drinks for women. *These outcomes were chosen based on CBT's focus on promoting alcohol abstinence, as well as reduction of overall alcohol consumption as a treatment goal*[125]*, and based on the evidence of PHDD as a meaningful non-abstinence based clinical indicator of treatment response*[6, 7, 196, 197]. The TLFB method for collection of drinking data will allow for the computation of a variety of drinking measures[4, 7, 198]. Urine toxicology screens for drugs of abuse will include Ethyl glucuronide (EtG), which will be used as a biological indicator of recent heavy drinking (cutoff 500 ng/ml)[10, 199-202], with the percentage of positive EtG urine results included as a secondary outcome.
- **Acquisition of targeted skills:** The acquisition and implementation of targeted skills will be assessed by: (1) the Alcohol Risk Response Test (ARRT) - a version of the Drug Risk Response Test (DRRT)[15] adapted from the Situational Competency Test[203], with excellent psychometric qualities, including high inter-rater reliability, concurrent and predictive validity[15], and treatment-specific acquisition of skills[96]; (2) Coping Strategies Inventory

(CSI)[16] - self-reported frequency of strategies for avoiding alcohol use, which has demonstrated treatment-specific coping strategies in CBT4CBT[17]; (3) the number and quality of homework assignments completed during treatment; and (4) a CBT knowledge assessment[50] - a multiple choice test that evaluates the extent to which participants understand CBT concepts, with each item drawn from the NIAAA CBT manual[125] and the content of CBT4CBT.

- **Therapeutic Alliance:** The Working Alliance Inventory (WAI)[158] will be used for measuring level of therapeutic alliance at multiple time points during the treatment period. It is a widely-used instrument designed to measure alliance between client and clinician across three dimensions (Task, Bond, Goal), and has strong psychometric properties, including association with alcohol treatment outcome[204, 205]. Both the client (WAI-C) and therapist (WAI-T) versions will be administered to all participants/clinicians across all treatment conditions. Participants assigned to the CBT4CBT condition will also complete an adapted version designed to assess their level of alliance with the computer program (WAI-Tech), which has been described in a recent report by Dr. Kiluk[159].
- **Cognitive Function: Shipley Institute of Living Scale**

Shipley Institute of Living Scale Shipley Institute of Living Scale **Schedule of Assessments**

**Instrument name**	Domain assessed	Screen/ Pre Tx	Weekly	Monthly	Post Tx (8 wks)	FU 1, 3, 6 mo
Informed Consent Quiz[216, 217]	Informed consent	X				
Screening & Demographics	Description	X				
DSM-5 diagnosis SCID [218]	Description, diagnosis	X			X	X
Brief Symptom Inventory (BSI) [219]	Moderator, monitoring	X	X		X	X
Obsessive Compulsive Drinking Scale (OCDS)[220]	Severity, monitoring	X	X		X	X
Timeline follow-back [4, 5] w/ BAC	Primary Outcome	X	X		X	X
Urine toxicology screen including ETG[199-201, 221] taken on clinic schedule	Outcome	X	X		X	X
Optional Genetics	Exploratory	X				
World Health Organization Quality of Life– Brief[222]	Quality of life, Outcome	X			X	X
Short Inventory of Problems (SIP)[12]	Consequences, Outcome	X			X	X
Alcohol Use Dis. Ident. Test (AUDIT) [223-227]	Description, moderator	X				
Shipley Institute of Living Scale [228, 229]	Cog moderator	X				
Stages of Change Questionnaire (SOC)[230]	Motivation, Moderator	X			X	X
Alcohol Risk Response Test (ARRT)[15]	Coping skills, Mediator	X		X	X	X
Change Strategies Inventory (CSI)[16, 98, 231]	Coping skills, Mediator	X		X	X	X
CBT Knowledge Quiz[50]	Knowledge, Mediator	X		X	X	X
Situational Confidence Questionnaire (SCQ)[232]	Self-efficacy, Mediator	X		X	X	X

Program and Client Costs (PACC-SAT)[14, 166, 233]	Tx utilization & cost	X		X	X	X
Yale Adherence & Competence Scale [75]	Process, Tx integrity		3 sessions/participant			
Working Alliance Inventory (WAI)[234-237]	Process, Tx integrity		Sessions 2, 6			
Endpoint rating form, satisfaction [238]	Evaluation				X	

C.2.f. Data Management and Analyses

The system of data management will include a combination of scannable forms (Teleforms), direct entry interface (Access), and a relational database (SQL Server) within a closed, secure network, allowing data to flow in a continuous cycle of collection, processing, error checking, and correction on a real-time basis while the study is ongoing. Outlined below is the general strategy for data analyses which will address each of our specific aims. Preparatory analyses will include evaluation of baseline equivalence of groups on demographic and prognostic variables, comparability of rates of data availability and treatment exposure across conditions[239, 240], *as well as consideration for the potential contribution of assessment reactivity*[241, 242]. Data analyses will be conducted on the intention to treat sample, and we will attempt to follow all participants regardless of their retention in the treatment arm of the trial[179, 180].

Aim 1 - Evaluation of treatment effects:

The principal strategy for assessing the efficacy of the study treatments on outcome will be random effects regression models for continuously measured *primary (e.g., percentage of days abstinent - PDA)* and secondary (e.g., percentage of heavy drinking days, quality of life, etc.) outcomes. Our research group has used these methods to evaluate main and interaction effects, with appropriate covariates (e.g., retention, compliance with treatment) in multiple previous trials[121, 138, 142, 171, 172]. *We will include treatment utilization data during the follow-up period as a covariate in analyses evaluating treatment effects through follow-up.*

Analysis of treatment effects at post-treatment and follow-up will include the following contrasts:

- ***H1a:*** *Main effect for CBT on PDA by month during treatment*: Clinician-CBT and CBT4CBT>TAU.
- ***H1b:*** *Main effect for CBT on PDA by month through follow-up:* Clinician-CBT and CBT4CBT>TAU.

Adequacy of sample size: *H1a* – Using conventional effect size estimates, we determined that our proposed sample size of 60 per condition would be sufficient for detecting a small-medium effect (d=.35) of treatment on PDA using a random effects regression model (2 groups, PDA by month, 3 data points, ICC=.6, alpha=.05, beta=.80). *H1b* – The proposed sample is sufficient to detect a medium effect (d=.50) through the follow-up period with a random effect regression model (2 groups, PDA by month, 7 data points, ICC=.6, alpha=.05, beta=.80). These effect sizes are conservative, based on estimates from completed studies of CBT4CBT[119, 122].

Cost-effectiveness analyses: Dr. Brian Yates of American University, an expert in cost analyses, particularly of behavioral therapies with substance using populations[243-250], is a member of the Scientific Advisory Board as well as a consultant to Dr. Carroll's NIDA-

supported Psychotherapy Development Center. In this capacity, he directs cost-effectiveness evaluations of Center projects, including all those related to CBT4CBT as well as the proposed study. *He was involved in the design of the Program and Client Costs for Substance Abuse Treatment (PACC-SAT)*[14, 166]*, the instrument that will be used to assess participants use of a wide variety of physical and mental health services, substance use and legal services utilization. The PACC-SAT covers the 12 months prior to randomization, the full-8 week treatment period, and the entire follow-up period* (See Schedule of Assessments). The general approach used to evaluate cost-effectiveness of the protocol interventions will be to evaluate the relationship between value of resources used in intervention implementation (*e.g., costs associated with the facility, staff, and transportation*) and nonmonetary outcomes produced by the intervention (*e.g., reduction in drinking behavior and drinking consequences, improvement in quality of life*). This will include calculating the total cost for each treatment condition, cost per participant in each condition, as well as outcomes of 'alcohol-free days', and comparing the average cost per 'alcohol-free day' gained per month across treatment conditions, *using strategies outlined in Dr. Yates's previous work*[243, 246, 247, 249, 250].

Aim 2 – Evaluation of skills acquisition as mediator of CBT effects:
Latent growth curve modeling will be the principal strategy for evaluating mediation using Mplus version 7[251]. The product of coefficients method[18, 19] will be used to determine the mediating effects of skills acquisition during the treatment period *on PDA* during follow-up. In this model (Figure 4), M_1 through M_3 are repeated measures (weeks 0, 4, 8) of the mediator 'acquisition of skills' (as measured by ARRT[15], CSI[16], homework completion and quality, and knowledge assessment[50]). Both the intercept (I_M) and slope (S_M) act as joint mediators of the effect of X (treatment condition) on Y (PDA during 6 month follow-up), allowing the examination of the average level (i.e., intercept) and average change over time (i.e., slope) of the mediator. We will be using the 6-month period following treatment as our alcohol use outcome in this model to support the temporal relation criterion[64], as well as to evaluate the delayed emergence of CBT's effect on alcohol use[139]. This model will test:

Figure 4. Latent Growth Curve Mediation Model

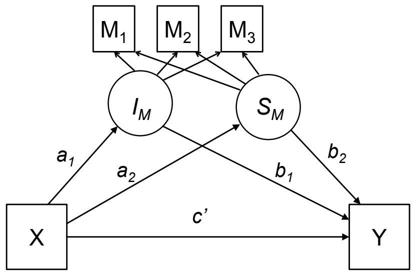

- ***H2a:*** *Effect of CBT on acquisition of skills (*a_2 path: *X→M):* Clinician-CBT>TAU; CBT4CBT>TAU
- ***H2b:*** Effect of change in acquisition *of skills on PDA by month during follow-up (*b_2 path: *M→Y)*
- ***H2c:*** *Indirect effect of CBT on PDA via change in acquisition of skills (*$a_2 * b_2 / se_{a2} * se_{b2}$*)*

Adequacy of sample size: Parameter estimates from our initial trial of CBT4CBT that supported coping skills acquisition as a mediator of treatment effects on substance use[96] were used to estimate effect sizes of treatment condition on PDA via skills acquisition (a=.26; b=.59). Based on these effect sizes and power estimates for testing mediation derived from a simulation study[94], we will have adequate power (.80) to detect significant mediating effects with the proposed sample size of 60 per condition.

Exploratory Aims:

(a) Pre-treatment cognitive function as indicated by measures from CANTAB tasks (sustained attention,
working memory, response inhibition, cognitive flexibility, planning, decision-making) will be evaluated as moderators of CBT's effect on PDA (clinician-delivered CBT and CBT4CBT vs. TAU), using linear moderation regression models, such that a significant interaction between pre-treatment cognitive function measures and treatment condition would indicate a moderated effect (i.e., the effect of CBT on PDA is dependent on levels of pre-treatment cognitive function); **(b)** Using a conditional process analysis[252], CANTAB measures at pre-treatment will be explored as a moderator of the effect of CBT on the acquisition of skills (i.e., moderated-mediation; Figure 5). In this model, baseline measures of cognitive function (*W*) moderate both the direct (c'_3) and indirect effects (a_3*b_1) of CBT (*X*) on PDA (*Y*); **(c)** (i) Using latent growth curve modeling outlined in Aim 2 we will explore differences in mediation across the two forms of CBT (clinician-delivered CBT vs. CBT4CBT) to evaluate the role of skills acquisition across formats; (ii) Additional potential mediators such as therapeutic alliance and self-efficacy[253] will be explored across CBT delivery formats using serial multiple mediation models[254]; (iii) Pre-treatment cognitive function (CANTAB) will be evaluated as a moderator of the treatment effect on alcohol use outcomes across CBT delivery formats, as well as on the mediated effect of skills acquisition (moderated-mediation across CBT formats). Additional potential moderator variables will be explored across CBT formats, such as client motivation (Stages of Change Questionnaire), alcohol use severity (AUDIT) and general intelligence (Shipley) using linear moderation regression models.

Figure 5.
Moderated Mediation

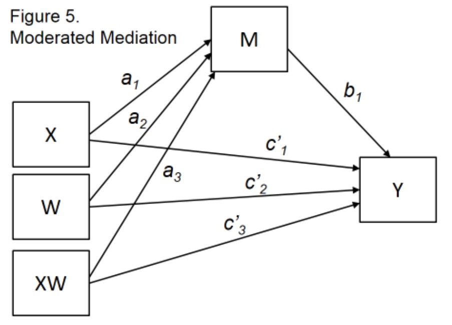

3. Genetic Testing N/A ☐

A. Describe

i. the types of future research to be conducted using the materials, specifying if immortalization of cell lines, whole exome or genome sequencing, genome wide association studies, or animal studies are planned

No immortalized cells will be utilized or established. Genomewide association studies will be conducted.

ii. the plan for the collection of material or the conditions under which material will be received

Saliva samples will be collected from study participants by study staff.

iii. the types of information about the donor/individual contributors that will be entered into a database

Demographic information, diagnostic information and assessment and outcome responses will be entered into a database.

iv. the methods to uphold confidentiality

There will be no personal identifiable information associated with the biological materials (saliva sample and DNA). The biological material from subjects will be identified by code rather than by personal identifiable information

B. What are the conditions or procedures for sharing of materials and/or distributing for future research projects?

Some de-identified genotype and phenotype data will be made available to qualified investigators in the research community, including distribution via an NIH-sponsored data repository

C. Is widespread sharing of materials planned?
   Yes, via an NIH-sponsored data repository.
D. When and under what conditions will materials be stripped of all identifiers?
   At the time of collection, biological materials (saliva sample and DNA) from subjects will be identified by a code number rather than by name or other personal identifier.
E. Can donor-subjects withdraw their materials at any time, and/or withdraw the identifiers that connect them to their materials?
   i. How will requests to withdraw materials be handled (e.g., material no longer identified: that is, anonymized) or material destroyed)?
      Donor-subjects can withdraw the identifiers that connect them to their biological materials at anytime. If a subject chooses to withdraw their identifiers, all identifiers and links to identifiers for the participant will be deleted and destroyed

F. Describe the provisions for protection of participant privacy
   Any records with identifiable patient information will be kept either in locked cabinets (hard copies), on encrypted removable computer media (electronic files) or on a password-protected, firewalled network server, and no such records will leave any clinical site.
G. Describe the methods for the security of storage and sharing of materials
   Extracted DNA samples will be stored in the Genetics Laboratory.

4. **Subject Population:** Provide a detailed description of the types of human subjects who will be recruited into this study.

Participants will be 180 individuals (aged 18 and older) seeking treatment for a primary alcohol use disorder at MCCA, which is an outpatient treatment facility, one of the largest providers of addiction treatment services in the Bridgeport-New Haven area or from SATU located in New Haven. In 2014, MCCA served 934 individuals with a substance use disorder (53%; n=491 with primary alcohol use disorder). The clinic population data indicate that 25% are female, and 60% represent ethnic minority groups (34% African American, 23% Hispanic, 3% other - primarily Asian or Native American). Thus, it is reasonable to anticipate we will recruit a sample with roughly these proportions of women and ethnic minorities, as we have in our previous trials.

5. **Subject classification:** Check off all classifications of subjects that will be specifically recruited for enrollment in the research project. Will subjects who may require additional safeguards or other considerations be enrolled in the study? If so, identify the population of subjects requiring special safeguards and provide a justification for their involvement.

☐ Children ☐ Healthy ☐ Fetal material, placenta, or dead fetus
☐ Non-English Speaking ☐ Prisoners ☐ Economically disadvantaged persons
☐ Decisionally Impaired ☐ Employees ☐ Pregnant women and/or fetuses
☐ Yale Students ☐ Females of childbearing potential

NOTE: Is this research proposal designed to enroll children who are wards of the state as potential subjects? ☐ Yes ☒ No (If yes, see Instructions section VII #4 for further requirements)

6. **Inclusion/Exclusion Criteria:** What are the criteria used to determine subject inclusion or exclusion?

Individuals will be included who:

- Are 18 years of age or older.
- Are applying for outpatient alcohol treatment and meet current DSM-5 criteria for alcohol use disorder, consuming at least 14/7 drinks (men/women) per week with at least 4 heavy drinking days reported in the past 28 days,
- Are sufficiently stable for 8 weeks of outpatient treatment and can commit to a 6-month follow-up
- Are willing to provide locator information for follow-up, and
- Are fluent in English and have a 6th grade or higher reading level.

Individuals will be excluded who:

- Have an untreated bipolar or schizophrenic disorder,
- Have a current legal case pending such that incarceration during the 8-week protocol is likely,
- *Have been prescribed an alcohol pharmacotherapy (e.g., disulfiram, naltrexone) within the past two weeks*, or
- Are physically dependent on alcohol, opioids or benzodiazepenes *such that immediate medical detoxification is necessary for safety purposes* (individuals demonstrating significant withdrawal symptoms would be eligible for re-screening following brief medical detoxification, which is arranged by MCCA or SATU staff at triage).

7. How will **eligibility** be determined, and by whom?

All new MCCA or SATU patients will be invited to participate in the protocol at the time of their initial intake appointment. They will be identified by their clinicians, interest sheets, and clinics postings. Those interested will be given a research packet containing the flyer, consent form, consent quiz and a self-addressed, stamped envelope for mailing the consent form and quiz back to the research team. Individuals who indicate they are interested in hearing more about the study will be offered a virtual meeting with the Research Coordinator by calling the study staff from the flyer. If potential participants prefer, with verbal consent to their clinicians, the clinician can give contact information to the research staff. At the first virtual interview, the Research Coordinator will provide a brief overview of the protocol and obtain written informed consent via Zoom using established guidelines of Yale University in which a photo is taken of the signature page of the consent form that the participant has received from the clinic in the research packet. The photo is taken by the research staff on the research cell phone. After determination of eligibility, the pretreatment assessments will be completed virtually.

8. **Risks:** Describe the reasonably foreseeable risks, including risks to subject privacy, discomforts, or inconveniences associated with subjects participating in the research.

1 Standard outpatient treatment: Treatment As Usual (TAU) and clinician-delivered CBT

The behavioral treatments used here, standard group counseling and clinician-delivered, NIAAA manual-guided Cognitive Behavioral Coping Skills Therapy (CBT)[125] at the MCCA and SATU clinics have been used safely in multiple previous studies at our centers and by investigators in other centers in the past. CBT will be delivered by highly trained clinicians who receive ongoing supervision and regular feedback/monitoring based on ratings of fidelity and skill by licensed study staff. Psychological risks are minimal and not different from those of equivalent non-study psychotherapeutic interventions[255].

For all treatment conditions, frequent monitoring (at least weekly) of the participant's clinical status by clinicians and research staff will insure identification and withdrawal from the study of participants who show significant psychological or symptomatic deterioration (*and thus may require a higher level of care*).

2. Computer-assisted training program: CBT4CBT

The 'CBT4CBT' program will be delivered in addition to brief (<10 minute) weekly clinical monitoring of safety. The clinical monitoring will be provided by a trained and supervised MCCA or SATU clinician *using our modified 'Clinical Management' manual*[185]*, following guidelines for low-intensity interventions used in previous placebo-controlled trials*[186-188] *and trials of internet-delivered treatment* [189]*. These brief monitoring sessions are for the purposes of ensuring client safety and will focus on assessment of clinical symptoms and level of risk for harm (e.g., suicide/homicide risk). The session will include an assessment of alcohol use frequency (for determination of significant increases in alcohol use), current severity of any psychiatric symptoms including suicidal/homicidal ideation, and determination of any immediate case management needs (with appropriate referral for case management services as needed – e.g., housing support, applications for financial assistance).*

The CBT4CBT program is modeled closely on the NIAAA and NIDA CBT treatment manuals[120, 125] that have been evaluated in several randomized clinical trials and widely accepted as an empirically validated treatment for alcohol use disorders. We have completed two randomized clinical trials of the program *for drug use disorders and one trial of the program for alcohol use disorders* and have found it safe and effective for a broad range of substance users[119, 122, 123], with no adverse events attributable to the program.
The web-based intervention is highly secure, and **does not collect any PHI identifiers nor specific information regarding illegal activities,** and has passed stringent Yale Information Technology Security Design Review. It is accessible only through a username/password system monitored by Yale staff. Moreover, the design of the CBT4CBT program has closely followed recommended ethical and safety guidelines for use of computer assisted behavioral therapies developed by Sampson and Pyle[256], including (1) assurance of confidentiality, (2) determination of appropriateness of the specific form of training, in this case, CBT, which has been shown to be effective for a wide number of substance use disorders and populations,

(3) adequate introduction to the computer program by staff to reduce possible anxiety about use of the system, (4) provision of follow-up consultation with a clinician if needed, and (5) supervision of the treatment process by a clinician[256, 257].

Computer-based interventions have been used safely in multiple investigations with a range of populations[117, 258-260] , and we are unaware of any reported risks associated with these interventions. A recent Agency for Healthcare Research and Quality (AHRQ) comprehensive review of computer-assisted interventions for a wide range of health and mental health issues found no reports of adverse events associated with these programs[110]. Similarly, the CBT4CBT program was not associated with any adverse events in our previous trials with a comparatively severe drug using population nor in our *completed* R21 pilot study with an alcohol using sample[119, 122, 123].

Thus, while we believe adverse events are likely to be rare, because the proposed trial includes an evaluation of the CBT4CBT version delivered as an approximation of a 'stand-alone' intervention, special efforts will be made to monitor patient safety and response closely. In addition to weekly assessments that include breath and urine monitoring taken on the schedule of the clinic and self-reports of substance use, the 'CBT4CBT' virtual standalone condition will be implemented with one 10-minute or less, manual-guided, clinician monitoring session per week delivered by a trained and supervised master's level MCCA or SATU clinician supervisor. This will enable us to monitor each participant's clinical status closely and thus address potential Human Subjects concerns associated with evaluation of a 'stand-alone' computer-based intervention for treatment seekers. *Participants assigned to this condition who show significant psychological or symptomatic deterioration will be withdrawn from this condition and referred immediately to the appropriate level of treatment (consistent with criteria for withdrawal from the other treatment conditions).*

These procedures are also consistent with recommended guidelines for monitoring adverse events in behavioral trials[177]. The Research Coordinator will supervise the participants' use and understanding of the program closely, and Dr. Kiluk, will be available to monitor patients' reactions to the program and answer any clinical issues. In addition, participants will be notified at both login and logoff that any feelings or thoughts that concern them should be discussed immediately with their clinician, the Research Coordinator, and/or Dr. Kiluk. Dr. Kiluk can be reached via cell phone to discuss any issues or concerns related to the computer-based intervention.

3. Urine, breath and saliva specimen collection
Urine and breath specimens are collected primarily as safeguards to participants. Saliva samples, collected for DNA analysis and should add no risks other than those normally associated with these procedures.

4. Rating scales and questionnaires
These are all non-invasive, should add no risk, and have been used without difficulty or any adverse events in our previous studies with this population. The major disadvantage is the time taken to complete them. Our past experience with these measures indicates that they are

APPROVED BY THE YALE UNIVERSITY IRB 2/15/2021

acceptable to patients. Careful efforts aimed at maintaining confidentiality have been effective in previous research, and only participants' study code numbers will be recorded on the forms themselves to protect confidentiality.

9. **Minimizing Risks:** Describe the manner in which the above-mentioned risks will be minimized.

As noted above, for the standard treatment and clinician-delivered CBT condition, psychological risks are minimal and not different from those of equivalent non-study psychotherapeutic interventions. Careful training and supervision of staff, including annual training in Human Subjects Protection and Good Clinical Practice will further minimize these risks.

In all conditions, we will closely monitor participant treatment response and safety through virtual weekly assessment sessions; these will include assessment of psychiatric status. Although in our experience this is a very rare event in behavioral trials, including those of computer-assisted therapy [156], participants who show significant deterioration (e.g., increased alcohol use or psychiatric symptoms that cannot be managed within the protocol, including significant suicidal or homicidal ideation) will be regarded as symptomatic failures, withdrawn from the treatment arm of the study, and referred for appropriate treatment (typically a more intensive level of care). The independent evaluator (Dr. O'Malley) will make the final determination as to whether a participant should be withdrawn from the treatment arm of the study. At the time of withdrawal, endpoint ratings will be made which include the full termination assessment battery.

Participants withdrawn from the treatment arm of the trial for these reasons or because they wish to withdraw from the study will be offered treatment as usual at the MCCA or SATU clinic or be referred to a higher level of care when appropriate. Private referral and/or hospitalization may also be offered according to the participants' needs and wishes.

For the **CBT4CBT condition**, no adverse consequences have been noted in our previous studies, nor in the general literature on computer-based and web-based interventions. For the CBT4CBT condition, the chief difference from the other conditions is in the level of interaction with a clinician. We have built in the following safeguards:

- Understanding of and use of the CBT4CBT program. The Research Coordinator will work with each participant to assure they are comfortable using the program and answer any concerns they may have. The 'checkout' page of each module reminds participants they can contact Dr. Kiluk at any time. For this efficacy trial, participants will be asked to access the program at the clinic once per week at the time of the weekly assessment. The program tracks, for each participant, time logged onto the program, modules accessed, progress through the program from session to session, completion of homework assignments, and learning of CBT principles through multiple choice tests after each module.
- Provision of monitoring and limited clinician support. Participants assigned to the CBT4CBT condition will also meet weekly with a trained MCCA or SATU clinician for a 'monitoring/support' visit per clinic policy. These brief (10 minutes maximum) monitoring/support manual-guided visits will take place at the time of the weekly

APPROVED BY THE YALE UNIVERSITY IRB 2/15/2021

assessment sessions and follow guidelines for low-intensity interventions used in previous placebo-controlled trials [159,210] and are intended to provide a means of closely monitoring each participants' status, inquire as to whether the participant has any questions or concerns, and assess availability and use of supports. We have modified our 'Clinical Management' manual [163], modeled on that used in Project Combine [160], and which has been used as a low-intensity monitoring/support condition in multiple previous trials, for this use. These sessions will be taped and monitored for fidelity.

- Participants in all conditions are also able to call the MCCA or SATU on-call clinician at any time should questions or concerns arise; emergency services are available through MCCA or SATU.

We have used similar procedures to monitor participant response and safety in several previous studies evaluating a range of types and intensities of behavioral therapies, as well as in our recently completed R21 pilot trial evaluating CBT4CBT for alcohol use disorders. As in the proposed study, participants in the R21 pilot trial (and all conditions) were monitored weekly by research staff; participants whose frequency or intensity of alcohol or drug use worsened or did not improve were evaluated by the Project Director for possible withdrawal from the treatment arm of the study with referral to inpatient or intensive day treatment (i.e., higher level of care). Of 24 participants assigned to the CBT4CBT condition with brief clinical monitoring in that study, only one was withdrawn from the treatment arm of the study and referred to intensive day treatment. No other adverse or serious adverse events were seen in that condition.

10. **Data and Safety Monitoring Plan:** Include an appropriate Data and Safety Monitoring Plan (DSMP) based on the investigator's risk assessment stated below. (Note: the HIC will make the final determination of the risk to subjects.) For more information, see the Instructions, page 24.
    a. What is the investigator's assessment of the overall risk level for subjects participating in this study? Minimal Risk
    b. If children are involved, what is the investigator's assessment of the overall risk level for the children participating in this study?
    c. Copy, paste, and then tailor an appropriate Data and Safety Monitoring Plan from http://www.yale.edu/hrpp/forms-templates/biomedical.html for
        i. Minimal risk
        ii. Greater than minimal/moderate risk
        iii. High risk

A Data and Safety Monitoring Board (DSMB) will monitor this project. This board, is already in place for the Yale Psychotherapy Development Center, monitors all of Dr. Kiluk's clinical trials. The DSMB is composed of Yale investigators (Drs. Feillin, McKee, Barry) who are independent of the proposed trial and experienced in various aspects of the conduct of clinical trials for the treatment of addictive disorders. We have developed a standard DSMB report form that is used in all Center and Center-related trials that summarizes, *on a quarterly basis*:

1. Recruitment, retention, and follow-up rates for the study and compares them to target rates.
2. Rates of data completeness and availability of primary outcome data

APPROVED BY THE YALE UNIVERSITY IRB 2/15/2021

APPROVED BY THE YALE UNIVERSITY IRB 2/15/2021

3. Occurrence of AEs and SAEs
4. Report of study progress since the last report.
5. Rates of recruitment of women, minorities, and children with respect to targets.

These reports are generated by the Data Manager each quarter, reviewed and signed by Dr. Kiluk prior to their submission to the DSMB. DSMB comments are documented and forwarded to the Yale IRB at the time of the annual review and re-approval.

Because the projected effect sizes may not be large enough for detection during interim analyses, we are not proposing a preliminary analysis of accumulating efficacy and feasibility data by treatment assignment. Instead, we propose to submit a quarterly report of aggregate data to the DSMB members that contains screening data, baseline demographics, retention data, serious adverse events data, as well as accrual status including projections, times to milestones, and any other data that will help in the assessment of the clinical trial. Based on this report, each DSMB member will complete a form making one of two recommendations: 1) continue recruitment as planned; or 2) schedule formal DSMB meeting immediately. If any DSMB member recommends a meeting, this will be scheduled within one week, minutes will be kept, the report will be reviewed with Dr. Kiluk, and the committee will vote on whether the study should: 1) continue recruitment unchanged; 2) continue with a protocol amendment; 3) stop recruiting pending further investigation. If, after this meeting, any DSMB member votes to stop recruitment or requests a protocol modification, the Yale IRB will be informed.

Participants who experience a significant psychiatric or medical problem requiring an overnight hospitalization at an acute care facility will be considered to have experienced an SAE. In general, most SAEs will result in inpatient care and thus in transfer from MCCA or SATU. All SAEs will result in the completion of an SAE Form and a verbal report within one hour to the Principal Investigator (Dr. Kiluk) and the MCCA Clinic Director, Victor Pittman, or the SATU Clinical Director, Donna LaPaglia. Within 24 hours, the following additional individuals will be informed: 1) all co-investigators; 2) the DSMB. All of these individuals will receive a copy of the SAE Form within one week at which point a decision will be made whether to convene a meeting of the DSMB. Adverse events that are serious and unanticipated and probably, possibly, or definitely related or adverse events occurring with greater frequency than anticipated will be reported to Yale Human Investigation Committee within 48 hours of discovery.

The procedures for SAE reporting include written documentation using the clinical notes related to the adverse event and specific forms detailing the event with a sign-off by all appropriate supervisory personnel. Communication of recommendations and decisions from all parties (DSMB, Yale Human Investigations Committee, and MCCA or SATU Administration) are made back to the investigator in a timely manner. We will report all protocol amendments or changes in the informed consent form to NIAAA as well as any temporary or permanent suspension of patient accrual.

d. For multi-site studies for which the Yale PI serves as the lead investigator**: N/A**

i. How will adverse events and unanticipated problems involving risks to subjects or others be reported, reviewed and managed?
ii. What provisions are in place for management of interim results?

APPROVED BY THE YALE UNIVERSITY IRB 2/15/2021

iii. What will the multi-site process be for protocol modifications?

11. **Statistical Considerations:** Describe the statistical analyses that support the study design.

Aim 1 - Evaluation of treatment effects:
The principal strategy for assessing the efficacy of the study treatments on outcome will be random effects regression models for continuously measured primary (e.g., percentage of days abstinent - PDA) and secondary (e.g., percentage of heavy drinking days, quality of life, etc.) outcomes. Our research group has used these methods to evaluate main and interaction effects, with appropriate covariates (e.g., retention, compliance with treatment) in multiple previous trials[121, 138, 142, 171, 172]. We will include treatment utilization data during the follow-up period as a covariate in analyses evaluating treatment effects through follow-up.

Analysis of treatment effects at post-treatment and follow-up will include the following contrasts:

- ***H1a:*** *Main effect for CBT on PDA by month during treatment*: Clinician-CBT and CBT4CBT>TAU.
- ***H1b:*** *Main effect for CBT on PDA by month through follow-up:* Clinician-CBT and CBT4CBT>TAU.

Adequacy of sample size: *H1a* – Using conventional effect size estimates, we determined that our proposed sample size of 60 per condition would be sufficient for detecting a small-medium effect (*d*=.35) of treatment on PDA using a random effects regression model (2 groups, PDA by month, 3 data points, ICC=.6, alpha=.05, beta=.80). *H1b* – The proposed sample is sufficient to detect a medium effect (*d*=.50) through the follow-up period with a random effect regression model (2 groups, PDA by month, 7 data points, ICC=.6, alpha=.05, beta=.80). These effect sizes are conservative, based on estimates from completed studies of CBT4CBT[119, 122].

Power calculations	Power	Estimated effect size	# of datapoints	Sample size per cell
H1a: (Clinician CBT + CBT4CBT stand-alone) v TAU, main effect for active treatment	.80	.35	3	60
H1b : (Clinician CBT + CBT4CBT stand-alone) v TAU, main effect for follow up	.80	.50	7	60

Aim 2 – Evaluation of skills acquisition as mediator of CBT effects:
Latent growth curve modeling will be the principal strategy for evaluating mediation using Mplus version 7[251]. The product of coefficients method[18, 19] will be used to determine the mediating effects of skills acquisition during the treatment period *on PDA* during follow-up. We will be using the 6-month period following treatment as our alcohol use outcome in this model to support the temporal relation criterion[64], as well as to evaluate the delayed emergence of CBT's effect on alcohol use[139]. This model will test:

- ***H2a:*** *Effect of CBT on acquisition of skills (a_2* path: *X→M):* Clinician-CBT>TAU; CBT4CBT>TAU
- ***H2b:*** Effect of change in acquisition *of skills on PDA by month during follow-up (b_2* path: *M→Y)*

- ***H2c:*** *Indirect effect of CBT on PDA via change in acquisition of skills* $(a_2*b_2 / se_{a2}*se_{b2})$

Adequacy of sample size: Parameter estimates from our initial trial of CBT4CBT that supported coping skills acquisition as a mediator of treatment effects on substance use[96] were used to estimate effect sizes of treatment condition on PDA via skills acquisition (a=.26; b=.59). Based on these effect sizes and power estimates for testing mediation derived from a simulation study[94], we will have adequate power (.80) to detect significant mediating effects with the proposed sample size of 60 per condition.

SECTION VI: RESEARCH INVOLVING DRUGS, BIOLOGICS, RADIOTRACERS, PLACEBOS AND DEVICES

If this section (or one of its parts, A or B) is not applicable, state N/A and delete the rest of the section.

A. DRUGS, BIOLOGICS and RADIOTRACERS N/A

SECTION VII: RECRUITMENT/CONSENT AND ASSENT PROCEDURES

1. **Targeted Enrollment: Give the number of subjects:**
   a. targeted for enrollment at Yale for this protocol 180
   b. If this is a multi-site study, give the total number of subjects targeted across all sites__N/A_

2. **Indicate recruitment methods below.** Attach copies of any recruitment materials that will be used.

☒ Flyers	☒ Internet/Web Postings	☐ Radio
☐ Posters	☐ Mass E-mail Solicitation	☐ Telephone
☐ Letter	☒ Departmental/Center Website	☐ Television
☐ Medical Record Review	☐ Departmental/Center Research Boards	☒ Newspaper
☐ Departmental/Center Newsletters	☐ Web-Based Clinical Trial Registries	
☐ YCCI Recruitment database	☐ Clinicaltrials.gov Registry (do not send materials to HIC)	

☒ Other (describe): Direct referral from MCCA or SATU triage clinicians, billboards, bus ads

3. **Recruitment Procedures:**
   a. Describe how potential subjects will be identified.
   b. Describe how potential subjects are contacted.
   c. Who is recruiting potential subjects?

   All new MCCA or SATU patients will be invited to participate in the protocol at the time of their initial triage appointment. They will be identified by their clinicians, interest sheets, and clinics postings. Individuals who indicate they are interested in hearing more about the study will be offered a virtual meeting with the Research Coordinator. Clinicaltrials.Gov will not be used as a recruitment tool because everyone the study is presented to must be applying for treatment at MCCA or SATU . Therefore, if a potential participant were to call

the study directly from ClinicalTrials.Gov they would be instructed to apply for treatment at MCCA or SATU in the usual manner and then be offered the study as indicated above. At the first virtual interview, the Research Coordinator will provide a brief overview of the protocol and obtain written informed consent via Zoom using established guidelines of Yale University in which a photo is taken of the signature page of the consent form. After determination of eligibility, the pretreatment assessments will be completed virtually.

4. Screening Procedures

a. Will email or telephone correspondence be used to screen potential subjects for eligibility prior to the potential subject coming to the research office? ☐ Yes ☒ No

b. If yes, identify below all health information to be collected as part of screening and check off any of the following HIPAA identifiers to be collected and retained by the research team during this screening process.

HEALTH INFORMATION TO BE COLLECTED:

HIPAA identifiers:

☐ Names
☐ All geographic subdivisions smaller than a State, including: street address, city, county, precinct, zip codes and their equivalent geocodes, except for the initial three digits of a zip code if, according to the current publicly-available data from the Bureau of the Census: (1) the geographic unit formed by combining all zip codes with the same three initial digits contains more than 20,000 people, and (2) the initial three digits of a zip code for all such geographic units containing 20,000 or fewer people is changed to 000.
☐ Telephone numbers
☐ Fax numbers
☐ E-mail addresses
☐ Social Security numbers
☐ Medical record numbers
☐ Health plan beneficiary numbers
☐ Account numbers
☐ All elements of dates (except year) for dates related to an individual, including: birth date, admission date, discharge date, date of death, all ages over 89 and all elements of dates (including year) indicative of such age, except that such ages and elements may be aggregated into a single category of age 90 or older
☐ Certificate/license numbers
☐ Vehicle identifiers and serial numbers, including license plate numbers
☐ Device identifiers and serial numbers
☐ Web Universal Resource Locators (URLs)
☐ Internet Protocol (IP) address numbers
☐ Biometric identifiers, including finger and voice prints
☐ Full face photographic images and any comparable images
☐ Any other unique identifying numbers, characteristics, or codes

5. Assessment of Current Health Provider Relationship for HIPAA Consideration:

Does the Investigator or any member of the research team have a direct existing clinical relationship with any potential subject?

☐ Yes, all subjects
☐ Yes, some of the subjects
☒ No

If yes, describe the nature of this relationship.

6. **Request for waiver of HIPAA authorization:** (When requesting a waiver of HIPAA Authorization for either the entire study, or for recruitment purposes only. Note: if you are collecting PHI as part of a phone or email screen, you must request a HIPAA waiver for recruitment purposes.)

   N/A

   Choose one: For entire study: ______ For recruitment purposes only: ______

   i. Describe why it would be impracticable to obtain the subject's authorization for use/disclosure of this data;
   ii. If requesting a waiver of **signed** authorization, describe why it would be impracticable to obtain the subject's signed authorization for use/disclosure of this data;

   By signing this protocol application, the investigator assures that the protected health information for which a Waiver of Authorization has been requested will not be reused or disclosed to any person or entity other than those listed in this application, except as required by law, for authorized oversight of this research study, or as specifically approved for use in another study by an IRB.

Researchers are reminded that unauthorized disclosures of PHI to individuals outside of the Yale HIPAA-Covered entity must be accounted for in the "accounting for disclosures log", by subject name, purpose, date, recipients, and a description of information provided. Logs are to be forwarded to the Deputy HIPAA Privacy Officer.

7. **Required HIPAA Authorization:** If the research involves the creation, use or disclosure of protected health information (PHI), separate subject authorization is required under the HIPAA Privacy Rule. Indicate which of the following forms are being provided:

   ☒ Compound Consent and Authorization form
   ☐ HIPAA Research Authorization Form

8. **Consent Personnel:** List the names of all members of the research team who will be obtaining consent/assent.

   Brian Kiluk, PhD
   Lawanda Frederick, BA
   Elizabeth Doohan, BA

9. **Process of Consent/Assent:** Describe the setting and conditions under which consent/assent will be obtained, including parental permission or surrogate permission and the steps taken to ensure subjects' independent decision-making.

   Individuals identified by MCCA or SATU clinicians will be offered the opportunity to meet with the Research Coordinator virtually at the time of triage or be given the research study telephone number to contact. At the virtual research and consent interview, the Research Coordinator will describe the requirements of the study and explain informed consent

procedures. Following provision of informed consent, Dr. Kiluk or the Research Coordinator will review inclusion and exclusion criteria and determine eligibility.

10. **Evaluation of Subject(s) Capacity to Provide Informed Consent/Assent:** Indicate how the personnel obtaining consent will assess the potential subject's ability and capacity to consent to the research being proposed.

    We routinely use an informed consent quiz to assure all prospective participants understand all aspects of the protocol and its requirements. This will be done virtually along with the other assessments. We also routinely collect urine and breath samples from all participants; if potential participants are determined to be under the influence of drugs or alcohol at the consent session, the informed consent session is rescheduled and the results from urine and breath screen are not formally recorded.

11. **Documentation of Consent/Assent:** Specify the documents that will be used during the consent/assent process. Copies of all documents should be appended to the protocol, in the same format that they will be given to subjects.

    HIC approved Compound Consent and Authorization Form and consent quiz

12. **Non-English Speaking Subjects:** Explain provisions in place to ensure comprehension for research involving non-English speaking subjects. Translated copies of all consent materials must be submitted for approval prior to use.

    As per inclusion criteria, reading and comprehension in English at a 6th grade level is required**.**

13. **Consent Waiver: In certain circumstances, the HIC may grant a waiver of signed consent, or a full waiver of consent, depending on the study.** If you will request either a waiver of consent, or a waiver of signed consent for this study, complete the appropriate section below.
    - ☒ **Not Requesting a consent waiver**
    - ☐ **Requesting a waiver of signed consent**
    - ☐ **Requesting a full waiver of consent**

A. <u>Waiver of **signed** consent</u>: (Verbal consent from subjects will be obtained. **If PHI is collected, information in this section must match Section VII, Question 6**)

☐ **Requesting a waiver of signed consent for <u>Recruitment/Screening</u> only**

If requesting a waiver of signed consent, please address the following:

a. Would the signed consent form be the only record linking the subject and the research?

☐ Yes ☐ No

b. Does a breach of confidentiality constitute the principal risk to subjects?

☐ Yes ☐ No

OR

c. Does the research activity pose greater than minimal risk?
☐ Yes ***If you answered yes, stop. A waiver cannot be granted.*** Please note: Recruitment/screening is generally a minimal risk research activity
☐ No

AND

d. Does the research include any activities that would require signed consent in a non-research context? ☐ Yes ☐ No

☐ **Requesting a waiver of signed consent for the Entire Study (**Note that an information sheet may be required.)

If requesting a waiver of signed consent, please address the following:
a. Would the signed consent form be the only record linking the subject and the research?
☐ Yes ☐ No
b. Does a breach of confidentiality constitute the principal risk to subjects?
☐ Yes ☐ No

OR

c. Does the research pose greater than minimal risk? ☐ Yes ***If you answered yes, stop. A waiver cannot be granted.*** ☐ No

AND

d. Does the research include any activities that would require signed consent in a non-research context? ☐ Yes ☐ No

B. **Full waiver** of consent: (No consent from subjects will be obtained for the activity.)
☐ **Requesting a waiver of consent for Recruitment/Screening only**
a. Does the research activity pose greater than minimal risk to subjects?
☐ Yes ***If you answered yes, stop. A waiver cannot be granted.*** Please note: Recruitment/screening is generally a minimal risk research activity
☐ No
b. Will the waiver adversely affect subjects' rights and welfare? ☐ Yes ☐ No
c. Why would the research be impracticable to conduct without the waiver?
d. Where appropriate, how will pertinent information be returned to, or shared with subjects at a later date?

☐ **Requesting a full waiver of consent for the Entire Study (Note: If PHI is collected, information here must match Section VII, question 6.)**

If requesting a full waiver of consent, please address the following:

a. Does the research pose greater than minimal risk to subjects?
☐ Yes ***If you answered yes, stop. A waiver cannot be granted.***
☐ No
b. Will the waiver adversely affect subjects' rights and welfare? ☐ Yes ☐ No

c. Why would the research be impracticable to conduct without the waiver?
d. Where appropriate, how will pertinent information be returned to, or shared with subjects at a later date?

SECTION VIII: PROTECTION OF RESEARCH SUBJECTS

Confidentiality & Security of Data:

a. What protected health information (medical information along with the HIPAA identifiers) about subjects will be collected and used for the research?
b. How will the research data be collected, recorded and stored?
c. How will the digital data be stored? ☐ CD ☐ DVD ☐ Flash Drive ☐ Portable Hard Drive ☒ Secured Server ☒ Laptop Computer ☒ Desktop Computer ☐ Other
d. What methods and procedures will be used to safeguard the confidentiality and security of the identifiable study data and the storage media indicated above during and after the subject's participation in the study?
Do all portable devices contain encryption software? ☒ Yes ☐ No
If no, see http://hipaa.yale.edu/guidance/policy.html
e. What will be done with the data when the research is completed? Are there plans to destroy the identifiable data? If yes, describe how, by whom and when identifiers will be destroyed. If no, describe how the data and/or identifiers will be secured.
f. Who will have access to the protected health information (such as the research sponsor, the investigator, the research staff, all research monitors, FDA, Yale Cancer Center Data and Safety Monitoring Committee (DSMC), SSC, etc.)? (please distinguish between PHI and de-identified data)
g. If appropriate, has a Certificate of Confidentiality been obtained?
Yes; CC-AA-16-044 expires 12/31/2022.

h. Are any of the study procedures likely to yield information subject to mandatory reporting requirements? (e.g. HIV testing – reporting of communicable diseases; parent interview -incidents of child abuse, elderly abuse, etc.). Please verify to whom such instances will need to be reported.

- Our study forms have been designed to avoid collecting identifiable information. We previously collected only birthdates and session dates. As of September 2007 this practice has been changed to collecting age. Session dates are changed to 'number of sessions completed' if data sets are released to other Yale investigators.
- Research data are collected on CRFs, and sent to data managers in our research offices on a closed secure network. All computers used by research staff are password protected. No identifying information is on CRFs.
- The screening of patients using the inclusion and exclusion criteria, and the comprehensive evaluations will minimize the risk of including subjects with insignificant substance use (or who are otherwise inappropriate for the study).
- Confidentiality in regards to collected materials will be maintained via a numbered reference system maintained by the Research Coordinator. Participants' names will appear only on the consent form, HIPPA authorization form, and "key" form kept by the Research Coordinator.

- Limits to confidentiality include only disclosure of acute suicidality, homicidality, or abuse of a minor, as is standard in clinical practice.
- Data are stored at our secure data management center; data sets do not include identifying information. At the conclusion of the study, all locator data are destroyed.
- The funding agency, NIAAA, may access the data for routine audits.
- We have received a Certificate of Confidentiality.
- All research staff and clinicians receive annual Good Clinical Practice training through the Core.

Our data collection and management procedures are fully compliant with HIPAA

Information about participants and their health, which might identify them, may be given to the following for audit and oversight reasons

- Regional Network of Programs
- MCCA
- The Substance Abuse Treatment Center (SATU)
- Yale University School of Medicine
- National Institute of Health (NIH). The research sponsor
- National Institute on Alcohol Abuse and Alcoholism (NIAAA)
- Members of the Human Investigations Committee or ethics Committee(s)
- Key Investigators
- Key Study Personnel
- Data and Safety Monitoring Board and others authorized to monitor the conduct of the study

SECTION IX: POTENTIAL BENEFITS

Potential Benefits: Identify any benefits that may be reasonably expected to result from the research, either to the subject(s) or to society at large. (Payment of subjects is not considered a benefit in this context of the risk benefit assessment.)

Benefits to participants include significant psychotherapeutic exploration through the provision of study therapies. Psychiatric examinations are also potential benefits. The major potential benefit in this study is in reduction of alcohol use via the study treatments, which may, in turn, foster improvement in participants' legal, medical, interpersonal, psychological and occupational functioning.

SECTION X: RESEARCH ALTERNATIVES AND ECONOMIC CONSIDERATIONS

1. **Alternatives:** What other alternatives are available to the study subjects outside of the research?
   Regular clinical care at MCCA or SATU

2. **Payments for Participation (Economic Considerations):** Describe any payments that will be made to subjects, the amount and schedule of payments, and the conditions for receiving this compensation.

All subjects will be offered a material inducement for participation in study evaluations in the form of either gift card or cash including $25 for screening, $50 for pretreatment, $10 for each weekly assessment completed in the treatment phase of the study ($10 per week), $50 for week 4 assessments, $50 at the end of the study (week 8) with a bonus of $25 for completing on time, and $75, $100, and $125 for each completed follow-up interview (1-, 3-, and 6-months, respectively)with a $25 bonus at each timepoint for completing assessments on time. Completion of follow-up interviews will be paid in gift card form if the participant is still enrolled in treatment at MCCA or SATU at the time of the interview. The maximum compensation available for the study is $655 if all assessments are completed. If participants do not complete all assessments they will be paid on a prorated basis for those they complete.

Participants who choose to participate in the optional genetics sub study will receive $15.

Subject Compensation Schedule

Activity	***Compensation Available***	***Form of Payment***
Screening	$25	Gift Card
Pretreatment	$50	Gift Card
Active Sessions (1x/wk for 8 weeks)	$80 Total (@ $10 a session)	Gift Card
Week 4 Assesments	$50	Gift Card
Post (end of study, week 8)	$75 ($50+ $25 bonus if completed on time)	Gift Card
Follow up 1 Month	$100 ($75+ $25 bonus if completed on time)	Gift card
Follow up 3 Months	$125 ($100+ $25 bonus if completed on time)	Gift card
Follow up 6 Months	$150 ($125+ $25 bonus if completed on time)	Gift card
Total Available	$655	

The major potential benefit in this study is in reduction of alcohol use via the study treatments, which may, in turn, foster improvement in subjects legal, medical, interpersonal, psychological and occupational functioning.

3. **Costs for Participation (Economic Considerations):** Clearly describe the subject's costs associated with participation in the research, and the interventions or procedures of the study that will be provided at no cost to subjects.

Subjects will not be charged for study treatments (CBT4CBT program) or research evaluations they receive at the clinic. Subjects may be charged for treatment as usual at the clinic; but most patients receive treatment with no-out of pocket expenses or on a sliding scale. There will be no monetary cost to participants associated with this research protocol.

4. **In Case of Injury:** This section is required for any research involving more than minimal risk.
   a. Will medical treatment be available if research-related injury occurs?

b. Where and from whom may treatment be obtained?
c. Are there any limits to the treatment being provided?
d. Who will pay for this treatment?
e. How will the medical treatment be accessed by subjects?

Because we are evaluating standard behavioral approaches with strong empirical support and no known adverse consequences, study related injuries are expected to be extremely rare. There will be no compensation and/or medical treatment available if injury occurs. Participants or their insurance carrier will be expected to cover costs of any medical treatment

APPROVED BY THE YALE UNIVERSITY IRB 2/15/2021

LITERATURE CITED

1. Morgenstern J, Naqvi NH, Debellis R, and Breiter HC. The contributions of cognitive neuroscience and neuroimaging to understanding mechanisms of behavior change in addiction. *Psychol Addict Behav,* 2013. 27(2): p. 336-50.
2. Team UR. UK Alcohol Treatment Trial: client-treatment matching effects. *Addiction,* 2008. 103(2): p. 228-38.
3. Project MATCH Research Group. Matching alcoholism treatments to client heterogeneity: Treatment main effects and matching effects on drinking during treatment. *Journal of Studies on Alcohol,* 1998. 59: p. 631-639.
4. Sobell LC and Sobell MB. *Timeline followback: A technique for assessing self-reported alcohol consumption*, in *Measuring alcohol consumption: Psychosocial and biological methods*, R.Z. Litten and J. Allen, Editors. 1992, Humana Press: New Jersey. p. 41-72.
5. Sobell LC and Sobell MB. *Alcohol Timeline Followback (TLFB)*, in *Handbook of Psychiatric Measures*. 2000, American Psychiatric Association: Washington, DC. p. 477-479.
6. Allen JP. Measuring outcome in interventions for alcohol dependence and problem drinking: executive summary of a conference sponsored by the national institute on alcohol abuse and alcoholism. *Alcohol Clin Exp Res,* 2003. 27(10): p. 1657-60.
7. Sobell LC, Sobell MB, Connors GJ, and Agrawal S. Assessing drinking outcomes in alcohol treatment efficacy studies: selecting a yardstick of success. *Alcohol Clin Exp Res,* 2003. 27(10): p. 1661-6.
8. Wetterling T, Dibbelt L, Wetterling G, Goder R, Wurst F, Margraf M, and Junghanns K. Ethyl glucuronide (EtG): better than breathalyser or self-reports to detect covert short-term relapses into drinking. *Alcohol Alcohol,* 2014. 49(1): p. 51-4.
9. Junghanns K, Graf I, Pfluger J, Wetterling G, Ziems C, Ehrenthal D, Zollner M, Dibbelt L, Backhaus J, Weinmann W, and Wurst FM. Urinary ethyl glucuronide (EtG) and ethyl sulphate (EtS) assessment: valuable tools to improve verification of abstention in alcohol-dependent patients during in-patient treatment and at follow-ups. *Addiction,* 2009. 104(6): p. 921-6.
10. Jatlow PI, Agro A, Wu R, Nadim H, Toll BA, Ralevski E, Nogueira C, Shi J, Dziura JD, Petrakis IL, and O'Malley SS. Ethyl glucuronide and ethyl sulfate assays in clinical trials, interpretation, and limitations: results of a dose ranging alcohol challenge study and 2 clinical trials. *Alcohol Clin Exp Res,* 2014. 38(7): p. 2056-65.
11. Feinn R, Tennen H, and Kranzler HR. Psychometric properties of the Short Index of Problems as a measure of recent alcohol-related problems. *Alcoholism: Clinical and Experimental Research,* 2003. 27(9): p. 1436-1441.
12. Kiluk BD, Dreifuss JA, Weiss RD, Morgenstern J, and Carroll KM. The Short Inventory of Problems-Revised (SIP-R): Psychometric properties within a large, diverse sample of substance use disorder treatment seekers. *Psychology of Addictive Behaviors,* 2013. 27(1): p. 307-314. PMCID: *PMC3434306.*
13. World Health Organization Quality of Life Group. Development of the World Health Organization WHOQOL-BREF quality of life assessment. The WHOQOL Group. *Psychol Med,* 1998. 28(3): p. 551-8.

14. Olmstead TA, Ostrow CD, and Carroll KM. Cost-effectiveness of computer-assisted training in cognitive-behavioral therapy as an adjunct to standard care for addiction. *Drug Alcohol Depend,* 2010. 110(3): p. 200-7.
15. Carroll KM, Nich C, Frankforter TL, and Bisighini RM. Do patients change in the way we intend? Treatment-specific skill acquisition in cocaine-dependent patients using the Cocaine Risk Response Test. *Psychological Assessment,* 1999. 11: p. 77-85.
16. Litt MD, Kadden RM, Cooney NL, and Kabela E. Coping skills and treatment outcomes in cognitive behavioral and interactional group therapy for alcoholism. *Journal of Consulting and Clinical Psychology,* 2003. 71: p. 118-128.
17. Sugarman DE, Nich C, and Carroll KM. Coping strategy use following computerized cognitive-behavioral therapy for substance use disorders. *Psychol Addict Behav,* 2010. 24(4): p. 689-95.
18. MacKinnon DP, Lockwood CM, Hoffman JM, West SG, and Sheets V. A comparison of methods to test mediation and other intervening variable effects. *Psychol Methods,* 2002. 7(1): p. 83-104.
19. MacKinnon DP, Fairchild AJ, and Fritz MS. Mediation analysis. *Annu Rev Psychol,* 2007. 58: p. 593-614.
20. Robbins TW, James M, Owen AM, Sahakian BJ, Lawrence AD, McInnes L, and Rabbitt PM. A study of performance on tests from the CANTAB battery sensitive to frontal lobe dysfunction in a large sample of normal volunteers: implications for theories of executive functioning and cognitive aging. Cambridge Neuropsychological Test Automated Battery. *J Int Neuropsychol Soc,* 1998. 4(5): p. 474-90.
21. Miller WR and Wilbourne PL. Mesa Grande: A methodological analysis of clinical trials of treatments for alcohol use disorders. *Addiction,* 2002. 97: p. 265-277.
22. Magill M and Ray LA. Cognitive-behavioral treatment with adult alcohol and illicit drug users: A meta-analysis of randomized controlled trials. *Journal of Studies on Alcohol and Drugs,* 2009. 70(4): p. 516-527.
23. Project MATCH Research Group. Matching alcoholism treatments to client heterogeneity: treatment main effects and matching effects on drinking during treatment. *J Stud Alcohol,* 1998. 59(6): p. 631-9.
24. Roth A and Fonagy P. *What Works for Whom? A Critical Review of the Psychotherapy Literature, Second Edition*. 2005, New York: The Guilford Press.
25. Miller WR, Brown JM, Simpson TL, Handmaker NS, Bien TH, Luckie LF, Montgomery HA, Hester RK, and Tonigan JS. *What works? A methodological analysis of the alcohol treatment literature.*, in *Handbook of Alcoholism Treatment Approaches: Effective Alternatives*, R.K. Hester and W.R. Miller, Editors. 1995, Allyn & Bacon: Boston, MA. p. 12-44.
26. Feeney GF, Young RM, Connor JP, Tucker J, and McPherson A. Cognitive behavioural therapy combined with the relapse-prevention medication acamprosate: are short-term treatment outcomes for alcohol dependence improved? *Aust N Z J Psychiatry,* 2002. 36(5): p. 622-8.
27. Balldin J, Berglund M, Borg S, Mansson M, Bendtsen P, Franck J, Gustafsson L, Halldin J, Nilsson LH, Stolt G, and Willander A. A 6-month controlled naltrexone study: combined effect with cognitive behavioral therapy in outpatient treatment of alcohol dependence. *Alcohol Clin Exp Res,* 2003. 27(7): p. 1142-9.

28. Anton RF, O'Malley SS, Ciraulo DA, Cisler RA, Couper D, Donovan DM, Gastfriend DR, Hosking JD, Johnson BA, LoCastro JS, Longabaugh R, Mason BJ, Mattson ME, Miller WR, Pettinati HM, Randall CL, Swift RM, Weiss RD, Williams LD, Zweben A, and for the COMBINE Study Research Group. Combined pharmacotherapies and behavioral interventions for alcohol dependence: The COMBINE Study. *JAMA,* 2006. 2006: p. 2003-2017.
29. Oslin DW, Lynch KG, Pettinati HM, Kampman KM, Gariti P, Gelfand L, Ten Have T, Wortman S, Dundon W, Dackis C, Volpicelli JR, and O'Brien CP. A placebo-controlled randomized clinical trial of naltrexone in the context of different levels of psychosocial intervention. *Alcohol Clin Exp Res,* 2008. 32(7): p. 1299-308.
30. Feeney GF, Connor JP, Young RM, Tucker J, and McPherson A. Combined acamprosate and naltrexone, with cognitive behavioural therapy is superior to either medication alone for alcohol abstinence: a single centres' experience with pharmacotherapy. *Alcohol Alcohol,* 2006. 41(3): p. 321-7.
31. Anton RF, Moak DH, Latham P, Waid LR, Myrick H, Voronin K, Thevos A, Wang W, and Woolson R. Naltrexone combined with either cognitive behavioral or motivational enhancement therapy for alcohol dependence. *J Clin Psychopharmacol,* 2005. 25(4): p. 349-57.
32. O'Malley SS, Rounsaville BJ, Farren C, Namkoong K, Wu R, Robinson J, and O'Connor PG. Initial and maintenance naltrexone treatment for alcohol dependence using primary care vs specialty care: a nested sequence of 3 randomized trials. *Arch Intern Med,* 2003. 163(14): p. 1695-704.
33. O'Malley SS, Jaffe AJ, Chang G, Schottenfeld RS, Meyer RE, and Rounsaville B. Naltrexone and coping skills therapy for alcohol dependence. A controlled study. *Arch Gen Psychiatry,* 1992. 49(11): p. 881-7.
34. DeRubeis RJ and Crits-Christoph P. Empirically supported individual and group psychological treatments for adult mental disorders. *Journal of Consulting and Clinical Psychology,* 1998. 66: p. 37-52.
35. Nathan PE and Gorman JM. *A Guide to Treatments That Work, Second Edition*. 2002, New York: Oxford University Press.
36. Tolin DF. Is cognitive-behavioral therapy more effective than other therapies? A meta-analytic review. *Clinical Psychology Review,* 2010. 30(6): p. 710-720.
37. O'Malley SS, Jaffe AJ, Chang G, Rode S, Schottenfeld R, Meyer RE, and Rounsaville BJ. Six month follow-up of naltrexone and psychotherapy for alcohol dependence. *Archives of General Psychiatry,* 1996. 53: p. 217-224.
38. Donovan DM, Anton RF, Miller WR, Longabaugh R, Hosking JD, Youngblood M, and Group CSR. Combined pharmacotherapies and behavioral interventions for alcohol dependence (The COMBINE Study): examination of posttreatment drinking outcomes. *J Stud Alcohol Drugs,* 2008. 69(1): p. 5-13.
39. Institute of Medicine. *Bridging the Gap Between Practice and Research: Forging Partnerships with Community-Based Drug and Alcohol Treatment.* 1998, Washington, DC: National Academy Press.
40. McLellan AT, Carise D, and Kleber HD. Can the national addiction treatment infrastructure support the public's demand for quality care? *Journal of Substance Abuse Treatment,* 2003. 25: p. 117-121.

41. Olmstead TA, Abraham AJ, Martino S, and Roman PM. Counselor training in several evidence-based psychosocial addiction treatments in private US substance abuse treatment centers. *Drug Alcohol Depend,* 2012. 120(1-3): p. 149-54.
42. Manuel JK, Hagedorn HJ, and Finney JW. Implementing evidence-based psychosocial treatment in specialty substance use disorder care. *Psychol Addict Behav,* 2011. 25(2): p. 225-37.
43. Miller WR, Sorensen JL, Selzer JA, and Brigham GS. Disseminating evidence-based practices in substance abuse treatment: A review with suggestions. *Journal of Substance Abuse Treatment,* 2006. 31: p. 25-39.
44. Miller WR. Bringing addiction treatment out of the closet. *Addiction,* 2007. 102: p. 867.
45. Carroll KM and Rounsaville BJ. A vision of the next generation of behavioral therapies research in the addictions. *Addiction,* 2007. 102(6): p. 850-62; discussion 863-9. PMCID: PMC2148498.
46. McCarty D, Fuller B, Kaskutas LA, Wendt WW, Nunes EV, Miller M, Forman R, Magruder KM, Arfken C, Copersino M, Floyd A, Sindelar J, and Edmundson E. Treatment programs in the National Drug Abuse Treatment Clinical Trials Network. *Drug and Alcohol Dependence,* 2008. 92(1-3): p. 200-7.
47. McCarty D, Fuller BE, Arfken C, Miller M, Nunes EV, Edmundson E, Copersino M, and Floyd A. Direct care workers in the National Drug Abuse Treatment Clinical Trials Network: Characteristics, opinions, and beliefs. *Psychiatric Services,* 2007. 58: p. 181-190.
48. Ball SA, Bachrach K, DeCarlo J, Farentinos C, Keen M, McSherry T, Polcin D, Snead N, Sockriter R, Wrigley P, Zammarelli L, and Carroll KM. Characteristics of community clinicians trained to provide manual-guided therapy for substance abusers. *Journal of Substance Abuse Treatment,* 2002. 23: p. 309-318.
49. Santa Ana E, Martino S, Ball SA, Nich C, and Carroll KM. What is usual about 'treatment as usual': Audiotaped ratings of standard treatment in the Clinical Trials Network. *Journal of Substance Abuse Treatment,* 2008. 35: p. 369-379.
50. Sholomskas D, Syracuse G, Ball SA, Nuro KF, Rounsaville BJ, and Carroll KM. We don't train in vain: A dissemination trial of three strategies for training clinicians in cognitive behavioral therapy. *Journal of Consulting and Clinical Psychology,* 2005. 73(1): p. 106-115. PMCID: PMC2367057.
51. Sholomskas DE and Carroll KM. One small step for manuals: Computer-assisted training in twelve-step facilitation. *Journal of Studies on Alcohol,* 2006. 67(6): p. 939-945. PMCID: PMC2366904.
52. Miller WR, Yahne CE, Moyers TB, Martinez J, and Pirritano M. A randomized trial of methods to help clinicians learn motivation interviewing. *Journal of Consulting and Clinical Psychology,* 2004. 72(6): p. 1050-1062.
53. McLellan AT. *What we need is a system: Creating a responsive and effective substance abuse treatment system*, in *Rethinking Substance Abuse: What the Science Shows and What We Should Do About It*, W.R. Miller and K.M. Carroll, Editors. 2006, Guilford: New York. p. 275-292.
54. Crits-Christoph P, Frank E, Chambless DL, Brody C, and Karp JF. Training in empirically validated therapies: What are clinical psychology students learning? *Professional Psychology: Research and Practice,* 1995. 26: p. 514-522.

55. Kazdin AE. Mediators and mechanisms of change in psychotherapy research. *Annu Rev Clin Psychol,* 2007. 3: p. 1-27.
56. Kazdin AE. Evidence-based treatment research: Advances, limitations, and next steps. *Am Psychol,* 2011. 66(8): p. 685-98.
57. Longabaugh R, Donovan DM, Karno MP, McCrady BS, Morgenstern J, and Tonigan JS. Active ingredients: how and why evidence-based alcohol behavioral treatment interventions work. *Alcohol Clin Exp Res,* 2005. 29(2): p. 235-47.
58. Morgenstern J and McKay JR. Rethinking the paradigms that inform behavioral treatment research for substance use disorders. *Addiction,* 2007. 102(9): p. 1377-89.
59. Longabaugh R, Donovan DM, Karno MP, McCrady BS, Morgenstern J, and Tonigan JS. Active ingredients: How and why evidence-based alcohol behavioral treatment interventions work. *Alcoholism: Clinical & Experimental Research,* 2005. 29: p. 235-247.
60. Abrams DB and Niaura RS. *Social learning theory*, in *Psychological Theories of Drinking and Alcoholism*, H.T. Blane and K.E. Leonard, Editors. 1987, Guilford: New York. p. 131-178.
61. Marlatt GA and Gordon JR. *Relapse Prevention: Maintenance Strategies in the Treatment of Addictive Behaviors*. 1985, New York: Guilford.
62. Miller WR, Westerberg VS, Harris RJ, and Tonigan JS. What predicts relapse? Prospective testing of antecedent models. *Addiction,* 1996. 91 Suppl: p. S155-72.
63. Sofuoglu M, DeVito EE, Waters AJ, and Carroll KM. Cognitive enhancement as a treatment for drug addictions. *Neuropharmacology,* 2013. 64: p. 452-63.
64. Kazdin AE and Nock MK. Delineating mechanisms of change in child and adolescent therapy: Methodological issues and research recommendations. *Journal of Child Psychology & Psychiatry,* 2003. 44: p. 1116-1129.
65. Nock MK. Conceptual and design essentials for evaluating mechanisms of change. *Alcohol Clin Exp Res,* 2007. 31(10 Suppl): p. 4s-12s.
66. Apodaca TR and Longabaugh R. Mechanisms of change in motivational interviewing: a review and preliminary evaluation of the evidence. *Addiction,* 2009. 104(5): p. 705-15.
67. Karno MP. A case study of mediators of treatment effectiveness. *Alcohol Clin Exp Res,* 2007. 31(10 Suppl): p. 33s-39s.
68. Morgenstern J and Longabaugh R. Cognitive-behavioral treatment for alcohol dependence: A review of the evidence for its hypothesized mechanisms of action. *Addiction,* 2000. 95: p. 1475-1490.
69. Baron RM and Kenny DA. The moderator-mediator variable distinction in social psychological research: conceptual, strategic, and statistical considerations. *J Pers Soc Psychol,* 1986. 51(6): p. 1173-82.
70. Finney JW, Noyes CA, Coutts AI, and Moos RH. Evaluating substance abuse treatment process models: I. Changes on proximal outcome variables during 12-step and cognitive-behavioral treatment. *J Stud Alcohol,* 1998. 59(4): p. 371-80.
71. Maisto SA, Connors GJ, and Zywiak WH. Alcohol treatment, changes in coping skills, self-efficacy, and levels of alcohol use and related problems 1 year following treatment initiation. *Psychol Addict Behav,* 2000. 14(3): p. 257-66.

APPROVED BY THE YALE UNIVERSITY IRB 2/15/2021

72. Perepletchikova F, Treat TA, and Kazdin AE. Treatment integrity in psychotherapy research: Analysis of the studies and examination of the associated factors. *Journal of Consulting & Clinical Psychology,* 2007. 75(6): p. 829-841.
73. Perepletchikova F and Kazdin AE. Treatment integrity and therapuetic change: Issues and research recommendations. *Clinical Psychology: Science and Practice,* 2005. 12: p. 365-383.
74. Miller WR and Rollnick S. The effectiveness and ineffectiveness of complex behavioral interventions: impact of treatment fidelity. *Contemp Clin Trials,* 2014. 37(2): p. 234-41.
75. Carroll KM, Nich C, Sifry R, Frankforter T, Nuro KF, Ball SA, Fenton LR, and Rounsaville BJ. A general system for evaluating therapist adherence and competence in psychotherapy research in the addictions. *Drug and Alcohol Dependence,* 2000. 57: p. 225-238.
76. Miller WR and Moyers TB. The forest and the trees: relational and specific factors in addiction treatment. *Addiction,* 2014.
77. Luborsky L, McLellan AT, Woody GE, O'Brien CP, and Auerbach A. Therapist success and its determinants. *Arch Gen Psychiatry,* 1985. 42(6): p. 602-11.
78. Crits-Christoph P, Baranackie K, Kurcias J, Beck AT, Carroll KM, Perry K, Luborsky L, McLellan AT, Woody G, Thompson L, Gallagher D, and Zitrin C. Meta-analysis of therapist effects in psychotherapy outcome studies. *Psychotherapy Research,* 1991. 1: p. 81-91.
79. Luborsky L, Crits-Christoph P, McLellan AT, Woody G, Piper W, Liberman B, Imber S, and Pilkonis P. Do therapists vary much in their success? Findings from four outcome studies. *Am J Orthopsychiatry,* 1986. 56(4): p. 501-12.
80. McLellan AT, Woody GE, Luborsky L, and Goehl L. Is the counselor an "active ingredient" in substance abuse rehabilitation? An examination of treatment success among four counselors. *J Nerv Ment Dis,* 1988. 176(7): p. 423-30.
81. Project MATCH Research Group. Therapist effects in three treatments for alcohol problems. *Psychotherapy Research,* 1998. 8(4): p. 455-474.
82. Kazdin AE. Treatment Outcomes, Common Factors, and Continued Neglect of Mechanisms of Change. *Clinical Psychology: Science and Practice,* 2005. 12(2): p. 184-188.
83. McKay JR. Lessons learned from psychotherapy research. *Alcohol Clin Exp Res,* 2007. 31(10 Suppl): p. 48s-54s.
84. Ambrose ML, Bowden SC, and Whelan G. Working memory impairments in alcohol-dependent participants without clinical amnesia. *Alcohol Clin Exp Res,* 2001. 25(2): p. 185-91.
85. Bates ME, Buckman JF, and Nguyen TT. A role for cognitive rehabilitation in increasing the effectiveness of treatment for alcohol use disorders. *Neuropsychology Review,* 2013.
86. Oscar-Berman M and Marinkovic K. Alcohol: effects on neurobehavioral functions and the brain. *Neuropsychol Rev,* 2007. 17(3): p. 239-57.
87. Bartley PC and Rezvani AH. Alcohol and cognition - consideration of age of initiation, usage patterns and gender: a brief review. *Curr Drug Abuse Rev,* 2012. 5(2): p. 87-97.

APPROVED BY THE YALE UNIVERSITY IRB 2/15/2021

88. Kopera M, Wojnar M, Brower K, Glass J, Nowosad I, Gmaj B, and Szelenberger W. Cognitive functions in abstinent alcohol-dependent patients. *Alcohol,* 2012. 46(7): p. 665-71.
89. Pitel AL, Witkowski T, Vabret F, Guillery-Girard B, Desgranges B, Eustache F, and Beaunieux H. Effect of episodic and working memory impairments on semantic and cognitive procedural learning at alcohol treatment entry. *Alcohol Clin Exp Res,* 2007. 31(2): p. 238-48.
90. Bates ME, Pawlak AP, Tonigan JS, and Buckman JF. Cognitive impairment influences drinking outcome by altering therapeutic mechanisms of change. *Psychology of Addictive Behaviors,* 2006. 20(3): p. 241-53.
91. Morgenstern J and Bates ME. Effects of executive function impairment on change processes and substance use outcomes in 12-step treatment. *J Stud Alcohol,* 1999. 60(6): p. 846-55.
92. Buckman JF, Bates ME, and Morgenstern J. Social support and cognitive impairment in clients receiving treatment for alcohol- and drug-use disorders: a replication study. *J Stud Alcohol Drugs,* 2008. 69(5): p. 738-46.
93. Blume AW and Marlatt GA. The role of executive functions in changing substance use: What we know and what we need to know. *Annals of Behavioral Medicine,* 2009. 37(2): p. 117-125.
94. Fritz MS and Mackinnon DP. Required sample size to detect the mediated effect. *Psychol Sci,* 2007. 18(3): p. 233-9.
95. Preacher KJ. Advances in mediation analysis: A survey and synthesis of new developments. *Annu Rev Psychol,* 2014.
96. Kiluk BD, Nich C, Babuscio T, and Carroll KM. Quantity vs. quality: Acquisition of coping skills following computerized cognitive behavioral therapy for substance use disorders. *Addiction,* 2010. 105: p. 2120-2127. PMCID: *PMC2975828.*
97. Litt MD, Kadden RM, Kabela-Cormier E, and Petry NM. Coping skills training and contingency management treatments for marijuana dependence: Exploring mechanisms of behavior change. *Addiction,* 2008. 103(4): p. 638-648.
98. Petry NM, Litt MD, Kadden R, and Ledgerwood DM. Do coping skills mediate the relationship between cognitive-behavioral therapy and reductions in gambling in pathological gamblers? *Addiction,* 2007. 102: p. 1280-1291.
99. Litt MD, Shafer DM, Ibanez CR, Kreutzer DL, and Tawfik-Yonkers Z. Momentary pain and coping in temporomandibular disorder pain: exploring mechanisms of cognitive behavioral treatment for chronic pain. *Pain,* 2009. 145(1-2): p. 160-8.
100. Greist JH. A promising debut for computerized therapies. *American Journal of Psychiatry,* 2008. 165(7): p. 793-795.
101. Carroll KM and Rounsaville BJ. Computer-assisted therapy in psychiatry: Be brave - it's a new world. *Current Psychiatry Reports,* 2010. 12(5): p. 426-432.
102. Institute of Medicine. *Broadening the Base of Treatment for Alcohol Problems*. 1990, Washington, D.C.: National Academy Press.
103. Babor TF, McKee BG, Kassenbaum PA, Grimaldi PL, Ahmed K, and Bray J. Screening, Brief Intervention, and Referral to Treatment (SBIRT): toward a public health approach to the management of substance abuse. *Substance Abuse,* 2007. 28: p. 7-30.

104. Norquist G and Regier DA. The epidemiology of psychiatric disorders and the de facto mental health care system. *Annual Review of Medicine,* 1996. 47: p. 473-479.
105. Postel MG, de Jong CA, and de Haan HA. Does e-therapy for problem drinking reach hidden populations? *Am J Psychiatry,* 2005. 162(12): p. 2393.
106. Marks IM and Cavanagh K. Computer-aided psychological treatments: Evolving issues. *Annual Review of Clinical Psychology,* 2009. 5(121-141).
107. Cunningham JA. Access and interest: Two important issues in considering the feasibility of web-assisted tobacco interventions. *Journal of Medical Internet Research,* 2008. 10(5): p. e37.
108. Cuijpers P, van Straten A, and Andersson G. Internet-administered cognitive behavior therapy for health problems: A systematic review. *Journal of Behavioral Medicine,* 2008. 31: p. 169-177.
109. Budman SH. Behavioral health care dot-com and beyond: Computer-mediated communications in mental health and substance abuse treatment. *American Psychologist,* 2000. 55: p. 1290-1300.
110. Gibbons MC, Wilson RF, Samal L, Lehmann CU, Dickersin K, Aboumatar H, Finkelstein J, Shelton E, Sharma R, and Bass EB, *Impact of Consumer Health Informatics Applications, Evidence Report/Technology Assessment No. 188.,*, Agency for Healthcare Research and Quality, Editor. 2009, Agency for Healthcare Research and Quality,: Rockville, MD.
111. Wright JH, Wright AS, Albano AM, Basco MR, Goldsmith LJ, Raffield T, and Otto MW. Computer-assisted cognitive therapy for depression: maintaining efficacy while reducing therapist time. *Am J Psychiatry,* 2005. 162(6): p. 1158-64.
112. Marsch LA and Dallery J. Advances in the psychosocial treatment of addiction: the role of technology in the delivery of evidence-based psychosocial treatment. *Psychiatr Clin North Am,* 2012. 35(2): p. 481-93.
113. Marks I and Cavanagh K. Computer-aided psychological treatments: Evolving issues. *Annual Review of Clinical Psychology,* 2009. 5: p. 121-141.
114. Horvath AO and Luborsky L. The role of the therapeutic alliance in psychotherapy. *Journal of Consulting and Clinical Psychology,* 1993. 61: p. 561-573.
115. Collins LM, Chakraborty B, Murphy SA, and Strecher V. Comparison of a phased experimental approach and a single randomized clinical trial for developing multicomponent behavioral interventions. *Clin Trials,* 2009. 6(1): p. 5-15.
116. Collins LM, Murphy SA, and Stretcher V. The Multiphase Optimization Strategy (MOST) and the Sequential Multiple Assignment Randomized Trial (SMART): New methods for more potent e-health interventions. *American Journal of Preventive Medicine,* 2007. 32: p. S112-S118. PMCID: PMC2062525.
117. Kiluk BD, Sugarman D, Nich C, Gibbons CJ, Martino S, Rounsaville BJ, and Carroll KM. A methodological analysis of randomized clinical trials of computer-assisted therapies for psychiatric disorders: Towards improved standards for an emerging field. *American Journal of Psychiatry,* 2011. 168: p. 790-799. PMCID: *PMC3607199.*
118. Khadjesari Z, Murray E, Hewitt C, Hartley S, and Godfrey C. Can stand-alone computer-based interventions reduce alcohol consumption? A systematic review. *Addiction,* 2011. 106(2): p. 267-82.

119. Carroll KM, Ball SA, Martino S, Nich C, Babuscio T, Gordon MA, Portnoy GA, and Rounsaville BJ. Computer-assisted cognitive-behavioral therapy for addiction. A randomized clinical trial of 'CBT4CBT'. *American Journal of Psychiatry,* 2008. 165(7): p. 881-888.
120. Carroll KM. *A Cognitive-Behavioral Approach: Treating Cocaine Addiction*. Therapy Manuals for Drug Addiction. 1998, Rockville, Maryland: NIDA.
121. Carroll KM, Nich C, and Ball SA. Practice makes progress: Homework assignments and outcome in the treatment of cocaine dependence. *Journal of Consulting and Clinical Psychology,* 2005. 73: p. 749-755. PMCID: PMC2365906.
122. Carroll KM, Kiluk BD, Nich C, Gordon MA, Portnoy G, Marino D, and Ball SA. Computer-assisted delivery of cognitive-behavioral therapy: Efficacy and durability of CBT4CBT among cocaine-dependent individuals maintained on methadone. *American Journal of Psychiatry,* 2014. 171: p. 436-444.
123. Carroll KM, Ball SA, Martino S, Nich C, Babuscio TA, and Rounsaville BJ. Enduring effects of a computer-assisted training program for cognitive behavioral therapy: A 6-month follow-up of CBT4CBT. *Drug and Alcohol Dependence,* 2009. 100(1): p. 178-181.
124. Longabaugh R. Commentary on Kiluk et al. (2010): the emperor has some new clothes - coping is a mediator of cognitive-behavioral therapy's effectiveness. *Addiction,* 2010. 105(12): p. 2128-9.
125. Kadden R, Carroll KM, Donovan D, Cooney JL, Monti P, Abrams D, Litt M, and Hester RK. *Cognitive-behavioral Coping Skills Therapy Manual: A Clinical Research Guide for Therapists Treating Individuals with Alcohol Abuse and Dependence*. Project MATCH Monograph Series Volume 3. 1992, Rockville, MD: NIAAA.
126. Jones B, Jarvis P, Lewis JA, and Ebbutt AF. Trials to assess equivalence: The importance of rigorous methods. *British Medical Journal,* 1996. 313: p. 36-39.
127. LeHenanff A, Giraudeau B, Baron G, and Ravaud P. Quality of reporting of non-inferiority and equivalence randomized trials. *JAMA,* 2006. 295: p. 1147-1151.
128. Scott IA. Non-inferiority trials: determining whether alternative treatments are good enough. *Medical Journal of Australia,* 2009. 190(6): p. 326-330.
129. Doss BD. Changing the way we study change in psychotherapy. *Clinical Psychology: Science and Practice,* 2004. 11(4): p. 368-386.
130. Marsch LA, Carroll KM, and Kiluk BD. Technology based interventions for the treatment and recovery management of substance use disorders: A JSAT special issue. *Journal of Substance Abuse Treatment,* 2014. 46(1): p. 1-4.
131. Kiluk BD and Carroll KM. New developments in behavioral treatments for substance use disorders. *Current Psychiatry Reports,* 2013. 15: p. 420-429. PMCID: *PMC3878068.*
132. Carroll KM and Rounsaville BJ. Computer-assisted therapy in psychiatry: be brave-it's a new world. *Curr Psychiatry Rep,* 2010. 12(5): p. 426-32.
133. Carroll KM and Rounsaville BJ. A perfect platform: combining contingency management with medications for drug abuse. *American Journal of Drug and Alcohol Abuse,* 2007. 33(3): p. 343-65. PMCID: PMC2367002.
134. Carroll KM. Manual guided psychosocial treatment: A new virtual requirement for pharmacotherapy trials? *Archives of General Psychiatry,* 1997. 54: p. 923-928.

135. Carroll KM, Nich C, Ball SA, McCance-Katz EF, Frankforter TF, and Rounsaville BJ. One year follow-up of disulfiram and psychotherapy for cocaine-alcohol abusers: Sustained effects of treatment. *Addiction,* 2000. 95: p. 1335-1349.
136. Carroll KM, Connors GJ, Cooney NL, DiClemente CC, Donovan DM, Longabaugh RL, Kadden RM, Rounsaville BJ, Wirtz PW, and Zweben A. Internal validity of Project MATCH treatments: Discriminability and integrity. *Journal of Consulting and Clinical Psychology,* 1998. 66: p. 290-303.
137. Carroll KM, Nich C, Ball SA, McCance-Katz E, and Rounsaville BJ. Treatment of cocaine and alcohol dependence with psychotherapy and disulfiram. *Addiction,* 1998. 93: p. 713-728.
138. Carroll KM, Fenton LR, Ball SA, Nich C, Frankforter TL, Shi J, and Rounsaville BJ. Efficacy of disulfiram and cognitive-behavioral therapy in cocaine-dependent outpatients: A randomized placebo controlled trial. *Archives of General Psychiatry,* 2004. 64: p. 264-272.
139. Carroll KM, Rounsaville BJ, Gordon LT, Nich C, Jatlow PM, Bisighini RM, and Gawin FH. Psychotherapy and pharmacotherapy for ambulatory cocaine abusers. *Archives of General Psychiatry,* 1994. 51: p. 177-197.
140. Carroll KM, Rounsaville BJ, Nich C, Gordon LT, Wirtz PW, and Gawin FH. One year follow-up of psychotherapy and pharmacotherapy for cocaine dependence: Delayed emergence of psychotherapy effects. *Archives of General Psychiatry,* 1994. 51: p. 989-997.
141. Carroll KM, Rounsaville BJ, and Gawin FH. A comparative trial of psychotherapies for ambulatory cocaine abusers: Relapse prevention and interpersonal psychotherapy. *American Journal of Drug and Alcohol Abuse,* 1991. 17: p. 229-247.
142. Carroll KM, Easton CJ, Nich C, Hunkele KA, Neavins TM, Sinha R, Ford HL, Vitolo SA, Doebrick CA, and Rounsaville BJ. The use of contingency management and motivational/skills-building therapy to treat young adults with marijuana dependence. *Journal of Consulting and Clinical Psychology,* 2006. 74(5): p. 955-966. PMCID: PMC2148500.
143. Carroll KM, Nich C, LaPaglia DM, Peters EN, Easton CJ, and Petry NM. Combining cognitive behavioral therapy and contingency management to enhance their effects in treating cannabis dependence: less can be more, more or less. *Addiction,* 2012. 107(9): p. 1650-1659.
144. Santa Ana EJ, Martino S, Ball SA, Nich C, Frankforter TL, and Carroll KM. What is usual about 'treatment-as-usual'? Data from two multisite effectiveness trials. *Journal of Substance Abuse Treatment,* 2008. 35(4): p. 369-379.
145. Carroll KM, Nich C, and Rounsaville BJ. Use of observer and therapist ratings to monitor delivery of coping skills treatment for cocaine abusers: Utility of therapist session checklists. *Psychotherapy Research,* 1998. 8: p. 307-320.
146. Martino S, Ball SA, Nich C, Frankforter TL, and Carroll KM. Community program therapist adherence and competence in motivational enhancement therapy. *Drug and Alcohol Dependence,* 2008. 96(1-2): p. 37-48.
147. Gibbons CJ, Nich C, Steinberg K, Roffman RA, Corvino J, Babor TF, and Carroll KM. Treatment process, alliance and outcome in brief versus extended treatments for marijuana dependence. *Addiction,* 2010. 105(10): p. 1799-808.

APPROVED BY THE YALE UNIVERSITY IRB 2/15/2021

148. Carroll KM, Nich C, and Rounsaville BJ. Contribution of the therapeutic alliance to outcome in active versus control psychotherapies. *Journal of Consulting and Clinical Psychology,* 1997. 65: p. 510-514.
149. Carroll KM, Kiluk BD, Nich C, DeVito EE, Decker S, LaPaglia D, Duffey D, Babuscio TA, and Ball SA. Towards empirical identification of a clinically meaningful indicator of treatment outcome for drug addiction: Features of candidate indicators and evaluation of sensitivity to treatment effects and relationship to one year cocaine use follow-up outcomes. *Drug and Alcohol Dependence,* 2014. 137: p. 3-19.
150. Kiluk BD, Nich C, Witkiewitz K, Babuscio TA, and Carroll KM. What happens in treatment doesn't stay in treatment: Cocaine abstinence during treatment is associated with fewer problems at follow-up. *J Consult Clin Psychol,* 2014. 82: p. 619-627. PMCID: *PMC4115028.*
151. MacKinnon DP, Fritz MS, Williams J, and Lockwood CM. Distribution of the product confidence limits for the indirect effect: program PRODCLIN. *Behav Res Methods,* 2007. 39(3): p. 384-9.
152. Carroll KM, Kiluk BD, Nich C, Babuscio TA, Brewer JA, Potenza MN, Ball SA, Martino S, Rounsaville BJ, and Lejuez CW. Cognitive function and treatment response in a randomized clinical trial of computer-based training in cognitive-behavioral therapy. *Subst Use Misuse,* 2011. 46(1): p. 23-34.
153. Kiluk BD, Nich C, and Carroll KM. Relationship of cognitive function and the acquisition of coping skills in computer assisted treatment for substance use disorders. *Drug & Alcohol Dependence,* 2011. 114: p. 169-176. PMCID: *PMC3046302.*
154. Zachary RA. *Shipley Institute of Living Scale: Revised Manual*. 1986, Los Angeles: Western Psychological Services.
155. Connors CK. *The Connors Continuous Performance Test II*. 2004, North Tonawanda, NY: Multi-Health Systems.
156. Buckman JF, Bates ME, and Cisler RA. Social networks and their influence on drinking behaviors: differences related to cognitive impairment in clients receiving alcoholism treatment. *J Stud Alcohol Drugs,* 2007. 68(5): p. 738-47.
157. Bates ME, Pawlak AP, Tonigan JS, and Buckman JF. Cognitive impairment influences drinking outcome by altering therapeutic mechanisms of change. *Psychol Addict Behav,* 2006. 20(3): p. 241-53.
158. Horvath AO and Greenberg LS. Development and validation of the Working Alliance Inventory. *Journal of Counseling Psychology,* 1989. 36(2): p. 223-233.
159. Kiluk BD, Serafini K, Frankforter T, Nich C, and Carroll KM. Only connect: The working alliance in computer-based cognitive behavioral therapy. *Behaviour Research & Therapy,* 2014. 63: p. 139-146. PMCID: *PMC4408209.*
160. Falk D, Wang XQ, Liu L, Fertig J, Mattson M, Ryan M, Johnson B, Stout R, and Litten RZ. Percentage of subjects with no heavy drinking days: Evaluation as an efficacy endpoint for alcoholclinical trials. *Alcoholism: Clinical and Experimental Research,* 2010. 34(12): p. 2022-2034.
161. DiClemente CC, Carbonari JP, Montgomery RP, and Hughes SO. The Alcohol Abstinence Self-Efficacy scale. *J Stud Alcohol,* 1994. 55(2): p. 141-8.

APPROVED BY THE YALE UNIVERSITY IRB 2/15/2021

162. McHugh RK, Kertz SJ, Weiss RB, Baskin-Sommers AR, Hearon BA, and Bjorgvinsson T. Changes in distress intolerance and treatment outcome in a partial hospital setting. *Behav Ther,* 2014. 45(2): p. 232-40.
163. Magill M, Kiluk BD, McCrady BS, Tonigan JS, and Longabaugh R. Active ingredients of treatment and client mechanisms of change in behavioral treatments for alcohol use disorders: Progress 10 years later. *Alcoholism: Clinical & Experimental Research,* under review.
164. Carroll KM, Kadden R, Donovan D, Zweben A, and Rounsaville BJ. Implementing treatment and protecting the validity of the independent variable in treatment matching studies. *Journal of Studies on Alcohol,* 1994. Suppl 12: p. 149-155.
165. Carroll KM, Nich C, Shi JM, Eagan D, and Ball SA. Efficacy of disulfiram and Twelve Step Facilitation in cocaine-dependent individuals maintained on methadone: a randomized placebo-controlled trial. *Drug Alcohol Depend,* 2012. 126(1-2): p. 224-31.
166. Olmstead TA, Sindelar JL, Easton CJ, and Carroll KM. The cost-effectiveness of four treatments for marijuana dependence. *Addiction,* 2007.
167. Dawson DA. Defining risk drinking. *Alcohol Res Health,* 2011. 34(2): p. 144-56.
168. NIAAA, *Helping patients who drink too much: A clinician's guide*, US Department of Health and Human Services, Editor. 2005, National Institutes of Health, National Institute on Alcohol Abuse and Alcoholism.
169. Ray LA, Courtney KE, Ghahremani DG, Miotto K, Brody A, and London ED. Varenicline, low dose naltrexone, and their combination for heavy-drinking smokers: human laboratory findings. *Psychopharmacology (Berl),* 2014. 231(19): p. 3843-53.
170. Martino S, Carroll KM, Nich C, and Rounsaville BJ. A randomized controlled pilot study of motivational interviewing for patients with psychotic and drug use disorders. *Addiction,* 2006. 101(10): p. 1479-92.
171. Ball SA, Martino S, Nich C, Frankforter TL, Van Horn D, Crits-Christoph P, Woody GE, Obert JL, Farentinos C, and Carroll KM. Site matters: Multisite randomized trial of motivational enhancement therapy in community drug abuse clinics. *Journal of Consulting & Clinical Psychology,* 2007. 75(4): p. 556-567. PMCID: PMC2148493.
172. Carroll KM, Martino S, Ball SA, Nich C, Frankforter T, Anez LM, Paris M, Suarez-Morales L, Szapocznik J, Miller WR, Rosa C, Matthews J, and Farentinos C. A multisite randomized effectiveness trial of motivational enhancement therapy for Spanish-speaking substance users. *Journal of Consulting and Clinical Psychology,* 2009. 77(5): p. 993-999.
173. Stout RL, Wirtz PW, Carbonari JP, and DelBoca FK. Ensuring balanced distribution of prognostic factors in treatment outcome research. *Journal of Studies on Alcohol,* 1994. Supplement 12: p. 70-75.
174. Wei LJ. An application of an urn model to the design of sequential controlled clinical trials. *Journal of the American Statistical Association,* 1978. 73: p. 559-563.
175. Carroll KM, Sinha R, Nich C, Babuscio T, and Rounsaville BJ. Contingency management to enhance naltrexone treatment of opioid dependence: A randomized clinical trial of reinforcement magnitude. *Experimental and Clinical Psychopharmacology,* 2002. 10: p. 54-63.

176. MTP Research Group. Brief treatments for cannabis dependence: Findings from a randomized multisite trial. *Journal of Consulting and Clinical Psychology,* 2004. 72: p. 455-466.
177. Petry NM, Roll JM, Rounsaville BJ, Ball SA, Stitzer M, Peirce JM, Blaine J, Kirby KC, McCarty D, and Carroll KM. Serious adverse events in randomized psychosocial treatment studies: safety or arbitrary edicts? *J Consult Clin Psychol,* 2008. 76(6): p. 1076-82.
178. Twitchell GR, Hertzog CA, Klein JL, and Schuckit MA. The anatomy of a follow-up. *British Journal of Addiction,* 1992. 87: p. 1327-1333.
179. Nich C and Carroll KM. Now you see it, now you don't: A practical demonstration of random regression versus traditional ANOVA models in the analysis of longitudinal follow-up data from a clinical trial. *Journal of Consulting and Clinical Psychology,* 1997. 65: p. 252-261.
180. Nich C and Carroll KM. Intention to treat meets missing data: Implications of alternate strategies for analyzing clinical trials data. *Drug and Alcohol Dependence,* 2002. 68: p. 121-130.
181. Kline-Simon AH, Falk DE, Litten RZ, Mertens JR, Fertig J, Ryan M, and Weisner CM. Posttreatment low-risk drinking as a predictor of future drinking and problem outcomes among individuals with alcohol use disorders. *Alcohol Clin Exp Res,* 2013. 37 Suppl 1: p. E373-80.
182. Udo T, Clifford PR, Davis CM, and Maisto SA. Alcohol use post AUD treatment initiation as a predictor of later functioning. *Am J Drug Alcohol Abuse,* 2009. 35(3): p. 128-32.
183. Carroll KM. Constrained, confounded, and confused: Why we know so little about therapist effects. *Addiction,* 2001. 96: p. 203-206.
184. Martino S, Ball S, Nich C, Frankforter TL, and Carroll KM. Correspondence of motivational enhancement treatment integrity ratings among therapists, supervisors, and observers. *Psychother Res,* 2009. 19(2): p. 181-93.
185. Carroll KM, Nuro KF, Rounsaville BJ, and Petrakis I. *Compliance Enhancement: A Clinician's Manual for Pharmacotherapy Trials in the Addictions*. 1998: Unpublished manual: Yale University Psychotherapy Development Center.
186. Volpicelli JR, Pettinati HM, McLellan AT, and O'Brien CP. *Combining medication and psychosocial treatments for addictions: The BRENDA approach*. 2001, New York: Guilford.
187. Pettinati HM, Weiss RD, Miller WR, Donovan DM, and Rounsaville BJ. *Medical Management (MM) Treatment Manual: A Guide for Medically-Trained Clinicians Providing Pharmacotherapy as Part of the Treatment of Alcohol Dependence*. 2004, Bethesda, MD: NIAAA.
188. Pettinati HM, Weiss RD, Dundon W, Miller WR, Donovan D, Ernst DB, and Rounsaville BJ. A structured approach to medical management: a psychosocial intervention to support pharmacotherapy in the treatment of alcohol dependence. *J Stud Alcohol Suppl,* 2005(15): p. 170-8; discussion 168-9.
189. Kenwright M, Marks IM, Graham C, Franses A, and Mataix-Cols D. Brief scheduled phone support from a clinician to enhance computer-aided self-help for obsessive-compulsive disorder: Randomized clinical trial. *Journal of Clinical Psychology,* 2005. 61(12): p. 1499-1508.

APPROVED BY THE YALE UNIVERSITY IRB 2/15/2021

190. Martino S, Ball SA, Nich C, Frankforter TL, and Carroll KM. Informal discussions in substance abuse treatment sessions. *J Subst Abuse Treat,* 2009. 36(4): p. 366-75.
191. Piaggio G, Elbourne DR, Altman DG, Pocock SJ, and Evans SJW. Reporting of noninferiority and equivalence randomized trials: An extension of the CONSORT statement. *JAMA,* 2006. 295(1152-1160).
192. Hamilton CM, Strader LC, Pratt JG, Maiese D, Hendershot T, Kwok RK, Hammond JA, Huggins W, Jackman D, Pan H, Nettles DS, Beaty TH, Farrer LA, Kraft P, Marazita ML, Ordovas JM, Pato CN, Spitz MR, Wagener D, Williams M, Junkins HA, Harlan WR, Ramos EM, and Haines J. The PhenX Toolkit: get the most from your measures. *Am J Epidemiol,* 2011. 174(3): p. 253-60.
193. Hendershot T, Pan H, Haines J, Harlan WR, Junkins HA, Ramos EM, and Hamilton CM. Using the PhenX Toolkit to Add Standard Measures to a Study. *Curr Protoc Hum Genet,* 2011. Chapter 1: p. Unit1 21.
194. Fucito LM, Park A, Gulliver SB, Mattson ME, Gueorguieva RV, and O'Malley SS. Cigarette smoking predicts differential benefit from naltrexone for alcohol dependence. *Biol Psychiatry,* 2012. 72(10): p. 832-8.
195. Deveci SE, Deveci F, Acik Y, and Ozan AT. The measurement of exhaled carbon monoxide in healthy smokers and non-smokers. *Respir Med,* 2004. 98(6): p. 551-6.
196. Gastfriend DR, Garbutt JC, Pettinati HM, and Forman RF. Reduction in heavy drinking as a treatment outcome in alcohol dependence. *J Subst Abuse Treat,* 2007. 33(1): p. 71-80.
197. Jackson KM. Heavy episodic drinking: determining the predictive utility of five or more drinks. *Psychol Addict Behav,* 2008. 22(1): p. 68-77.
198. Donovan DM, Bigelow GE, Brigham GS, Carroll KM, Cohen AJ, Gardin JG, Hamilton JA, Huestis MA, Hughes JR, Lindblad R, Marlatt GA, Preston KL, Selzer JA, Somoza EC, Wakim PG, and Wells EA. Primary outcome indices in illicit drug dependence treatment research: Systematic approach to selection and measurement of drug use endpoints in clinical trials. *Addiction,* 2012. 107(4): p. 694-708.
199. Jatlow P and O'Malley SS. Clinical (nonforensic) application of ethyl glucuronide measurement: are we ready? *Alcohol Clin Exp Res,* 2010. 34(6): p. 968-75.
200. Sarkola T, Dahl H, Eriksson CJ, and Helander A. Urinary ethyl glucuronide and 5-hydroxytryptophol levels during repeated ethanol ingestion in healthy human subjects. *Alcohol Alcohol,* 2003. 38(4): p. 347-51.
201. Wurst FM, Wiesbeck GA, Metzger JW, and Weinmann W. On sensitivity, specificity, and the influence of various parameters on ethyl glucuronide levels in urine--results from the WHO/ISBRA study. *Alcohol Clin Exp Res,* 2004. 28(8): p. 1220-8.
202. Anton RF. Commentary on: ethyl glucuronide and ethyl sulfate assays in clinical trials, interpretation, and limitations: results of a dose ranging alcohol challenge study and 2 clinical trials. *Alcohol Clin Exp Res,* 2014. 38(7): p. 1826-8.
203. Chaney EF, O'Leary MR, and Marlatt GA. Skill training with problem drinkers. *Journal of Consulting and Clinical Psychology,* 1978. 46: p. 1092-1104.
204. Fenton LR, Cecero JJ, Nich C, Frankforter TL, and Carroll KM. Perspective is everything: The predictive validity of six working alliance instruments. *Journal of Psychotherapy Practice and Research,* 2001. 10: p. 262-268.

APPROVED BY THE YALE UNIVERSITY IRB 2/15/2021

APPROVED BY THE YALE UNIVERSITY IRB 2/15/2021

205. Connors GJ, Carroll KM, DiClemente CC, Longabaugh R, and Donovan DM. The therapeutic alliance and its relationship to alcoholism treatment participation and outcome. *Journal of Consulting and Clinical Psychology,* 1997. 65: p. 588-598.
206. Magrys SA and Olmstead MC. Alcohol intoxication alters cognitive skills mediated by frontal and temporal brain regions. *Brain Cogn,* 2014. 85: p. 271-6.
207. Naim-Feil J, Fitzgerald PB, Bradshaw JL, Lubman DI, and Sheppard D. Neurocognitive deficits, craving, and abstinence among alcohol-dependent individuals following detoxification. *Arch Clin Neuropsychol,* 2014. 29(1): p. 26-37.
208. Townshend JM and Duka T. Binge drinking, cognitive performance and mood in a population of young social drinkers. *Alcohol Clin Exp Res,* 2005. 29(3): p. 317-25.
209. Smith JL, Mattick RP, Jamadar SD, and Iredale JM. Deficits in behavioural inhibition in substance abuse and addiction: A meta-analysis. *Drug Alcohol Depend,* 2014.
210. Beatty WW, Katzung VM, Moreland VJ, and Nixon SJ. Neuropsychological performance of recently abstinent alcoholics and cocaine abusers. *Drug Alcohol Depend,* 1995. 37(3): p. 247-53.
211. Goldstein RZ, Leskovjan AC, Hoff AL, Hitzemann R, Bashan F, Khalsa SS, Wang G-J, Fowler JS, and Volkow ND. Severity of neuropsychological impairment in cocaine and alcohol addiction: association with metabolism in the prefrontal cortex. *Neuropsychologia,* 2004. 42(11): p. 1447-1458.
212. Flannery B, Fishbein D, Krupitsky E, Langevin D, Verbitskaya E, Bland C, Bolla K, Egorova V, Bushara N, Tsoy M, and Zvartau E. Gender differences in neurocognitive functioning among alcohol-dependent Russian patients. *Alcohol Clin Exp Res,* 2007. 31(5): p. 745-54.
213. Parsons OA. Neurocognitive deficits in alcoholics and social drinkers: a continuum? *Alcohol Clin Exp Res,* 1998. 22(4): p. 954-61.
214. Houston RJ, Derrick JL, Leonard KE, Testa M, Quigley BM, and Kubiak A. Effects of heavy drinking on executive cognitive functioning in a community sample. *Addict Behav,* 2014. 39(1): p. 345-9.
215. Noel X, Bechara A, Dan B, Hanak C, and Verbanck P. Response inhibition deficit is involved in poor decision making under risk in nonamnesic individuals with alcoholism. *Neuropsychology,* 2007. 21(6): p. 778-86.
216. Rounsaville DB, Hunkele K, Easton CJ, Nich C, and Carroll KM. Making consent more informed: preliminary results from a multiple-choice test among probation-referred marijuana users entering a randomized clinical trial. *J Am Acad Psychiatry Law,* 2008. 36(3): p. 354-9.
217. Kiluk BD, Nich C, and Carroll KM. Neurocognitive indicators predict results of an informed consent quiz among substance dependent treatment seekers entering a randomized clinical trial. *Journal of Studies on Alcohol and Drugs,* 2010. 71: p. 704-712. PMCID: *PMC2930500.*
218. Kranzler HR, Kadden RM, Babor TF, Tennen H, and Rounsaville BJ. Validity of the SCID in substance abuse patients. *Addiction,* 1996. 91: p. 859-868.
219. Derogatis LR and Melisaratos N. The Brief Symptom Index: An introductory report. *Psychological Medicine,* 1983. 13: p. 595-605.
220. Anton RF, Moak DH, and Latham P. The Obsessive Compulsive Drinking Scale: A self-rated instrument for the quantification of thoughts about alcohol and drinking behavior. *Alcoholism: Clinical and Experimental Research,* 1995. 19(1): p. 92-99.

APPROVED BY THE YALE UNIVERSITY IRB 2/15/2021

APPROVED BY THE YALE UNIVERSITY IRB 2/15/2021

221. Wurst FM, Dresen S, Allen JP, Wiesbeck G, Graf M, and Weinmann W. Ethyl sulphate: a direct ethanol metabolite reflecting recent alcohol consumption. *Addiction,* 2006. 101(2): p. 204-11.
222. World Health Organization Quality of Life Group. Development of the World Health Organization WHOQOL-BREF quality of life instrument. *Psychol Med,* 1998. 28(3): p. 551-558.
223. Bohn MJ, Babor TF, and Kranzler HR. The Alcohol Use Disorders Identification Test (AUDIT): validation of a screening instrument for use in medical settings. *J Stud Alcohol,* 1995. 56(4): p. 423-32.
224. Saunders JB, Aasland OG, Babor TF, de la Fuente JR, and Grant M. Development of the Alcohol Use Disorders Identification Test (AUDIT): WHO Collaborative Project on Early Detection of Persons with Harmful Alcohol Consumption--II. *Addiction,* 1993. 88(6): p. 791-804.
225. Dawson DA, Grant BF, Stinson FS, and Zhou Y. Effectiveness of the derived Alcohol Use Disorders Identification Test (AUDIT-C) in screening for alcohol use disorders and risk drinking in the US general population. *Alcohol Clin Exp Res,* 2005. 29(5): p. 844-54.
226. Rubinsky AD, Dawson DA, Williams EC, Kivlahan DR, and Bradley KA. AUDIT-C scores as a scaled marker of mean daily drinking, alcohol use disorder severity, and probability of alcohol dependence in a U.S. general population sample of drinkers. *Alcohol Clin Exp Res,* 2013. 37(8): p. 1380-90.
227. Rubinsky AD, Kivlahan DR, Volk RJ, Maynard C, and Bradley KA. Estimating risk of alcohol dependence using alcohol screening scores. *Drug Alcohol Depend,* 2010. 108(1-2): p. 29-36.
228. Shipley WC. *Manual: Shipley-Institute of Living Scale*. 1967, Los Angeles: Western Psychological Services.
229. Zachary RA. *The Manual of the Shipley Institute of Living Scale*. 1991, Los Angeles: Western Psychological Services.
230. DiClemente CC and Hughes SO. Stages of change profiles in outpatient alcoholism treatment. *Journal of Substance Abuse,* 1990. 2: p. 217-235.
231. Litt MD, Kadden RM, Stephens RS, Babor TF, Carroll KM, Christiansen K, Donaldson J, Herrell J, McRee B, Miller M, Roffman R, Solowji N, Steinberg K, and Vendetti J. Coping skills and self-efficacy in marijuana treatment: Results from the Marijuana Treatment Project. *Journal of Consulting and Clinical Psychology,* 2005. 73(6): p. 1015-1025.
232. Annis HM and Davis CS. *Assessment of expectancies*, in *Assessment of Addictive Behaviors*, D.M. Donovan and G.A. Marlatt, Editors. 1988, Guilford Press: New York. p. 84-111.
233. Olmstead TA, Sindelar JL, and Petry NM. Cost-effectiveness of prize-based incentives for stimulant abusers in outpatient psychosocial treatment programs. *Drug and Alcohol Dependence,* 2007. 87: p. 175-182.
234. Horvath AO and Greenberg LS. *The development of the Working Alliance Inventory*, in *The Psychotherapeutic Process: A Research Handbook*, L.S. Greenberg and W.M. Pinsof, Editors. 1986, Guilford: New York. p. 529-556.
235. Tracey TJ and Kokotovic AM. Factor structure of the Working Alliance Inventory. *Psychological Assessment,* 1989. 1: p. 207-210.

APPROVED BY THE YALE UNIVERSITY IRB 2/15/2021

APPROVED BY THE YALE UNIVERSITY IRB 2/15/2021

236. Cecero JJ, Fenton LR, Nich C, Frankforter T, and Carroll KM. Focus on the therapeutic alliance: The psychometric properties of six measures across three treatments. *Psychotherapy,* 2001. 38: p. 1-11.
237. Tichenor V and Hill CE. A comparison of six measures of the working alliance. *Psychotherapy,* 1989. 26: p. 195-199.
238. Donovan DM, Kadden RM, DiClemente CC, and Carroll KM. Client satisfaction with three therapies in the treatment of alcohol dependence: Results from Project MATCH. *American Journal on Addictions,* 2002. 11: p. 291-307.
239. Babor TF, Steinberg K, Anton RF, and Del Boca FK. Talk is cheap: Measuring drinking outcomes in clinical trials. *Journal of Studies on Alcohol,* 2000. 61: p. 55-63.
240. Zanis DA, McLellan AT, and Randall M. Can you trust patient self-reports of drug use during treatment? *Drug and Alcohol Dependence,* 1994. 35: p. 127-132.
241. Clifford PR and Davis CM. Alcohol treatment research assessment exposure: a critical review of the literature. *Psychol Addict Behav,* 2012. 26(4): p. 773-81.
242. Clifford PR, Maisto SA, and Davis CM. Alcohol treatment research assessment exposure subject reactivity effects: part I. Alcohol use and related consequences. *J Stud Alcohol Drugs,* 2007. 68(4): p. 519-28.
243. Yates BT. Toward the incorporation of costs, cost-effectiveness analysis, and cost-benefit analysis into clinical research. *J Consult Clin Psychol,* 1994. 62(4): p. 729-36.
244. Yates BT and Taub J. Assessing the costs, benefits, cost-effectiveness, and cost-benefit of psychological assessment: we should, we can, and here's how. *Psychol Assess,* 2003. 15(4): p. 478-95.
245. Fals-Stewart W, Klostermann K, Yates BT, O'Farrell TJ, and Birchler GR. Brief relationship therapy for alcoholism: a randomized clinical trial examining clinical efficacy and cost-effectiveness. *Psychol Addict Behav,* 2005. 19(4): p. 363-71.
246. Yates BT. Cost-inclusive evaluation: a banquet of approaches for including costs, benefits, and cost-effectiveness and cost-benefit analyses in your next evaluation. *Eval Program Plann,* 2009. 32(1): p. 52-4.
247. Yates BT, *Measuring and improving cost, cost-effectiveness, and cost-benefit for substance abuse treatment programs*. 1999, National Institute on Drug Abuse, NIH Publication Number 99-4518: Bethesda, MD.
248. Yates BT. *Cost-benefit and cost-effectiveness analysis*, in *Encyclopedia of Psychology*, A. Kazdin, Editor. 2000, American Psychological Association: Washington, DC.
249. Yates BT. *Program evaluation: Outcomes and costs of putting psychology to work*, in *Handbook of Research Methods in Psychology, Vol 2*, H. Cooper, Editor. 2012, American Psychological Association: Washington, DC.
250. Taxman FS and Yates BT. *Quantitative exploration of Pandora's box of treatment and supervision: What goes on between costs in and outcomes out*, in *Costs and benefits of preventing crime*, B.C. Welsch and D.P. Farrington, Editors. 2001, Westview Press: Boulder, CO. p. 51-84.
251. Muthen LK and Muthen BO, *Statistical Analysis with Latent Variables: Users Guide*. 2012: www.StatModel.com.

APPROVED BY THE YALE UNIVERSITY IRB 2/15/2021

APPROVED BY THE YALE UNIVERSITY IRB 2/15/2021

252. Hayes AF. *Introduction to mediation, moderation, and conditional process analysis: A regression-based approach*. 2013, New York, NY: Guilford Press.
253. Maisto SA, Roos CR, O'Sickey AJ, Kirouac M, Connors GJ, Tonigan JS, and Witkiewitz K. The indirect effect of the therapeutic alliance and alcohol abstinence self-efficacy on alcohol use and alcohol-related problems in Project MATCH. *Alcohol Clin Exp Res,* 2015. 39(3): p. 504-13.
254. Preacher KJ and Hayes AF. Asymptotic and resampling strategies for assessing and comparing indirect effects in multiple mediator models. *Behav Res Methods,* 2008. 40(3): p. 879-91.
255. Petry NM, Roll JM, Rounsaville BJ, Ball SA, Stitzer M, Peirce JM, Blaine J, Kirby KC, McCarty D, and Carroll KM. Serious adverse events in randomized psychosocial treatment studies: safety or arbitrary edicts? *Journal of Consulting and Clinical Psychology,* 2008. 76(6): p. 1076-82, PMCID: PMC2756150.
256. Sampson JP and Pyle KR. Ethical issues involved with the use of computer-assessed counseling, testing, and guidance systems. *Personnel and Guidance Journal,* 1983. 61: p. 283-287.
257. Wright JH and Wright AS. Computer-assisted psychotherapy. *Journal of Psychotherapy Practice and Research,* 1997. 6: p. 315-329.
258. Marks IM, Cuijpers P, Cavanagh K, van Straten A, Gega L, and Andersson G. Meta-analysis of computer-aided psychotherapy: Problems and partial solutions. *Cognitive Behaviour Therapy,* 2009. 38(2): p. 83-90.
259. Cuijpers P, Marks IM, van Straten A, Cavanagh K, Gega L, and Andersson G. Computer-aided psychotherapy for anxiety disorders: A meta-analytic review. *Cognitive Behaviour Therapy,* 2009. 38(2): p. 66-82.
260. Moore BA, Fazzino T, Garnet B, Cutter CJ, and Barry DT. Computer-based interventions for drug use disorders: A systematic review. *Journal of Substance Abuse Treatment,* 2011. 40(3): p. 215-223.
261. McCance-Katz EF, Carroll KM, and Rounsaville BJ. Gender differences in treatment seeking cocaine abusers: Implications for treatment and prognosis. *American Journal on Addictions,* 1999. 8: p. 300-311.
262. Milligan CO, Nich C, and Carroll KM. Ethnic differences in substance abuse treatment retention, compliance, and outcome: Findings from two randomized clinical trials. *Psychiatric Services,* 2004. 55: p. 167-173.
263. Nich C, McCance-Katz EF, Petrakis IL, Cubells JF, Rounsaville BJ, and Carroll KM. Gender differences in cocaine dependent individuals' response to disulfiram treatment. *Addictive Behaviors,* 2004. 29: p. 1123-1129.
264. Montgomery L, Petry NM, and Carroll KM. Moderating effects of race in clinical trial participation and outcomes among marijuana-dependent young adults. *Drug Alcohol Depend,* 2012.
265. DeVito EE, Babuscio T, Nich C, and Carroll KM. Influence of gender on clinical outcomes in randomized clinical trials for cocaine dependence. under review.
266. McCance-Katz EF, Carroll KM, and Rounsaville BJ. Gender differences in treatment-seeking cocaine abusers--implications for treatment and prognosis. *Am J Addict,* 1999. 8(4): p. 300-11.

APPROVED BY THE YALE UNIVERSITY IRB 2/15/2021

267. McHugh RK, Devito EE, Dodd D, Carroll KM, Potter JS, Greenfield SF, Connery HS, and Weiss RD. Gender differences in a clinical trial for prescription opioid dependence. *J Subst Abuse Treat,* 2013.
268. Suarez-Morales L, Matthews J, Martino S, Ball SA, Rosa C, Farentinos C, Szapocznik J, and Carroll KM. Issues in designing and implementing a Spanish-language multi-site clinical trial. *American Journal on the Addictions,* 2007. 16(3): p. 206-215.
269. Carroll KM, Rosa C, Brown LS, Jr., Daw R, Magruder KM, and Beatty L. Addressing ethnic disparities in drug abuse treatment in the Clinical Trials Network. *Drug and Alcohol Dependence,* 2007. 90: p. 101-106.

APPROVED BY THE YALE UNIVERSITY IRB 2/15/2021
