# Supplementary material for: A Digital Cognitive Behavioral Therapy Program for Adults With Alcohol Use Disorder: A Randomized Clinical Trial
Source: JAMA Netw Open. 2024 Sep 26;7(9):e2435205. doi: 10.1001/jamanetworkopen.2024.35205 (PMC11428014; doi:10.1001/jamanetworkopen.2024.35205)
Supplement: Supplement 2. — eTable. Percentage of Days Abstinent from Alcohol (PDA) and Percentage of Heavy Drinking Days (PHDD) at Each Study Time Point [file jamanetwopen-e2435205-s002.pdf]

Supplementary Online Content

Kiluk BD, Benitez B, DeVito EE, et al. A digital cognitive behavioral therapy program for adults with alcohol use disorder: a randomized clinical trial. *JAMA Netw Open*. 2024;7(9):e2435205. doi:10.1001/jamanetworkopen.2024.35205

eTable. Percentage of Days Abstinent from Alcohol (PDA) and Percentage of Heavy Drinking Days (PHDD) at Each Study Time Point

This supplementary material has been provided by the authors to give readers additional information about their work.

© 2024 Kiluk BD et al. *JAMA Network Open.*

eTable. Percentage of Days Abstinent from Alcohol (PDA) and Percentage of Heavy Drinking Days (PHDD) at Each Study Time Point

	TAU		CBT		CBT4CBT	
**Percentage of Days Abstinent (PDA) during past month**	*M (sd)*	n	*M (sd)*	n	*M (sd)*	n
Baseline	49.3 (27.8)	34	53.7 (29.8)	32	47.6 (31.8)	33
Month 1	63.3 (29.8)	31	69.9 (29.4)	31	71.1 (28.2)	30
Month 2	69.3 (26.2)	28	68.1 (29.9)	29	75.1 (25.1)	29
Month 3	66.7 (32.1)	28	75.9 (29.3)	29	74.7 (32.6)	27
Month 4	63.9 (35.6)	25	72.0 (30.9)	27	77.1 (28.6)	26
Month 5	55.1 (37.5)	25	68.1 (33.1)	27	82.6 (22.8)	26
Month 6	67.7 (36.5)	23	63.7 (35.9)	26	82.1 (23.1)	24
Month 7	68.2 (37.2)	23	68.3 (37.1)	26	85.1 (23.7)	24
Month 8	69.6 (34.4)	23	70.2 (35.1)	26	82.6 (25.3)	24
**Percentage of Heavy Drinking Days (PHDD) during past month**	*M (sd)*	n	*M (sd)*	n	*M (sd)*	n
Baseline	68.2 (28.9)	34	67.5 (30.1)	32	61.4 (32.0)	33
Month 1	17.3 (22.0)	31	15.7 (24.2)	31	12.3 (15.7)	30
Month 2	14.7 (22.5)	28	13.5 (21.5)	29	13.3 (22.0)	29
Month 3	13.8 (23.0)	28	12.1 (22.2)	29	13.2 (28.3)	27
Month 4	15.7 (28.6)	25	9.8 (15.9)	27	12.4 (20.7)	26
Month 5	22.3 (31.7)	25	10.6 (20.4)	27	7.1 (11.6)	26
Month 6	16.3 (28.2)	23	18.0 (31.2)	26	14.9 (22.9)	24
Month 7	15.7 (28.3)	23	15.8 (28.9)	26	11.0 (23.6)	24
Month 8	15.8 (26.1)	23	12.6 (26.6)	26	10.4 (21.5)	24

© 2024 Kiluk BD et al. *JAMA Network Open.*
