# Supplementary material for: A Digital Cognitive Behavioral Therapy Program for Adults With Alcohol Use Disorder: A Randomized Clinical Trial
Source: JAMA Netw Open. 2024 Sep 26;7(9):e2435205. doi: 10.1001/jamanetworkopen.2024.35205 (PMC11428014; doi:10.1001/jamanetworkopen.2024.35205)
Supplement: Supplement 3. — Data Sharing Statement [file jamanetwopen-e2435205-s003.pdf]

Data Sharing Statement

Kiluk. A Digital Cognitive Behavioral Therapy Program for Adults With Alcohol Use Disorder. *JAMA Netw Open*. Published September 26, 2024. doi:10.1001/jamanetworkopen.2024.35205

Data
Data available: Yes
Data types: Deidentified participant data
How to access data: brian.kiluk@yale.edu
When available: With publication

Supporting Documents
Document types: None

Additional Information
Who can access the data: researchers whose proposed use of the data has been approved
Types of analyses: for a specified purpose
Mechanisms of data availability: with a signed data access agreement
